# Unique somatic and malignant expression patterns implicate PIWI-interacting RNAs in cancer-type specific biology

**DOI:** 10.1038/srep10423

**Published:** 2015-05-27

**Authors:** Victor D. Martinez, Emily A. Vucic, Kelsie L. Thu, Roland Hubaux, Katey S.S. Enfield, Larissa A. Pikor, Daiana D. Becker-Santos, Carolyn J. Brown, Stephen Lam, Wan L. Lam

**Affiliations:** 1 Department of Integrative Oncology, British Columbia Cancer Research Centre, Vancouver, B.C. V5Z 1L3 Canada; 2 Department of Medical Genetics, University of British Columbia, Vancouver, B. C. V6T 1Z3 Canada

## Abstract

Human PIWI-interacting RNAs (piRNAs) are known to be expressed in germline cells, functionally silencing LINEs and SINEs. Their expression patterns in somatic tissues are largely uncharted. We analyzed 6,260 human piRNA transcriptomes derived from non-malignant and tumour tissues from 11 organs. We discovered that only 273 of the 20,831 known piRNAs are expressed in somatic non-malignant tissues. However, expression patterns of these piRNAs were able to distinguish tissue-of-origin. A total of 522 piRNAs are expressed in corresponding tumour tissues, largely distinguishing tumour from non-malignant tissues in a cancer-type specific manner. Most expressed piRNAs mapped to known transcripts, contrary to “piRNA clusters” reported in germline cells. We showed that piRNA expression can delineate clinical features, such as histological subgroups, disease stages, and survival. PiRNAs common to many cancer types might represent a core gene-set that facilitates cancer growth, while piRNAs unique to individual cancer types likely contribute to cancer-specific biology.

Small RNA-guided gene regulation represents a widely conserved mechanism across almost all living organisms^1^. Small RNA-mediated gene silencing typically involves a sequence recognition particle (i.e. small RNA) and a member of the Argonaute protein family, which is composed of Argonaute (Ago) and P-element–induced wimpy testis (PIWI) subfamilies^2^. Based largely on differences in biogenesis and structure, small RNAs can be broadly divided into three groups: small interfering RNAs (siRNAs), microRNAs (miRNAs) and PIWI-interacting RNA (piRNAs). These interact with either the AGO (siRNAs, miRNAs) or PIWI subfamily (piRNAs) to form gene regulatory RNA/protein complexes^2^,^3^.

The discovery of piRNAs affirmed a fundamental functional role for the small non-protein coding genome^4^,^5^,^6^,^7^,^8^,^9^. piRNAs are small (24–32 nucleotides), single-stranded non-coding RNAs, that have highly conserved functions across species, such as transposon silencing and stem cell maintenance in germline tissues^6^,^10^,^11^,^12^. Today, conservative estimates for the total number of piRNAs in the eukaryotic genome parallel those of protein-coding genes (~20,000), and largely exceeds the estimated 2,000 miRNA loci^13^,^14^. Most piRNAs are derived from long, single-stranded RNA precursors transcribed from distinct transposons referred to as “piRNA clusters”^11^,^15^,^16^; however, a small fraction of piRNAs are also encoded in intergenic noncoding transcripts as well as protein-coding genes, primarily in the 3′ untranslated regions (3′UTRs)^6^,^7^,^10^,^17^,^18^,^19^.

In humans, mature piRNAs form complexes with one of the four PIWI proteins (PIWIL1/HIWI, PIWIL2/HILI, PIWIL3, and PIWIL4/HIWI2). Specificity of the piRNA/PIWI riboprotein complex is mediated by the piRNA sequence which targets the complex to sites of complementary DNA or messenger RNA (mRNA); the effects of which are likely mediated by recruited cofactors, in a tissue- and context-specific manner in different species and for different PIWI proteins (reviewed in^15^,^20^). The piRNA/PIWI complex-mediated transcriptional silencing of specific genomic loci complementary to the piRNA sequence occurs through recruitment of epigenetic machinery and establishment of repressive epigenetic marks^8^,^9^,^21^,^22^,^23^,^24^,^25^,^26^,^27^,^28^. However, piRNAs and PIWI proteins are also found in the cytoplasm, and evidence of piRNA involvement in the regulation of mRNA (i.e., post-transcriptional silencing) is emerging^20^. For example, piRNAs can target the piRNA/PIWI complex to mRNA sequences containing a 3′ retrotransposon sequence^20^,^29^,^30^. PiRNAs can also bind mRNAs in *trans*, when derived from pseudogenes of target mRNAs, or in *cis*, when encoded within endogenous genes^20^,^31^. The mechanisms governing piRNA-mediated mRNA degradation in humans are not well understood, although they may involve both small RNA-dependent endonucleases (slicer) and slicer-independent mechanisms for degradation of target RNAs by PIWI proteins and piRNAs, and recruitment of proteins that mediate mRNA deadenylation^20^,^32^.

The functional role of piRNAs in somatic tissues — from stem cell maintenance, to memory-related synaptic plasticity, to whole body regeneration in diverse species — is a rapidly emerging field of research^8^,^33^,^34^,^35^,^36^. In human cancers, some components of the PIWI biogenesis machinery have been characterized^24^,^37^; but tumour specific expression of piRNAs are limited to either single gene studies^38^,^39^, or expression profiles of a few representative tumours^40^,^41^,^42^ or cell lines^42^,^43^,^44^,^45^ for a few types of cancers.

Given the abundant presence of piRNAs in our genomes, the functional conservation of piRNAs across species, their functions in development, and the important physiological roles of even the small number of piRNAs studied to date, further exploration of these molecules in a broad spectrum of normal and diseased somatic tissues is warranted in order to gain further insights into normal and disease-related biology^46^. Towards this aim, we systematically assessed piRNA transcriptomes in 6,260 human tissue samples derived from non-malignant and tumour tissues from multiple organs. We investigated: 1) which piRNAs are expressed in somatic tissues? 2) are they the same ones expressed in germline cells? 3) does piRNA expression follow a tissue-specific pattern? 4) are piRNA expression patterns altered in cancer, and in a tumour-type specific manner? and 5) are piRNA expression patterns related to clinical features important to specific cancer types?

## Results

### piRNAs are expressed across non-malignant human somatic tissues in a shared and tissue-specific manner

To date, piRNA expression has been primarily described in germline tissues, thus the first goal of this study was to systematically assess piRNA transcriptome patterns across human somatic tissues (Table 1). Briefly, we evaluated the expression of 20,831 unique piRNAs (encoded by 39,545 genomic loci, see Supplementary Table 1) in normal tissues (n = 508) derived from 10 different anatomical sites: bladder, breast, colon, head and neck (different sites), kidney, lung, prostate, stomach, thyroid, and uterine corpus available from The Cancer Genome Atlas (TCGA) consortium. We detected 273 piRNAs expressed in somatic, non-malignant tissues (defined as Reads per kilobase per million mapped reads (RPKM) ≥ 1 in at least 10% of samples). While the number of piRNAs we detect as somatically expressed is small (1.3%) relative to the total pool of piRNAs assessed, our results indicate that piRNAs are expressed in all somatic, non-malignant tissues analyzed. RPKM expression values for all piRNAs are listed in Supplementary Table 2.

**Figure 1:**
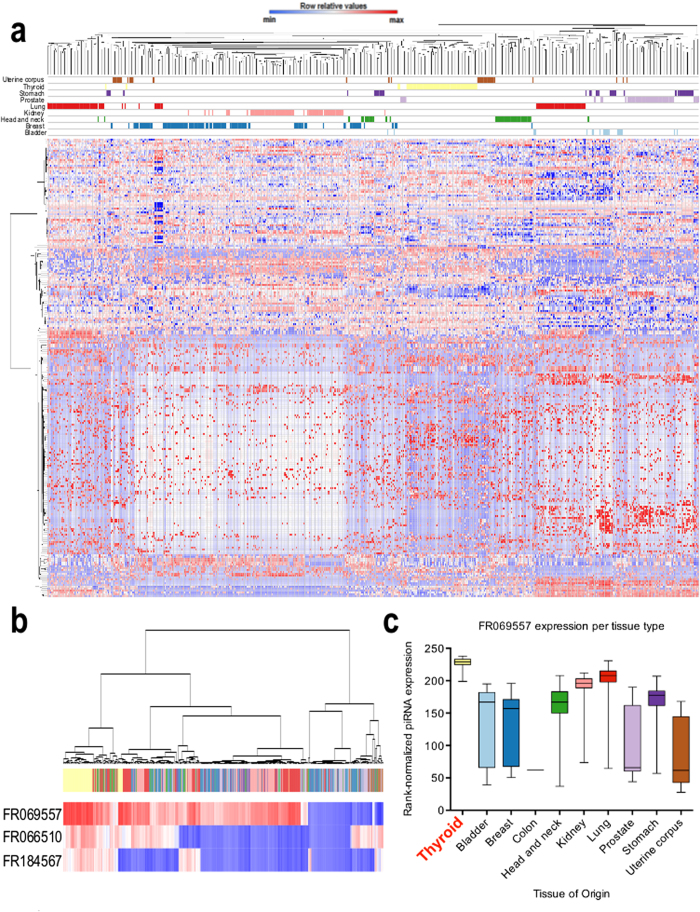
Genome-wide distribution of piRNA expression in human non-malignant tissues. Colour coding for somatic tissues noted in Fig. 1A applies to all panels (a–c). **a**) Unsupervised hierarchical clustering of rank-normalized piRNA expression levels from 508 non-malignant tissue samples derived from 10 different organs: bladder, breast, colon, head and neck, kidney, lung, prostate, stomach, thyroid and uterine corpus. Tissue types are colour coded and separated by rows under the dendrogram. A total of 273 piRNAs were expressed in non-malignant tissues. Expression levels are coded from low (blue) to high (red) on a row-standardized scale, meaning that expression values (colours) are comparable both across rows. The single colon non-malignant sample analyzed is not shown in this heatmap, to avoid misinterpretation due to a low sample size **b**) Top three differentially expressed piRNAs that can discriminate between thyroid and other tissues based on a comparative marker selection analysis^73^. Unsupervised hierarchical clustering was also performed to display the ability to distinguish thyroid (yellow) from other tissues. **c**) Box-and-whiskers plots of normalized expression levels of FR069557 across different non-malignant tissue types.

We observed intriguing piRNA expression patterns across non-malignant somatic tissues (Fig. 1a). Overall, piRNA expression patterns appeared similar across all samples for both highly and lowly expressed piRNAs. We stress that only one non-malignant colon tissue was available for comparison and therefore colorectal cancer piRNA expression results are likely less robust than for other cancer types. However, when tissue-origin information was overlaid onto the sample dendrogram (denoted by colored bars on top of the heatmap in Fig. 1a), tissue-specific piRNA expression patterns for multiple organs were evident, particularly for thyroid and prostate tissues. Other tissues clustered together moderately (e.g., lung, head and neck or bladder) or were highly heterogeneous with samples clustering with many different tissue types (e.g., stomach and bladder). piRNAs highly expressed across all tissues could suggest a conserved general function for these piRNAs in somatic tissues, while subtle differences in piRNAs expressed at low or moderate levels may be capable of distinguishing tissues based on organ type, possibly indicating a tissue-specific role for these piRNAs, which would be consistent with the literature on somatic piRNAs^8^.

For thyroid—the most homogenously clustered tissue type—as few as three piRNAs (FR069557, FR066510 and FR184567) were capable of distinguishing a high proportion of thyroid tissues from all other tissue types (Fig. 1b). Expression of FR069557 (the top ranked differentially expressed piRNA between thyroid and all other tissues analyzed) was significantly higher in thyroid compared to all other individual tissues (p value < 0.0001, one-way ANOVA, Fig. 1c). Tissue specific expression of piRNAs could implicate a specific functional role for such piRNAs in various somatic tissues.

### piRNA expression distinguishes non-malignant and tumour tissues

To identify piRNAs that differentiate non-malignant and tumour tissues, we next analyzed piRNA expression in tumour samples (n = 5,752) from 12 different tumour types: bladder urothelial carcinoma (BLCA), breast invasive carcinoma (BRCA), colon adenocarcinoma (COAD), head and neck squamous cell carcinoma (HNSC), kidney renal clear cell (KIRC), lung adenocarcinoma (LUAD), lung squamous cell carcinoma (LUSC), ovarian serous cystadenocarcinoma (OV), prostate adenocarcinoma (PRAD), stomach adenocarcinoma (STAD), thyroid carcinoma (THCA), and uterine corpus (UCEC). Consistent with a putative role for piRNAs in promoting cell proliferation, we detected a considerably higher number of piRNAs expressed in tumours (n = 522 piRNAs, encoded at 635 piRNA loci) relative to non-malignant tissues (n = 273 piRNAs).

**Figure 2:**
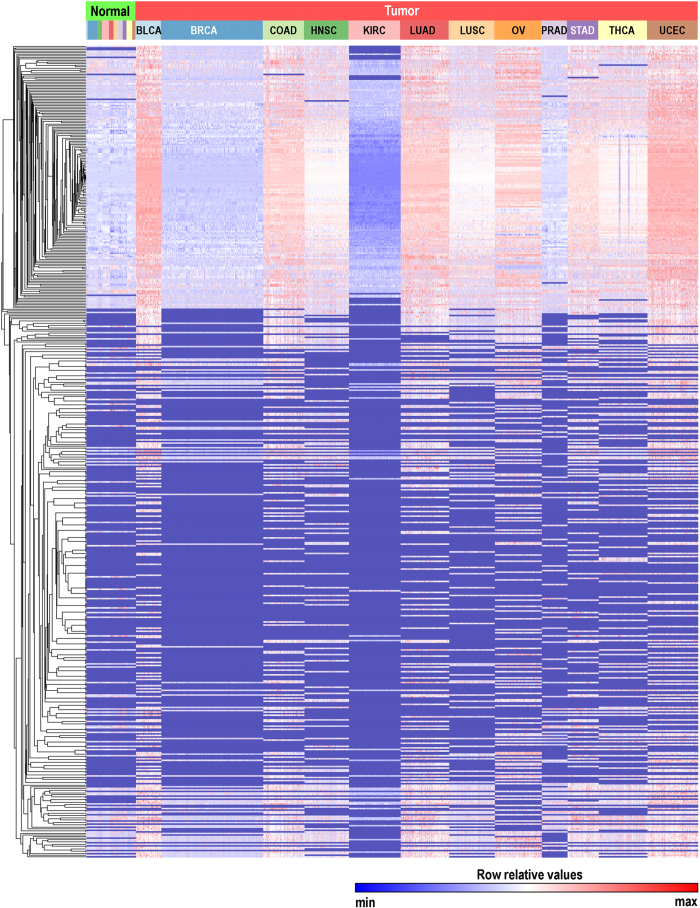
piRNA expression in non-malignant and tumour tissues. Expression patterns across all non-malignant (top row green, n = 508) and tumour tissues (top row red, n = 5,752). The second row indicates source of tissue. Each row in the heatmap denotes the rank-normalized RPKM levels of each of the piRNAs expressed across non-malignant and tumour tissues, in a row-standardized scale. PiRNA expression is clustered according to expression levels. Dark blue represents low expression levels, red denotes higher expression of a given piRNA.

Globally, we noticed that piRNA expression levels were considerably lower in non-malignant tissue compared with tumour tissue, with the exceptions of BRCA and KIRC tumours, which showed levels comparable to those observed in non-malignant tissue (Fig. 2). Of the 522 expressed piRNAs, 324 were significantly differentially expressed between non-malignant and tumour tissues (permutation test, FDR-BH p-value < 0.01, Supplementary Table 3), most of which were overexpressed in tumours compared to non-malignant tissue, with 135 piRNAs exclusively expressed in tumours (i.e. not expressed in non-malignant tissues) (Supplementary Table 3).

We also observed intriguing patterns of size distribution amongst non-malignant and tumour tissues (Supplementary Figure 1). For non-malignant tissues, ~40% of expressed piRNAs were almost equally distributed between 29 and 30 nt length. PiRNA size distribution patterns in tumours were strikingly different displaying an enrichment of piRNAs 32 nt long (36–39% of all expressed piRNAs in each tissue), followed by 28 nt (18.5%–26.1%) and 24 nt piRNAs (13.05% 17.2%) — a pattern that was remarkably conserved across all tumour types. Remarkably, over 90% of the piRNA species for the most enriched size (32 nt) were shared amongst all tumour types assessed. While speculative, this strong enrichment could indicate selection of a common tumour-promoting functional role of these piRNAs, such as altered metabolic function or involvement in other cancer hallmarks.

**Figure 3:**
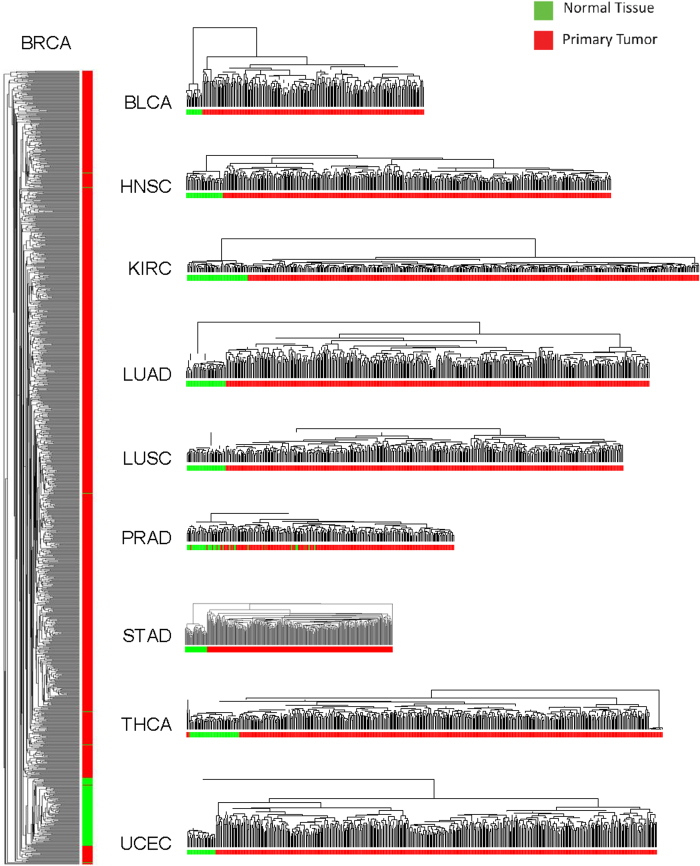
piRNA expression patterns differentiate between normal and tumour tissues. Unsupervised hierarchical clustering (average linkage, Euclidean distance) analysis on rank normalized expression values in non-malignant (NM, green) and tumour (T, red) samples derived from the following tissues: bladder (BLCA, NM = 19, T = 260), breast (BRCA, NM = 103, T = 1,043), head and neck (HNSC, NM = 43, T = 455), kidney renal clear cell (KIRC, NM = 71, T = 529), lung adenocarcinoma (LUAD, NM = 46, T = 497), lung squamous cell (LUSC, NM = 45, T = 469), prostate (PRAD, NM = 50, T = 263), stomach (STAD, NM = 38, T = 320), thyroid (THCA, NM = 59, T = 499), and uterine corpus (UCEC, NM = 33, T = 518). COAD and OV were excluded from this analysis because they had one and zero samples derived from normal tissue, respectively.

Unsupervised hierarchical clustering of piRNA expression profiles revealed a remarkably distinct segregation of tumour and non-malignant samples within individual tissue types with the exception of PRAD samples (Fig. 3 and Supplementary Figures 2–11). These data suggest that distinct piRNA expression programs are associated with tumourigenesis and support the hypothesis that piRNAs may be functionally important to cancer biology^9^,^46^.

### piRNAs exhibit pan-cancer and tumour-type specific expression patterns

**Figure 4:**
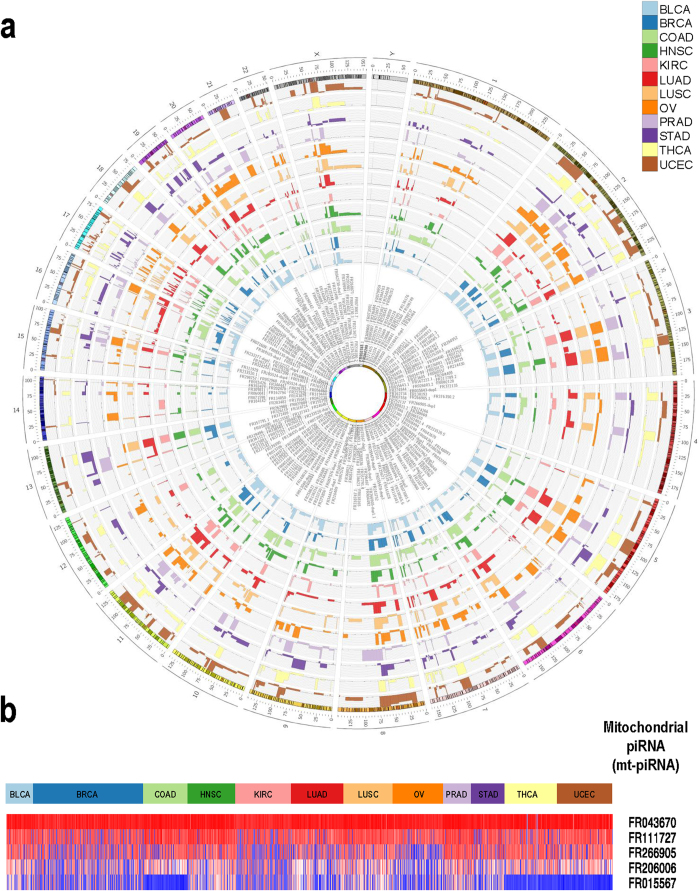
Genome-wide expression of piRNA **a**) Circular representation of genome-wide expression of piRNAs in 5,752 tumour samples derived from 12 different tissues. An ideogram of human chromosomes is shown in the outer ring, with chromosomes coloured in the same manner than the innermost ring, where the name of the expressed piRNAs are shown considering their chromosome location. Each concentric track between the two ideograms represents a histogram of average RPKM piRNA expression for each tumour type minus the expression of the same piRNA observed in non-malignant tissue (if expressed). Thus, the figure represents tumour-specific expression. The darker line on each track represents an average RPKM value = 0. Bars above this line represent that the expression of a given piRNA is higher in tumours, while bars below correspond to piRNA expressed at higher levels in non-malignant tissue compared to tumours. **b**) Detailed view of mt-piRNA expression across tumour types. Rank-normalized expression of the five most highly expressed piRNAs is shown.

We next focused our attention on piRNA expression patterns in malignant tissues. As evident in Fig. 4a, most piRNAs were commonly upregulated (relative to non-malignant tissues) across the majority of tumour types. One particularly interesting example we identified was the high level (with the exception of BRCA and KIRC) upregulation of piRNAs mapping to the mitochondrial genome (mt-piRNAs). While expression of some mt-piRNAs such as FR015567 were highly variable across tumour-types, with particularly low expression levels in COAD, THCA and UCEC, other mt-piRNAs such as FR043670, were highly expressed across almost all tumour types (Fig. 4b). The piRNAs observed here to be upregulated in a pan-cancer manner might represent a core set of genes that contribute to oncogenic properties of mt-piRNAs. Additionally, we assessed tumour piRNA expression data derived from independent cancer cohorts, including bladder (GSE31616, n = 10), breast (GSE29173 and GSE28884, n = 167), colon (GSE63119, n = 50), lung (GEO Accession numbers pending; adenocarcinoma subtype, n = 30, squamous cell carcinoma subtype, n = 30) and stomach (GSE36968, n = 25) tumours. This analysis revealed a high degree of concordance with normalized expression levels of the top 50 highest expressed piRNA for each of these tumour types (Supplementary Figure 12).

**Figure 5:**
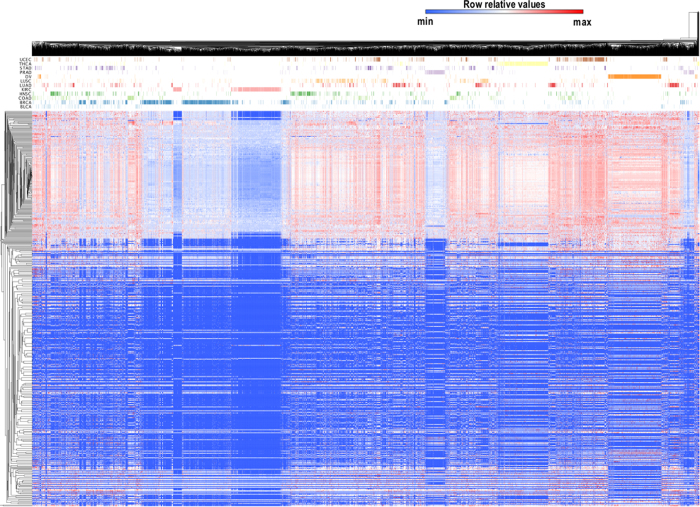
Unsupervised clustering of piRNA expression in tumour tissues. An unsupervised hierarchical clustering (average linkage, Euclidean distance) of piRNA expression profiles was performed on tumour samples derived from all analyzed tissues. Tumour types are colour-coded and separated by rows under the dendrogram. Rank-normalized expression values are expressed on an row-standardized scale and range from low (dark blue) to high (red) expression.

We next conducted unsupervised hierarchical clustering of all tumour piRNA expression profiles using pan-cancer standardized RPKM values (Fig. 5). We observed that while tumours generally clustered by tissue-of-origin, not all tumours of the same type conform to this pattern, suggesting heterogeneity exists at the level of piRNA expression within some tumour types. For example, PRAD and OV tumours, and to a lesser extent KIRC and THCA, formed discrete clusters (i.e. a low number of samples from other tumour types are observed in clusters dominated by these tumours). For KIRC tumours, distinct clustering appeared to be largely attributable to moderately expressed piRNAs, which were very highly expressed in other tumour types. Conversely, we noted that other tumour types clustered heterogeneously with tumours from other origins. For example, individual BLCA and STAD tumours were scattered among tumours of other origins, with no clusters containing a majority of these tumour samples.

**Figure 6:**
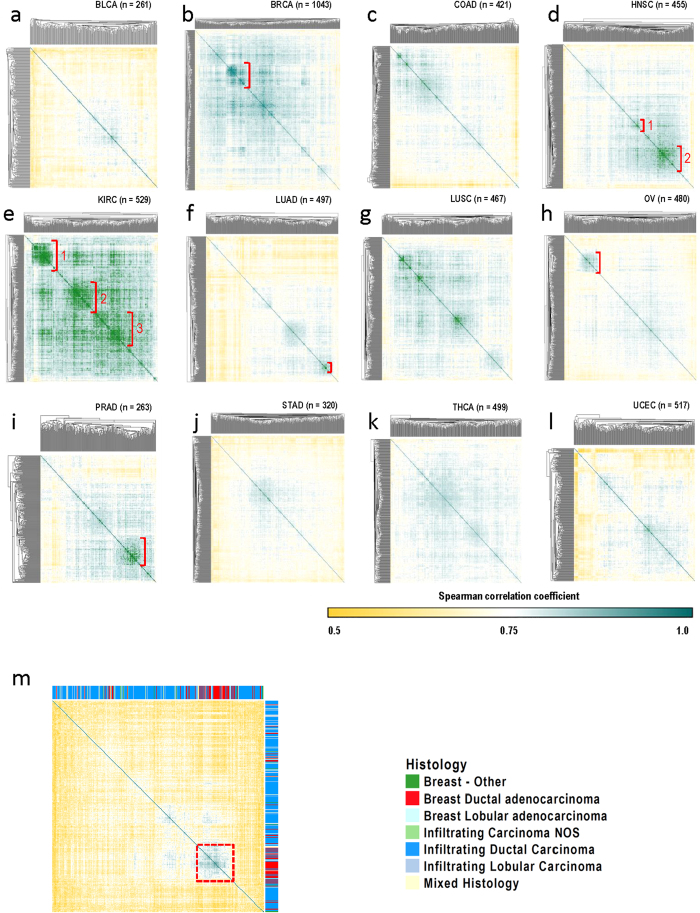
Correlation of piRNA expression patterns across samples within the same tumour type. a–l) Spearman correlation-based matrices for each tumour type. **a**) BLCA, **b**) BRCA, **c**) COAD, **d**) HNSC, **e**) KIRC, **f**) LUAD, **g**) LUSC, **h**) OV, **i**) PRAD, **j**) STAD, **k**) THCA, and **l**) UCEC. Darker green boxes indicate a higher correlation among samples, while yellow indicates a poorer correlation. Red brackets indicate clusters of highly related samples that were selected for correlative analyses with clinical variables. m) Spearman correlation-based matrix for 415 breast tumour samples with available histology information. A cluster of highly correlated samples (dashed red square) is significantly enriched (p value <0.0001, Fisher Exact Test) for breast ductal adenocarcinoma histology.

In order to further investigate how similar or divergent piRNA expression patterns were within tumours from the same tissue-of-origin, we compared similarity of samples based on their piRNA expression pattern. To that end, we built similarity matrices for each tumour type, defined by Spearman correlation coefficients (Fig. 6a–l). This analysis revealed tumour sub-group piRNA expression patterns, especially in KIRC, HNSC, PRAD, LUAD, OV, and BRCA.

### Clinically-relevant tumour subtypes exhibit different piRNA expression patterns

We were next interested in assessing whether tumours corresponding to distinct clusters in our similarity matrices for individual tumour types were enriched for clinical features relevant to the corresponding cancer type (Supplementary Table 4). For KIRC, we assessed whether metastasis, nodal status, stage and presence of *Von Hippel-Lindau* (*VHL*) mutation —a common mutation in kidney cancer— were significantly different across the three distinct tumour clusters (Fig. 6e) using multivariate analysis of variance. Intriguingly, KIRC tumour stage was significantly different across the three major sub-clusters based on both univariate (p = 0.0346) and multivariate (p = 0.03281) analyses. Cluster 1 (in Fig. 6e had the highest proportion and lowest proportion of Stage I and Stage IV tumours, respectively. We next assessed tumour location, alcohol consumption, smoking-status and pack-years, stage and grade and for HNSC tumours, and found nodal status was significantly different (multivariate p value = 0.002, univariate p value = 0.0098) between distinct clusters indicated in Fig. 6d (higher fraction of node negative tumours in Cluster 2). For PRAD, we found that Gleason score—a grading system for prostate carcinoma, where higher scores are associated with aggressive growth patterns — was often low, with the majority (72%) of prostate tumours in the PRAD cohort having a Gleason score of 7 or less. The cluster identified in the PRAD similarity matrix (Fig. 6i) has a significantly higher proportion of cases with Gleason scores ≥8 (multivariate p value = 0.00752, univariate p value = 0.0344). We did not find any significant clinical features associated with tumour sub-group clusters evident in other similarity matrices such as those for LUAD and OV (Fig. 6f,h).

Since breast cancer represented the largest tumour set in our analysis, and is a cancer type with well-defined clinical subtypes, we were able to assess whether the highly similar group of tumours indicated by the red dashed box in Fig. 6m was associated with a specific histological subtype within the 415 BRCA tumours that had available histological data. Strikingly, the cluster of highly correlated tumours was significantly enriched for breast ductal adenocarcinoma (p value < 0.0001, Fisher Exact Test) (Fig. 6m). The piRNA expression signature distinguishing this subtype is listed in Supplementary Table 4. Taken together, it is evident that piRNA expression is associated with clinically relevant features of individual tumour types.

### Prognostic value of piRNA expression

**Figure 7:**
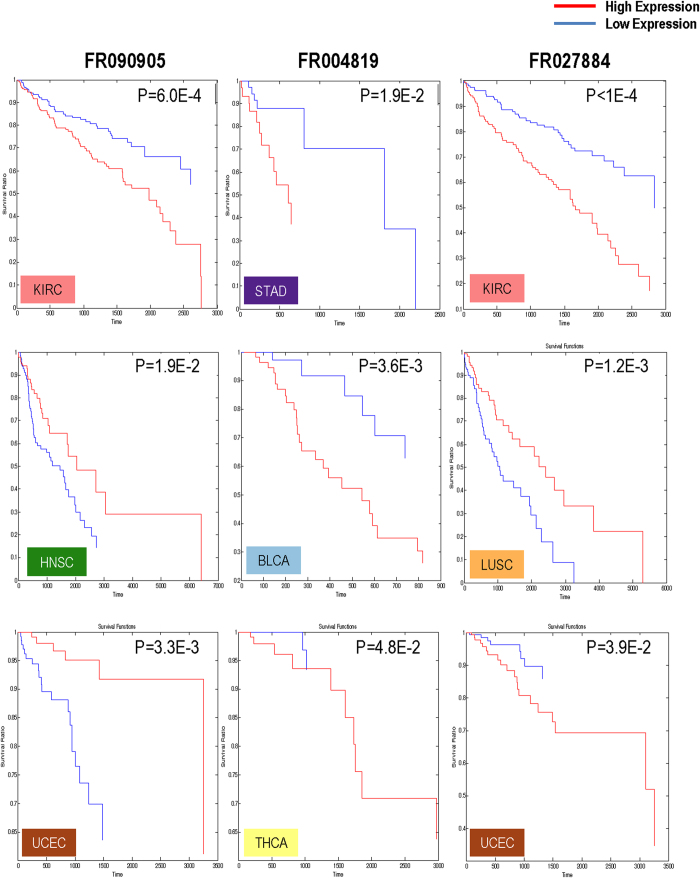
piRNA-based survival analysis. We assessed the association of piRNA expression with cancer patient survival using a logrank test. We considered the 522 piRNAs exclusively expressed in tumours in 4,976 samples where survival information was available (12 tumour types). Survival associations were only calculated for piRNAs that were detectably expressed in at least two-thirds of the samples for each particular cancer type. Patient outcome was compared in patients with piRNA expression levels ranking in the top and bottom tertiles of expression.

We next assessed the association between piRNA expression and cancer patient survival on a per cancer-type basis using a log-rank test (Fig. 7). We identified a subset of piRNAs whose expression was associated with survival in at least one tumour type (Supplementary Table 5). These piRNAs had either the same associations in each tumour type (e.g. high levels of FR004819 associated with poorer patient survival in STAD, BLCA, THCA), or had opposite associations in different tumour types. For example, high expression of FR090905 is associated with poor patient survival in KIRC, and improved survival in UCEC. Notably, FR090905 was significantly associated with survival in three different tumour types (KIRC, HNSC, and UCEC), and was nearly significant in LUSC (p value = 0.065, after false discovery rate correction for multiple testing), highlighting the potential importance of this piRNA in multiple tumour types.

Additionally, we assessed whether the prognostic significance of piRNAs we identified in breast cancer (the largest cancer cohort analyzed) could be replicated in an additional, although smaller cohort of 97 breast tumour samples with clinical follow-up information (GEO accession: GSE29173). Indeed, two piRNAs associated with survival in the TCGA breast cancer cohort (FR378984 and FR025321) validated in this external cohort (Supplementary Table 5).

### Genomic localization of somatically expressed piRNAs

In contrast to the piRNAs previously described as expressed in germline cells, which predominantly map to piRNA clusters, we found that only 10.8% and 14.3% of the piRNAs expressed in normal somatic and tumour tissues mapped to known human piRNA clusters, respectively. However, our findings were in line with a recent somatic piRNA expression study that described only 14% of piRNAs expressed coordinately with liver regeneration stages mapped to known piRNA genomic clusters^34^. We also noted that 70.4% of expressed piRNAs mapped to known protein-coding and non-coding transcripts, with 209 piRNAs mapping to introns of known genes. Additionally, 11.8% of expressed piRNAs mapped within 100 base pairs up- or downstream of repetitive elements of which 53% were also situated within coding and non-coding transcripts. Of the repetitive element-associated piRNAs, 69.3% corresponded to short interspersed nuclear elements (SINES) and long interspersed nuclear elements (LINES) (Supplementary Table 4). Interestingly, the sequences of 17 piRNAs expressed in tumours overlapped with gene mutations described in the Catalogue of Somatic Mutations in Cancer (COSMIC) (Supplementary Table 6).

### A public piRNA expression atlas to support future piRNA studies

To facilitate visualization, cross-tissue comparisons, and integration with other genomic features, we have made this information available through a custom UCSC genome browser track displaying normalized mean RPKM piRNA expression levels for these 6,260 samples summarized for each malignant or non-malignant tissue type (accessible at http://goo.gl/cnMwSv, Supplementary Figure 13). Users can also complement this information with expression matrices of RPKM values for each expressed piRNA (i.e., RPKM ≥1, n = 522) for any individual sample (Supplementary Table 2). 

## Discussion

Investigation into the role of piRNAs in the establishment of genetic, epigenetic, and transcriptional patterns —and their involvement in key processes in germline and more recently, somatic cells—is a rapidly growing field of research^8^,^34^,^39^,^40^,^47^,^48^. While expression patterns of other small non-coding RNAs (e.g. miRNAs), have been extensively described in a tissue-specific context for multiple eukaryotic organisms and almost all cancer types^49^ in humans, malignant and non-malignant piRNA expression patterns are largely uncharted. Thus, we assessed piRNA transcriptomes in over 6,000 normal and tumour tissues from 11 organ sites. Our findings revealed that, 1) piRNAs are expressed in all malignant and non-malignant human somatic tissues analyzed, 2) piRNA expression patterns are specific to malignancies and their clinical groupings, 3) some piRNAs display pan-cancer expression patterns, such as the overexpression of mitochondrial piRNAs, and 4) subgroup-specific piRNA expression patterns from tumours of the same organ exist and correlate with key clinical features relevant to each tumour type. Moreover, we found a subset of cancer-specific piRNAs associated with patient survival either across multiple malignancies or specifically in one tumour type.

Relative to the large number of piRNAs encoded in the genome, only a small proportion of the piRNAs (1.3%) were expressed in non-malignant human somatic tissues. While the number of total miRNA species expressed at any level in somatic tissues is much higher compared to piRNAs, the expression patterns of piRNAs in somatic tissue resembles those of miRNAs, where a small number of unique species represent most of the total expression^49^. It is also possible that, as with other non-coding molecules (e.g., long non-coding RNAs), low expression levels may be sufficient to exert profound biological effects^50^. While very lowly expressed piRNAs (i.e., ≤1 RPKM) were filtered from our analysis, the biological importance of these molecules may be elucidated by further interrogation using deep sequencing methods and functional assessment in future studies.

The number of piRNAs expressed in somatic tissue is considerably lower than observed in germline tissues. This is expected, since piRNAs and PIWI proteins play critical roles in germline development and gametogenesis, including germline determination, stem cell maintenance, meiosis, spermatogenesis, and transposon silencing^51^. In the original piRNA discovery paper, Girard *et al.* sequenced candidate piRNA populations from human testes, obtaining 52,099 sequences^7^. Another more recent study that analyzed human testes of 3 individuals obtained 14,608,234 reads, of which 20,121 mapped to piRNAs. The top 10 abundant piRNAs represent 61% of total piRNAs, reinforcing that a large fraction of expressed piRNAs are expressed at low levels^52^.

Most piRNAs are derived from long, single-stranded RNA precursors transcribed from specific genomic loci known as “piRNA clusters”^11^,^15^,^16^,^17^,^18^. In contrast to this pattern of expression, we observed that only 10.8% of the detected piRNAs expressed in non-malignant somatic tissues mapped to known human piRNA clusters. Most expressed piRNAs (70.4%) mapped to known human transcripts, while 11.8% were located within 100 base pairs of repetitive sequences (half of these located within coding and non-coding transcripts). This pattern has been previously observed^7^, and raises the possibility that transcripts hosting piRNAs, particularly those containing SINE or LINE repeats, might either regulate or be regulated by piRNA expression in human tissues.

Overall, clustering of non-malignant tissues based on piRNA expression revealed a high concordance in piRNA expression levels, with most piRNAs being either highly or lowly expressed across all tissues. However, some tissues, such as prostate and thyroid, displayed surprisingly tissue-type specific piRNA expression patterns (Fig. 1). Other tissues, such as stomach and bladder, did not form discrete clusters. These findings highlight a tissue-specific expression pattern, potentially reflecting tissue-specific functions, similar to that found for other small non-coding RNAs^8^. These patterns also highlight the importance of defining baseline tissue-specific expression levels for the interpretation of piRNA expression in tumours. It is noted that, since all non-malignant tissues were derived from patients with cancer, it is possible that tissue-specific patterns observed in this study may deviate to some degree from that of true normal.

Although tumours expressed twice as many piRNAs as non-malignant tissues, the overall number expressed similarly represented a small fraction of the assessed piRNAs (3.05% out of 20,831). This is consistent with what has been previously described for small cohorts of tumours and some cancer cell lines^41^,^43^,^44^,^45^. Also, similar to that observed in non-malignant tissues, a low percentage (14.3%) of piRNAs expressed in tumour tissues mapped to known human piRNA clusters. Since the majority of the expressed piRNA loci mapped outside of known piRNA genomic clusters in both malignant and non-malignant tissues, piRNA clusters may be predominantly silenced in somatic tissues.

Remarkably, with the exception of prostate adenocarcinoma, tumour-specific piRNA expression patterns appeared distinct from non-malignant tissues (Figs. 2 and 3). The imperfect tumour-normal clustering patterns observed for prostate tumours might be explained, at least in part, by the vast proportion of tumours with low Gleason scores. Interestingly, one of the stark differences found between tumours and non-malignant tissues was the high expression of piRNAs mapping to mitochondrial DNA in tumour tissues (Fig. 4), which is in line with the fact that mt-piRNA precursors exhibit high expression levels in cancer cell types and tumours^53^. However, we cannot rule out the possibility that increased expression of mt-piRNAs may be related to increased mitochondrial copy number in tumours^54^. Altered cellular metabolism in proliferating cells is a well documented phenomenon in cancer, thus it would be interesting to explore the potential metabolic role of these piRNAs in tumour cells^55^,^56^,^57^,^58^.

Although some commonalities in piRNA expression were evident across all cancer types, piRNA expression levels were capable of clustering samples by tumour-type for some tissues (Fig. 5). Tissue-specific piRNA clustering was especially evident for PRAD, OV, KIRC, and THCA, whereas other tumour types were more heterogeneous, such as BLCA and STAD. A deeper investigation of similarities in piRNA expression within individual tissues, intriguingly revealed intra-tumour piRNA sub-groups. Sub-groups within KIRC, HNSC, PRAD, and BRCA (Fig. 6) were associated with key clinical features relevant to these cancer types, including tumour stage, nodal status, Gleason score, and histological subtype, respectively. Moreover, we discovered that a subset of piRNAs were significantly associated with survival in at least one tumour type (Fig. 7; Supplemental Table 4). This indicates that piRNAs could represent potential prognostic markers similar to miRNAs, and should be evaluated on a tissue-specific basis in future studies.

Collectively, our analysis of the piRNA transcriptome provides: 1) evidence supporting a potentially tissue-specific role for piRNAs in both tumour and non-malignant somatic tissues that may be clinically relevant, 2) a curated list of piRNAs for prioritization of future piRNA functional interrogation, and 3) a human atlas of 6,260 piRNA transcriptome profiles summarized according to tumour and non-malignant tissue types. As interest in this field grows and sequence detection capacities and technologies improve, new piRNA species and reference resources are sure to rapidly expand. Thus our study represents a first-generation piRNA somatic tumour and non-malignant tissue resource based on expression of piRNAs known to date, and may serve as a useful foundation for comparative studies in future works. Further mechanistic exploration of piRNAs and associated proteins will surely yield important insight into the specificity and establishment of epigenetic patterns, transcriptional regulation, and functional involvement in normal development and human disease. We acknowledge that a major caveat to our study is that it is correlative; however, we hope that our findings may be applied to the prioritization of interesting piRNAs to commence important functional work.

## Methods

### TCGA data acquisition and small-RNA sequencing library processing

A total of 6,260 samples (508 non-malignant and 5,752 tumour) processed by The Cancer Genome Atlas (TCGA) Research Network (http://cancergenome.nih.gov/) were selected for this study. Samples spanned 10 non-malignant tissues and 12 tumour types (lung tumours are divided into LUAD and LUSC, and OV cancer lacked normal samples). The criteria for tissue inclusion were: i) availability for public use as April 2014, ii) carcinoma histology, and iii) at least 100 tumour samples. Small RNA sequencing libraries were generated at Canada’s Michael Smith Genome Sciences Centre and sequenced using the Illumina Genome Analyzer and HiSeq2000 platforms. Reads were subjected to quality control in order to exclude non-biological reads (trimming of 3′ non-biological adapter sequences) before alignment. A full description of a per-tissue processing can be found in the Synapse archive (www.synapse.org) within the following accessions: syn1461149 (BLCA), syn1461151 (BRCA), syn1461155 (COAD), syn1461156 (HNSC), syn1461159 (KIRC), syn1461166 (LUAD), syn1461168 (LUSC), syn1461171 (OV), syn1461173 (PRAD), syn1461177 (STAD), syn1461178 (THCA), syn1461180 (UCEC). Each library was sequenced to an average depth of 7.42 (range: 0.63–42.10) million reads for tumors and 10.62 (range: 1.42– 49.05) for non-malignant tissue. This sequencing strategy has been shown to be generally sufficient to identify moderate-to-low-abundance small RNAs including those exhibiting modest expression differences between samples^59^. We have also calculated the sequencing depth on a per-tissue basis in the revised manuscript (Table 2). Filtered reads were aligned to human genome (GRCh37/hg19) using the Burrows-Wheeler Aligner (BWA) algorithm^60^. Sequences aligned to the human genome (.bam files) were downloaded from the Cancer Genomics Hub (www.cghub.ucsc.edu/), after receiving authorization from the data access committee (dbgap Project ID: 6208).

### Pre-processing, quantification and selection of piRNAs

In order to select for reads corresponding to piRNAs, we extracted raw, unaligned read sequences and quality scores (FASTQ format) from the BAM file downloaded from CGhub using the “SamToFastq” tool in the Picard analysis package (http://picard.sourceforge.net). Resulting FASTQ files were trimmed based on quality (Phred quality score ≥20) and size (read length ≥21 bp), in order to enrich for high quality reads corresponding to piRNAs. Samples were realigned to human genome (hg19) using the the Spliced Transcripts Alignment to a Reference (STAR) software, with One mismatch was allowed during re-mapping of sequence reads to the human genome^61^ and a custom piRNA reference transcriptome using genomic coordinates for piRNA sequences obtained from the functional RNA database (v3.4)^62^,^63^. This reference transcriptome considers widely accepted piRNA sequence features, such as sequence bias for Uracil in the first position and presence of PIWI binding domains^7^,^62^, although known biases for 2-o-methylation in the 3′ end were not considered. miRNA annotation references, miRBase mature microRNAs version 20, and miRBase precursor microRNAs version 20, had minimal overlap with regions covered by the piRNA transcriptome reference (i.e. only 0.5% and 0.31% overlap with bases included in the piRNA transcriptome reference, respectively). The locations of piRNA clusters were obtained from piRNABank^64^.

PiRNA expression quantification was performed using PartekFlow™ (Partek Inc., MO, USA). Reads were assigned to piRNA genomic loci based on the Expectation/Maximization (E/M) algorithm^65^. Partek Genome Suite (PGS) was used to further process and filter quantified files. Statistical features for raw reads are shown in Supplementary Table 7. Reads per kilobase of exon model per million mapped reads (RPKM) was used to scale read count^66^. Following a similar criterion to that used by GENCODE database for non-coding RNAseq-based analysis^50^, we chose a conservative threshold of RPKM ≥1 for considering a piRNA as “expressed”. Additionally, we applied a frequency filter to define piRNAs as “expressed”, selecting only piRNAs expressed in at least 10% of tumour samples from the same tissue of origin.

### Visualization

Expression matrices were created using the integrative genome viewer software (IGV)^67^,^68^ and GENE-E software (Available at http://www.broadinstitute.org/cancer/software/GENE-E/). Circular representation of genome-wide expression in non-malignant stomach tissue and GA tumours was performed using the CircosPlot^69^.

### Statistical analysis

In order to perform comparisons between expressions levels, we applied the rank-normalization algorithm implemented in GenePattern, where each RPKM expression value was replaced by its rank within each individual sample. New rank-based values range from 1 (lowest RPKM expression value) to N (highest expression RPKM value, where N is the number of piRNA expressed in a given sample). If two or more piRNAs have the same expression value, their values are replaced by the average of their ranked positions. Unsupervised hierarchical clustering was performed using GENE-E software. Differential expression analyses were performed using the “Comparative Marker Selection” module implemented in Gene Pattern^70^,^71^. Briefly, this procedure determines the piRNAs that are most closely correlated with either a phenotype, or the significance of that correlation. Differential expression between the non-malignant and tumour samples was assessed through a signal-to-noise (SNR) test, and the significance (nominal P-value) was estimated using a permutation test (10,000 permutations). Nominal P-values were corrected using the Benjamini and Hochberg procedure False Discovery Rate (FDR-BH)^72^. The fold change parameter was calculated by dividing the mean expression value of tumours by the mean expression value in non-malignant tissues. Spearman correlation values of 0.75 or higher were used to call clusters in Fig. 6 heatmaps.

### Survival analysis

Survival information available for 3,433 cases (Table 1) was pulled from The Cancer Genome Atlas – Data Portal. Information concerning “days to death”, “days to last follow-up” and “vital status” was used for calculations. We assessed whether expression of piRNAs was associated with cancer patient survival using a log-rank test in MATLAB (MATLAB R2010a, The MathWorks Inc., Natick, MA, 2000), considering the 522 piRNAs expressed in tumours. Outcome was compared in patients with piRNA expression levels ranking in the top and bottom tertiles. Log-rank survival analysis was performed on piRNAs that were detectably expressed in at least two-thirds of the samples for each particular cancer type. p-values <0.05 were considered significant. Kaplan-Meier curves were used to compare survival distribution across patients with high or low expression of a given piRNA.

## Additional Information

**How to cite this article**: Martinez, V. D. *et al.* Unique somatic and malignant expression patterns implicate PIWI-interacting RNAs in cancer-type specific biology. *Sci. Rep.* **5**, 10423; doi: 10.1038/srep10423 (2015).

## Supplementary Material

Supplementary Information Supplementary Figures

Supplementary Table 1

Supplementary Table 2

Supplementary Table 3

## Figures and Tables

**Table 1:** TCGA cases and tissue types studied.

**Tissue of origin**	**TCGA code (for tumour types)**	**Number of normal samples**	**Number of tumour samples**	**Number of cases with survival information**
Bladder	BLCA	19	261	189
Breast	BRCA	103	1043	909
Colon	COAD	1	421	323
Head and neck	HNSC	43	455	391
Kidney	KIRC	71	529	484
Lung	LUAD	46	497	422
	LUSC	45	467	359
Ovarian	OV	0	480	474
Prostate	PRAD	50	263	204
Stomach	STAD	38	320	265
Thyroid	THCA	59	499	485
Uterine Corpus	UCEC	33	517	471
**TOTAL**	**12**	**508**	**5,752**	**4,976**

**Table 2:** Sequencing depth per tissue (in millions of reads).

	**TUMOURS**		**NON-MALIGNANT**	
**TISSUE**	**Average number (million reads)**	**Range (million reads)**	**Average number (million reads)**	**Range (million reads)**
Bladder (BLCA)	9.53	2.31–37.16	22.04	3.3–49.05
Breast (BRCA)	5.62	0.96–33.52	5.45	1.42–25.56
Colon (COAD)	6.95	0.63–42.1	6.07*	6.07–6.07
Head and neck (HNSC)	8.16	1.47–30.59	10.56	3.86–19.19
Kidney (KIRC)	4.6	1.24–25.67	4.76	1.84–11.59
Lung (LUAD)	8.85	0.94–26.36	10.19	2.4–19.19
Lung (LUSC)	6.29	1.31–17.88	13.17	5.35–23.33
Ovarian (OV)	7.94	1.37–25.32	N/A	N/A
Prostate (PRAD)	10.13	2.53–36.41	11.54	4.72–31.07
Stomach (STAD)	8.32	1.28–34.98	12.81	4.13–39.2
Thyroid (THCA)	9.39	2.09–34.4	12.31	4.02–21.17
Uterine corpus (UCEC)	7.77	1.07–36.88	23.2	6.93–40.41

^*^Only 1 library was available for this tissue
