# Supplementary material for: Unique somatic and malignant expression patterns implicate PIWI-interacting RNAs in cancer-type specific biology
Source: Sci Rep. 2015 May 27;5:10423. doi: 10.1038/srep10423 (PMC4444957; doi:10.1038/srep10423)
Supplement: Supplementary Information — Supplementary Figures [file srep10423-s1.pdf]

# Unique somatic and malignant expression patterns implicate PIWI-interacting RNAs in cancer-type specific biology

Victor D. Martinez[1*], Emily A. Vucic[1,3] , Kelsie L. Thu[1,3] , Roland Hubaux[1], Katey S.S. Enfield[1], Larissa A. Pikor[1], Daiana D. Becker-Santos[1], Carolyn J. Brown[1,2], Stephen Lam[1], Wan L. Lam[1]

# Supplementary Figures

**Supplementary Figure 1: Size distribution of expressed piRNA in different tissues**

A

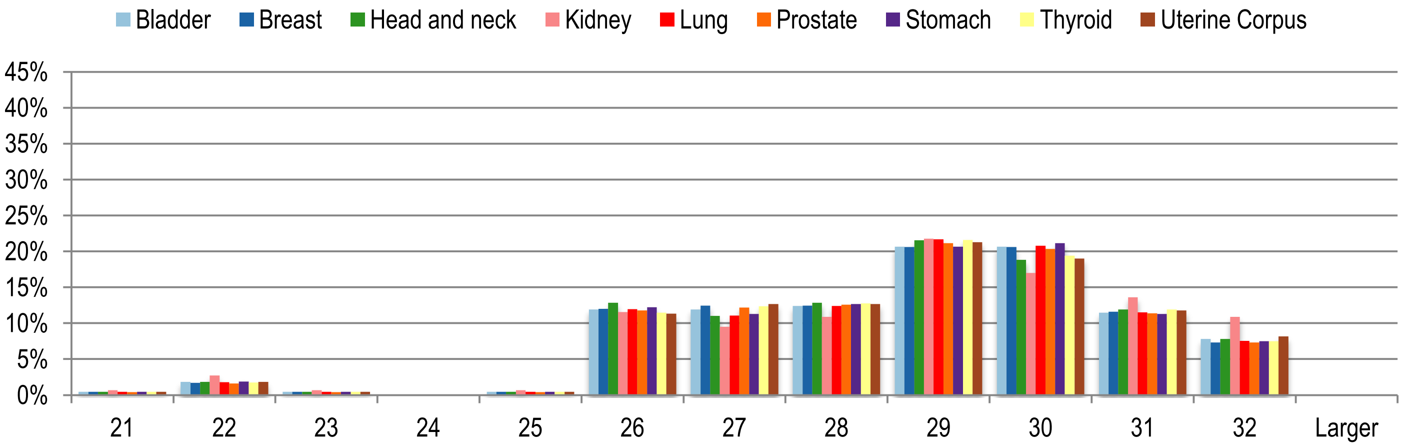


B

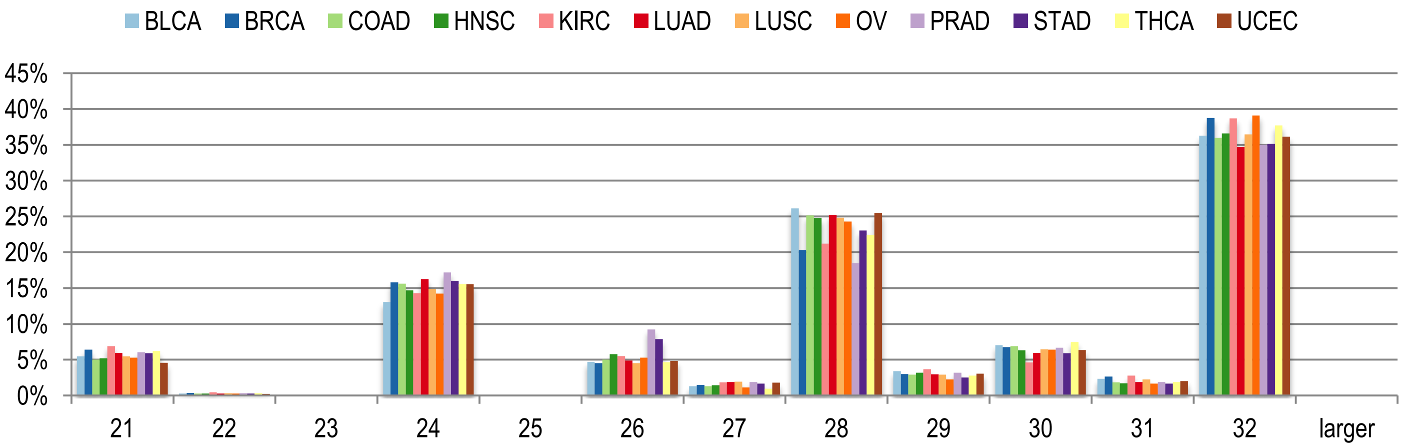


The percentages of piRNAs for each corresponding size (21-32 nt) was determined in each tissue type for: A) non-malignant and B) tumors.

**Supplementary Figure 2: Clustering analysis of normal and tumoural samples from BLCA**

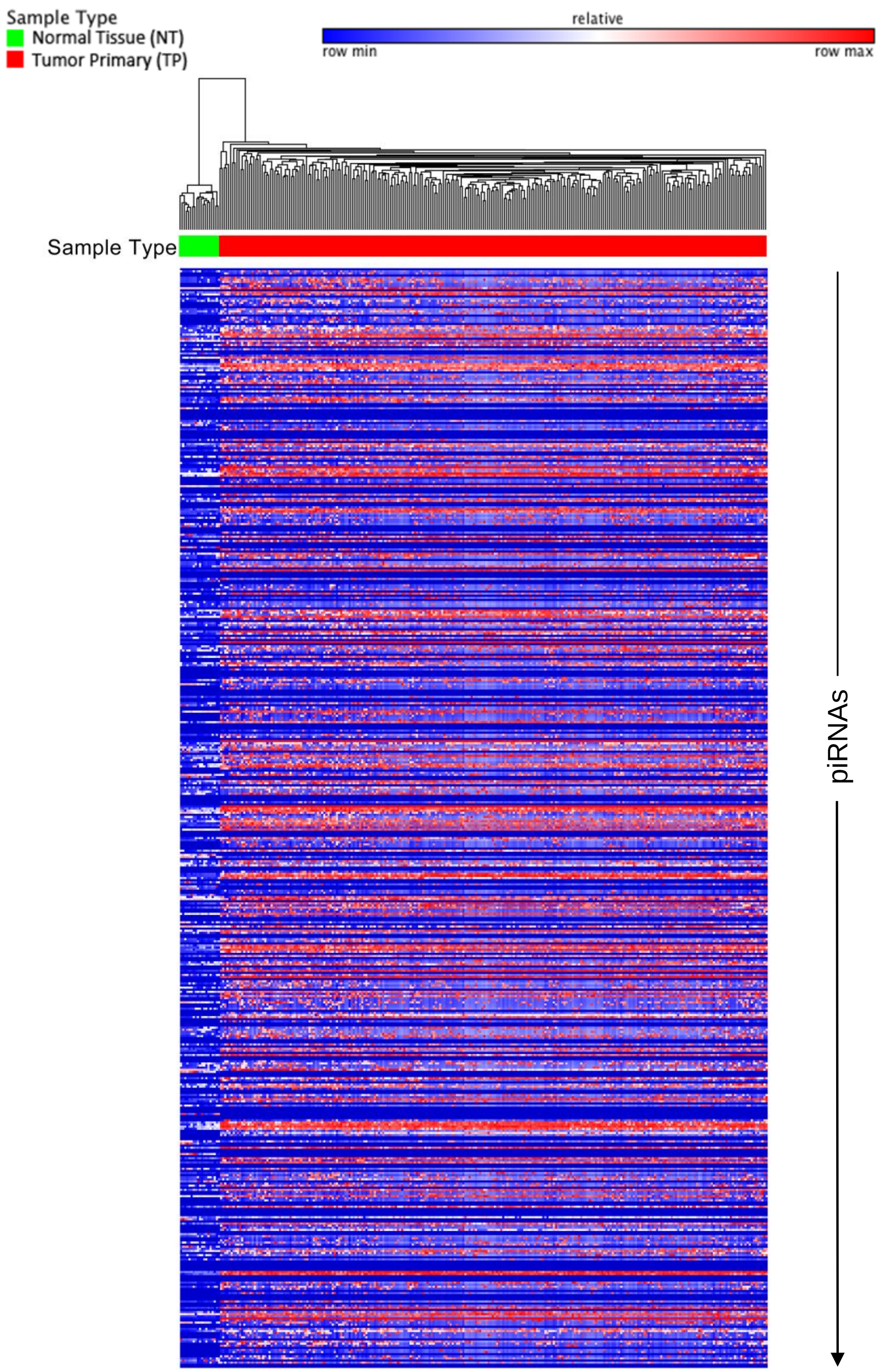


Unsupervised hierarchical clustering (Euclidean distance, average distance) of rank-normalized piRNA expression obtained from 19 samples derived from bladder non-malignant tissue and 260 BLCA tissues.

**Supplementary Figure 3: Clustering analysis of normal and tumoural samples from BRCA**

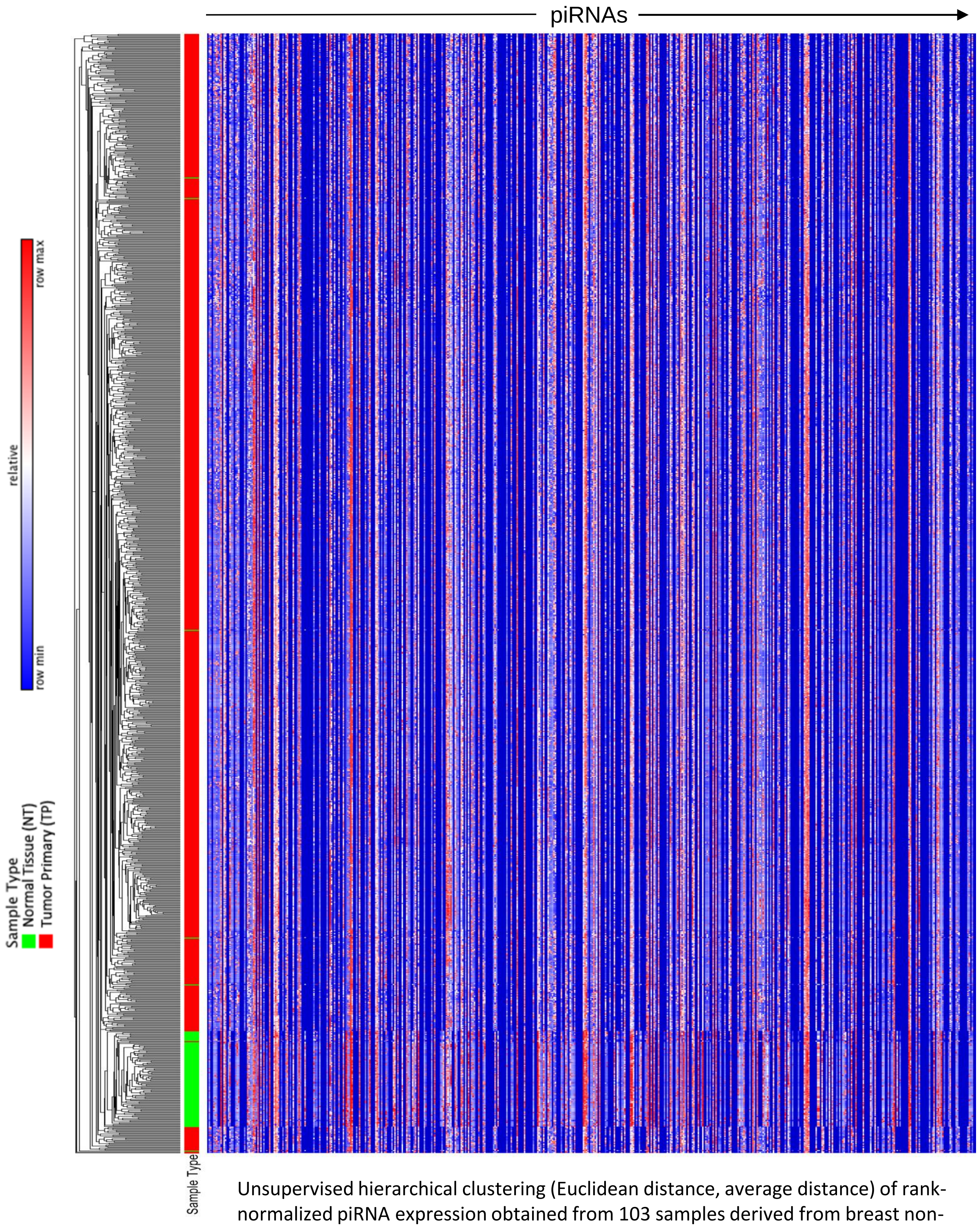


Unsupervised hierarchical clustering (Euclidean distance, average distance) of rank-normalized piRNA expression obtained from 103 samples derived from breast non-malignant tissue and 1,043 BRCA tumours.

**Supplementary Figure 4: Clustering analysis of normal and tumoural samples from HNSC**

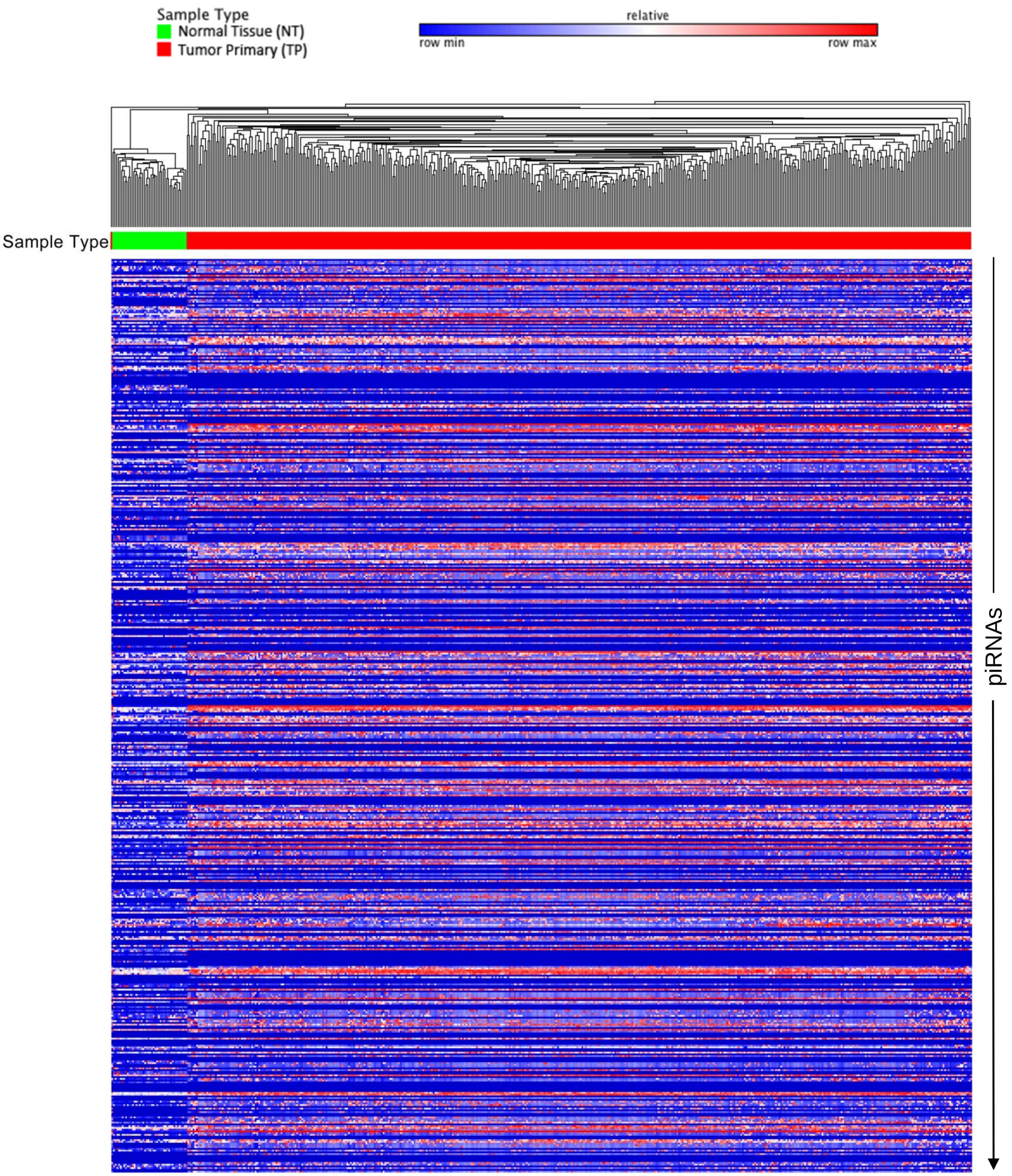


Unsupervised hierarchical clustering (Euclidean distance, average distance) of rank-normalized piRNA expression obtained from 43 samples derived from head and neck (different anatomical locations) non-malignant tissue and 455 HNSC tumours.

**Supplementary Figure 5: Clustering analysis of normal and tumoural samples from KIRC**

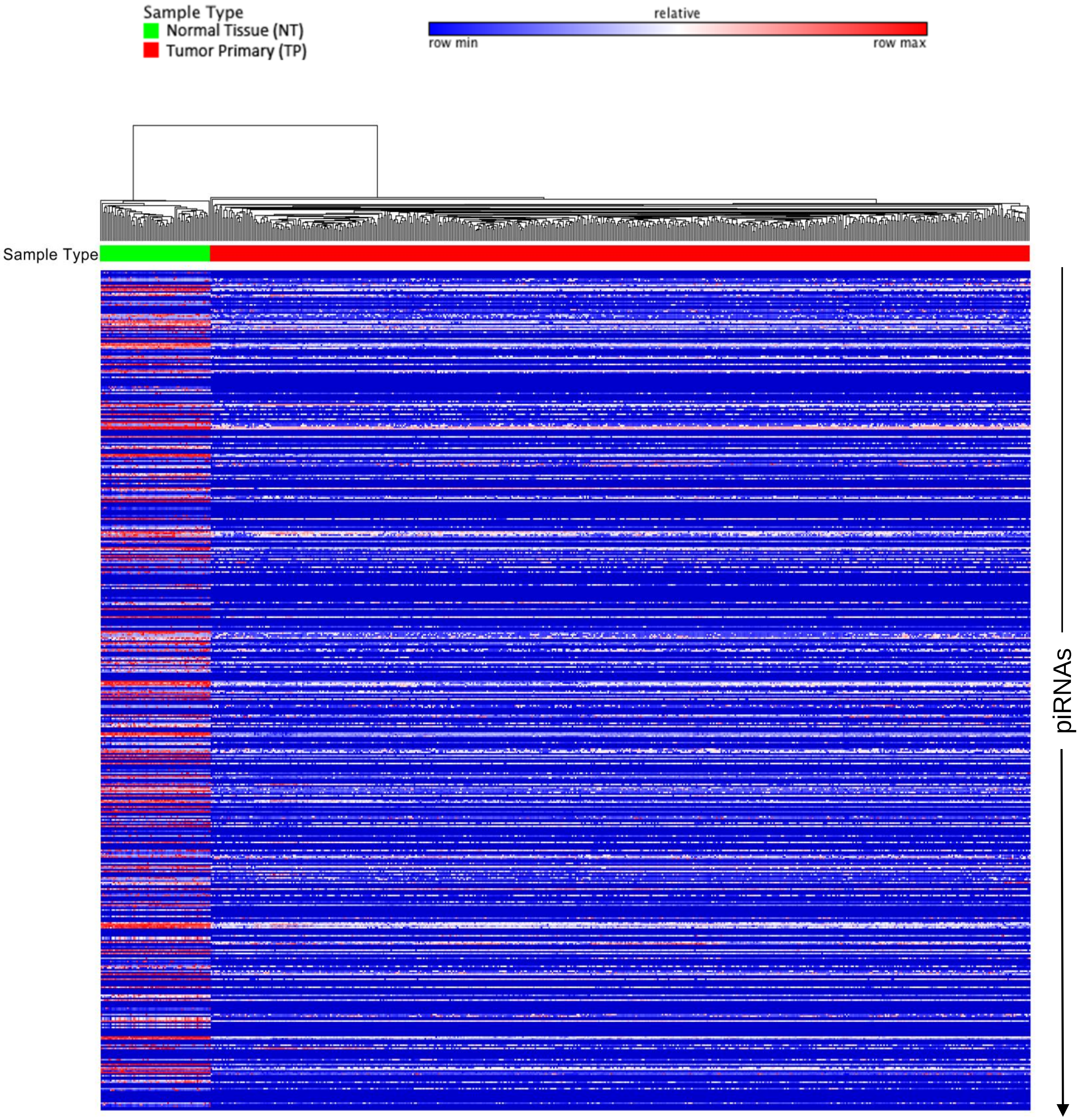


Unsupervised hierarchical clustering (Euclidean distance, average distance) of rank-normalized piRNA expression obtained from 71 samples derived from kidney non-malignant tissue and 529 KIRC tumours.

**Supplementary Figure 6: Clustering analysis of normal and tumoural samples from LUAD**

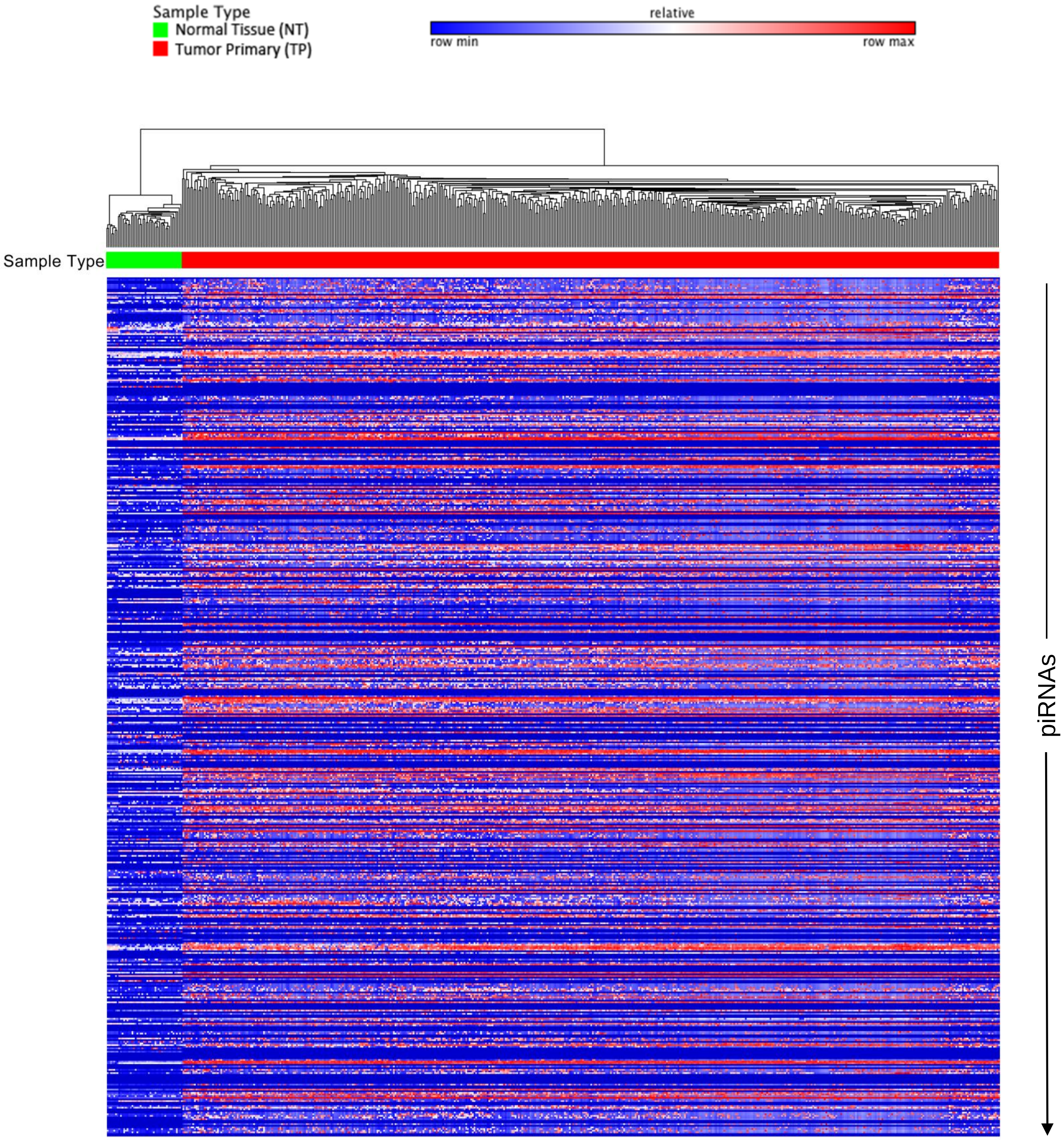


Unsupervised hierarchical clustering (Euclidean distance, average distance) of rank-normalized piRNA expression obtained from 46 samples derived from lung non-malignant tissue and 497 LUAD tumours.

**Supplementary Figure 7: Clustering analysis of normal and tumoural samples from LUSC**

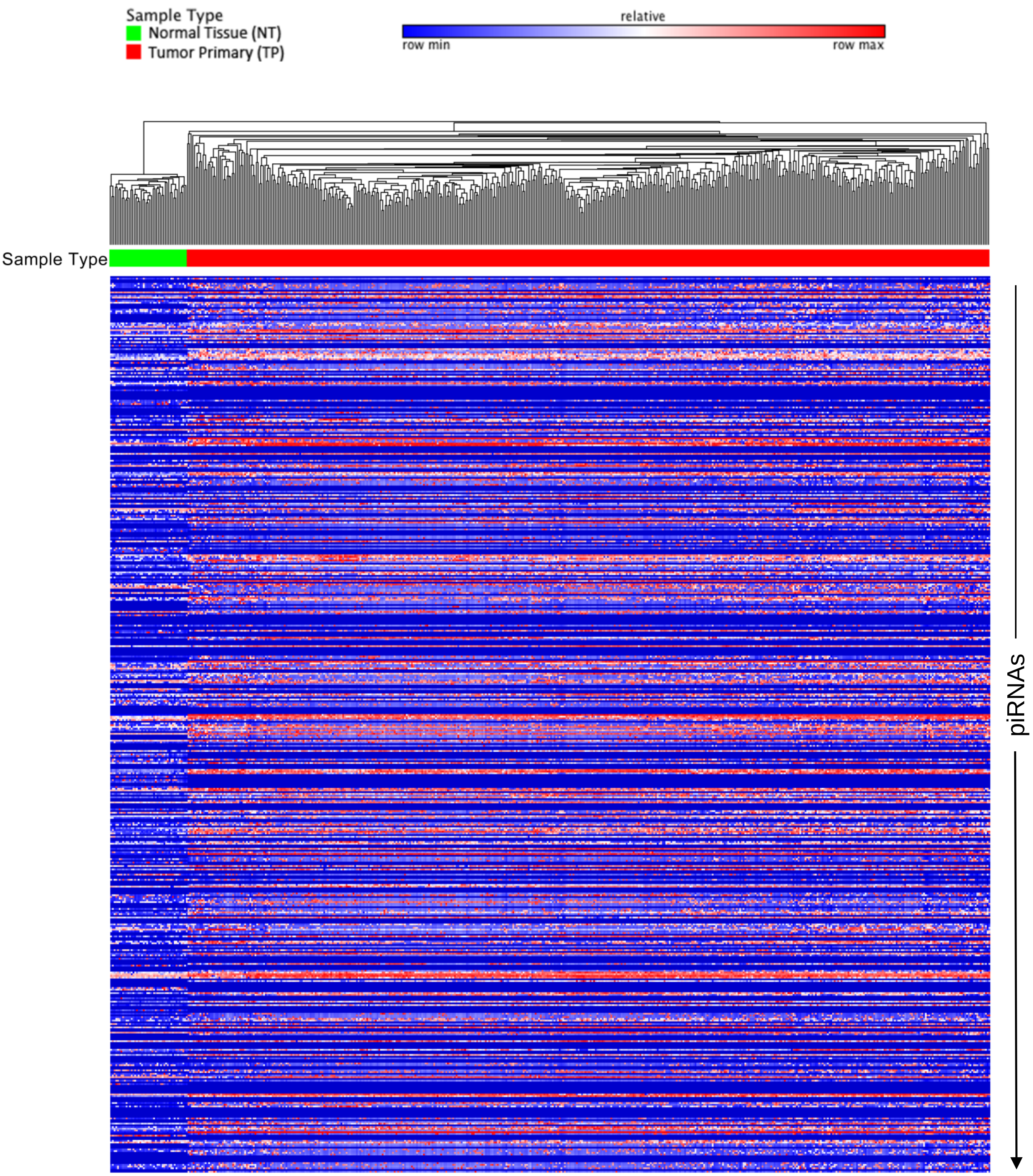


Unsupervised hierarchical clustering (Euclidean distance, average distance) of rank-normalized piRNA expression obtained from 45 samples derived from lung non-malignant tissue and 467 LUSC tumours.

**Supplementary Figure 8: Clustering analysis of normal and tumoural samples from PRAD**

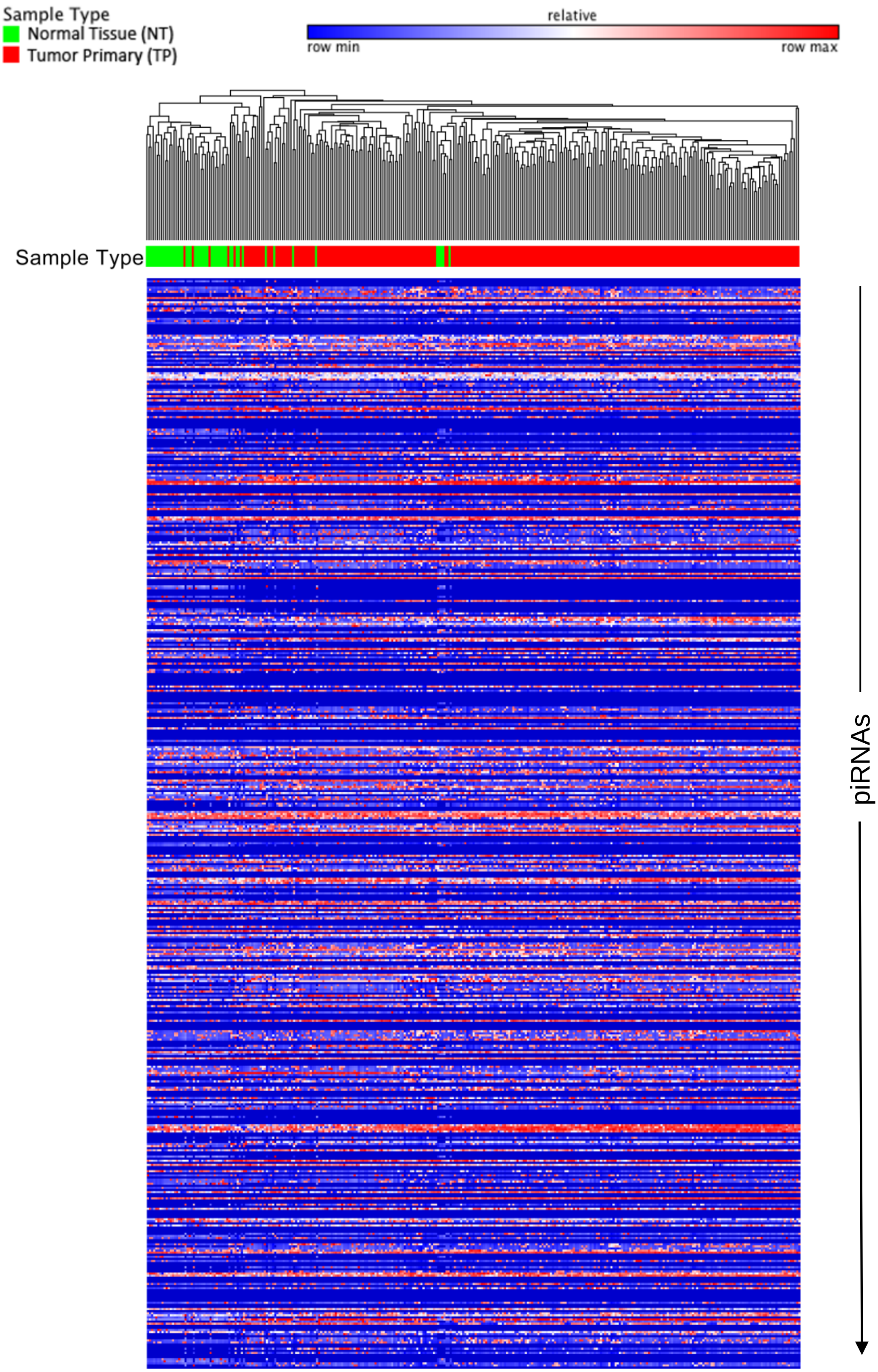


Unsupervised hierarchical clustering (Euclidean distance, average distance) of rank-normalized piRNA expression obtained from 50 samples derived from prostate non-malignant tissue and 263 PRAD tumours.

**Supplementary Figure 9: Clustering analysis of normal and tumoural samples from STAD**

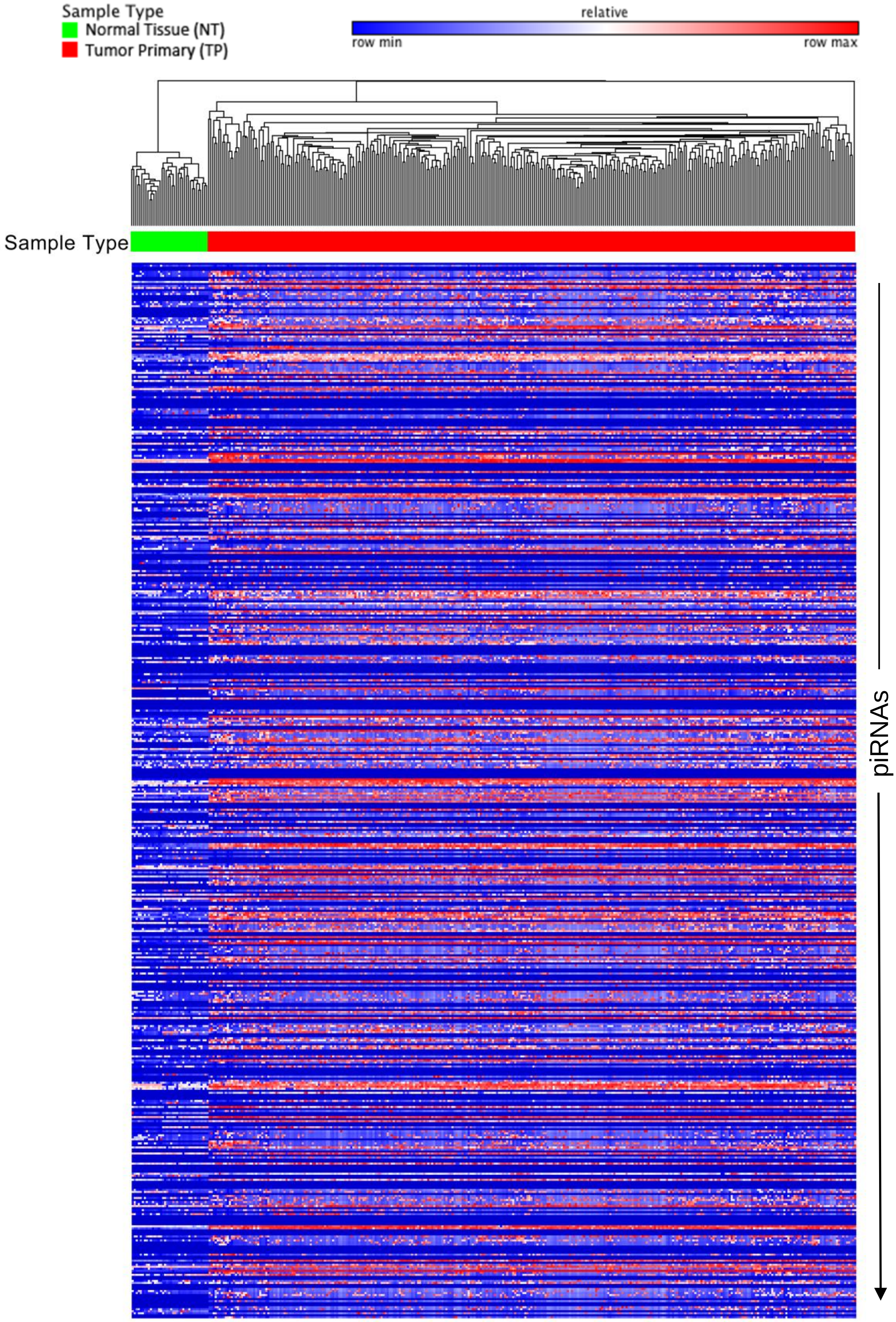


Unsupervised hierarchical clustering (Euclidean distance, average distance) of rank-normalized piRNA expression obtained from 38 samples derived from prostate non-malignant tissue and 320 STAD tumours.

**Supplementary Figure 10: Clustering analysis of normal and tumoural samples from THCA**

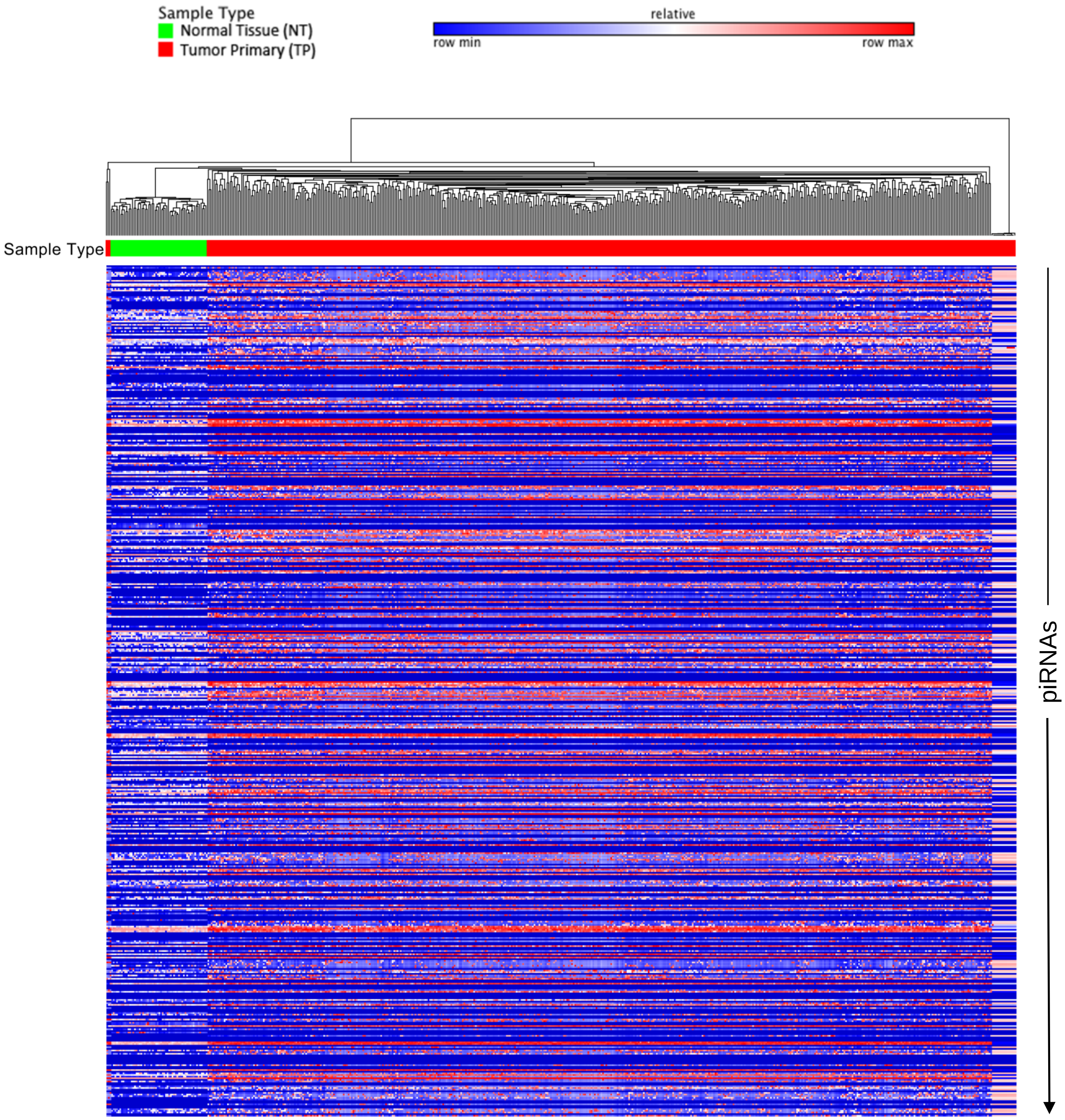


Unsupervised hierarchical clustering (Euclidean distance, average distance) of rank-normalized piRNA expression obtained from 59 samples derived from thyroid non-malignant tissue and 499 THCA tumours.

**Supplementary Figure 11: Clustering analysis of normal and tumoural samples from UCEC**

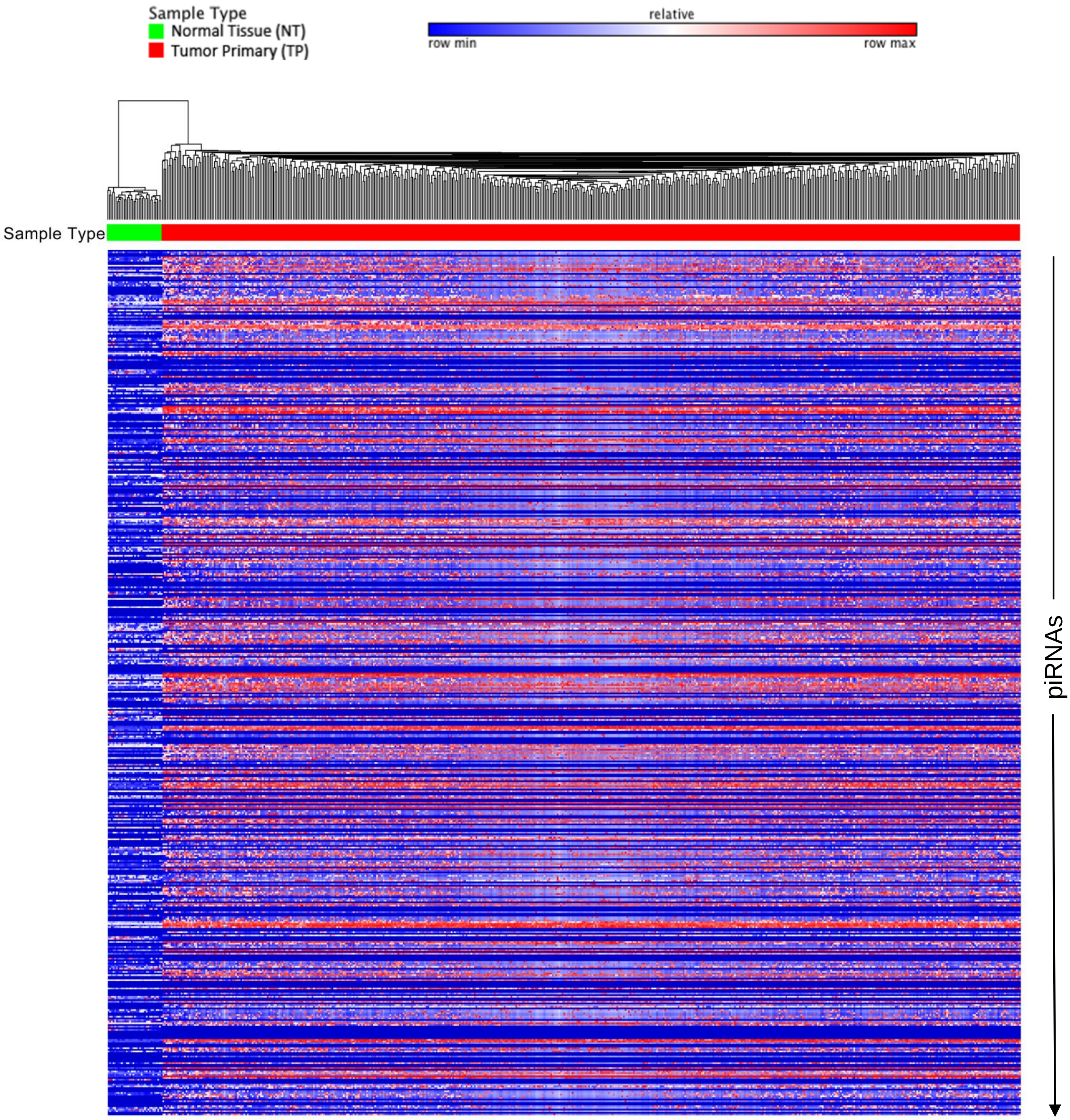


Unsupervised hierarchical clustering (Euclidean distance, average distance) of rank-normalized piRNA expression obtained from 33 samples derived from uterine non-malignant tissue and 518 UCEC tumours.

**Supplementary Figure 12: TCGA vs independent cohort validation**

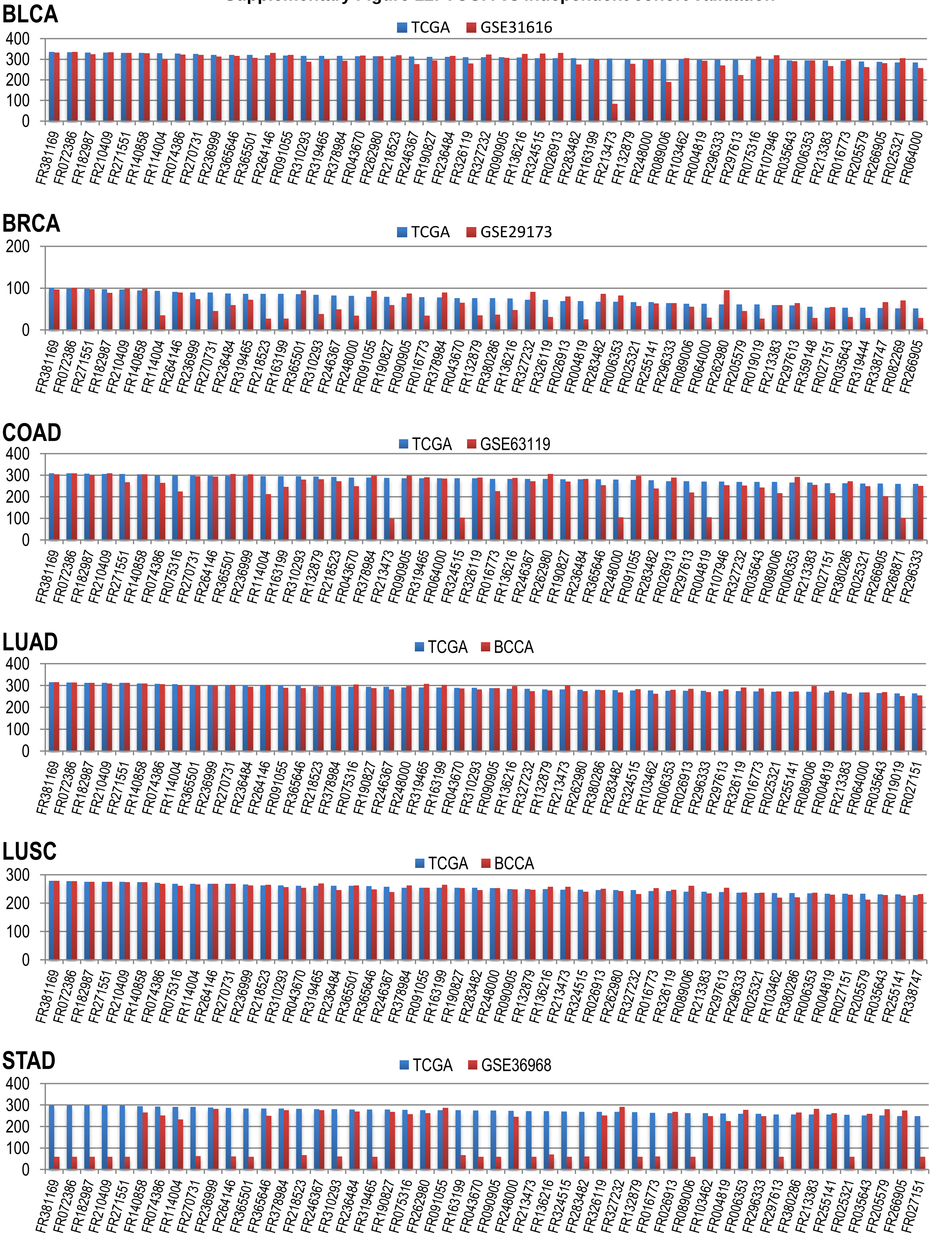

**Supplementary Figure 13: Custom UCSC piRNA expression tracks.**

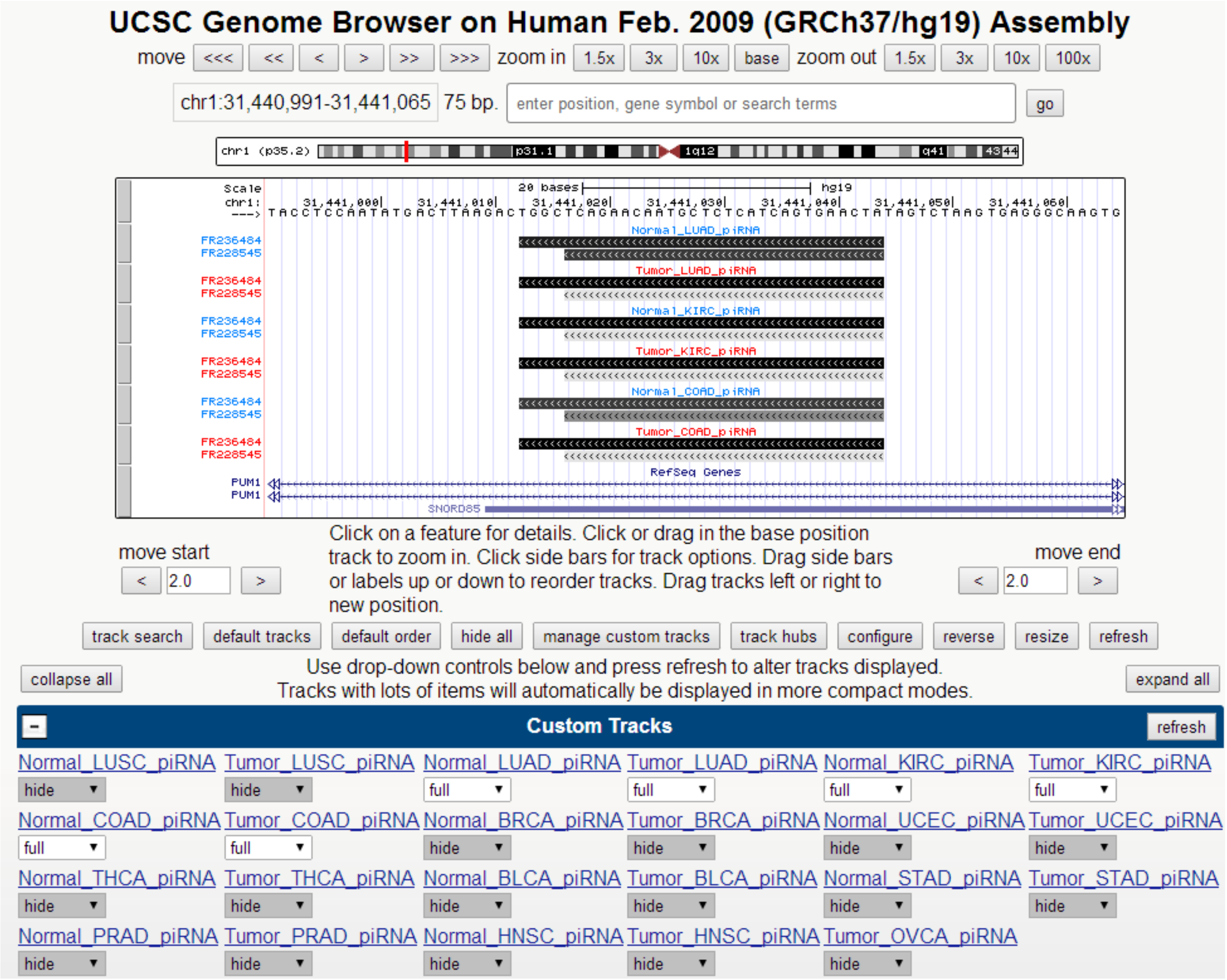


The average expression values for expressed piRNA (n=276 in normals and n=635 in tumours) were calculated and rank normalized for each normal and tumour tissue type, and used along with piRNA genomic coordinates to create custom tracks for display in the UCSC Genome Browser on the latest built (GRCh37/hg19, Feb. 2009). Expression levels are illustrated on a colour scale ranging from light gray to black indicating low and high expression values, respectively. The colour scales for each tissue type are defined by the piRNA with the lowest average expression (i.e. rank=1) and the highest average expression (rank=276 for normals and rank=635 for tumours). These publically available tracks contain rank normalized expression data for 11 normal tissue types and 12 tumour tissue types.

# Supplementary Tables

# Supplementary Table 3

| | | | Median expression (RPKM) per tumor type | | | | | | | | | |
|---|---|---|---|---|---|---|---|---|---|---|---|---|
| piRNA ID | Benjamini-H | Expression Pattern | BLCA | BRCA | HNSC | KIRC | LUAD | LUSC | PRAD | STAD | THCA | UCEC |
| FR003121 | 0 | | | | | | | | | | | |
| FR003837 | 0 | | | | | | | | | | | |
| FR004361 | 0 | Exclusively expressed in tumo | 1.2997 | 1.2357 | | 1.6724 | 0.7017 | | 0.5697 | 0.9826 | 1.0958 | 2.9612 |
| FR004819 | 0 | | | | | | | | | | | |
| FR006353 | 0 | | | | | | | | | | | |
| FR007558 | 0 | | | | | | | | | | | |
| FR008494 | 0 | Exclusively expressed in norm | 1.3124 | 0.948 | 0.3033 | 0.9875 | 1.122 | 1.5636 | 0.4201 | 1.8485 | 0.8685 | 3.2722 |
| FR008939 | 0 | Exclusively expressed in tumo | 2.3109 | 1.7251 | 0.7956 | | 1.0947 | 3.8057 | | 3.0573 | | 2.116 |
| FR013706 | 0 | Exclusively expressed in tumo | 1.0506 | | 0.1604 | 1.2546 | 0.6447 | 0.789 | | 0.9022 | 0.8755 | 2.3092 |
| FR016000 | 0 | | | | | | | | | | | |
| FR016773 | 0 | | | | | | | | | | | |
| FR018916 | 0 | | | | | | | | | | | |
| FR018977 | 0 | | | | | | | | | | | |
| FR021331 | 0 | | | | | | | | | | | |
| FR024428 | 0 | | | | | | | | | | | |
| FR025321 | 0 | | | | | | | | | | | |
| FR027884 | 0 | | | | | | | | | | | |
| FR029085 | 0 | | | | | | | | | | | |
| FR029743 | 0 | Exclusively expressed in tumo | 2.1005 | 0.9093 | 0.1802 | | 1.411 | 1.4952 | 0.7982 | 1.0179 | 2.1361 | 1.5472 |
| FR037883 | 0 | | | | | | | | | | | |
| FR048331 | 0 | | | | | | | | | | | |
| FR054474 | 0 | | | | | | | | | | | |
| FR054563 | 0 | | | | | | | | | | | |
| FR055135 | 0 | | | | | | | | | | | |
| FR061350 | 0 | | | | | | | | | | | |
| FR069557 | 0 | | | | | | | | | | | |
| FR072386 | 0 | | | | | | | | | | | |
| FR073001 | 0 | | | | | | | | | | | |
| FR075316 | 0 | | | | | | | | | | | |
| FR095339 | 0 | | | | | | | | | | | |
| FR097339 | 0 | Exclusively expressed in tumo | 1.1196 | 2.1007 | 0.167 | 1.0115 | 0.5711 | 0.8536 | 1.2566 | 0.7296 | 0.7485 | 1.3704 |
| FR098442 | 0 | | | | | | | | | | | |
| FR102402 | 0 | Exclusively expressed in tumo | 0.7243 | 0.9031 | 0.2418 | | 0.6987 | 1.0122 | 0.4588 | 1.1315 | 0.8086 | 2.2522 |
| FR102859 | 0 | | | | | | | | | | | |
| FR107946 | 0 | | | | | | | | | | | |
| FR118379 | 0 | | | | | | | | | | | |
| FR128806 | 0 | | | | | | | | | | | |
| FR131044 | 0 | | | | | | | | | | | |
| FR132358 | 0 | | | | | | | | | | | |
| FR133421 | 0 | Exclusively expressed in tumo | 1.3171 | 1.0553 | 0.4637 | | 1.6445 | 1.2741 | 0.39 | 0.9336 | 1.6386 | 0.9292 |
| FR135042 | 0 | | | | | | | | | | | |
| FR135938 | 0 | Exclusively expressed in tumo | 1.0667 | 0.9097 | 0.2022 | | 0.8389 | 1.0038 | | 0.7034 | | 1.1524 |
| FR138455 | 0 | | | | | | | | | | | |
| FR140858 | 0 | | | | | | | | | | | |

| | | | | | | | | | | | | |
|---|---|---|---|---|---|---|---|---|---|---|---|---|
| FR149623 | 0 | | | | | | | | | | | |
| FR157967 | 0 | | | | | | | | | | | |
| FR162144 | 0 | | | | | | | | | | | |
| FR162734 | 0 | | | | | | | | | | | |
| FR188833 | 0 | | | | | | | | | | | |
| FR190827 | 0 | | | | | | | | | | | |
| FR191737 | 0 | | | | | | | | | | | |
| FR197104 | 0 | | | | | | | | | | | |
| FR197889 | 0 | | | | | | | | | | | |
| FR202919 | 0 | | | | | | | | | | | |
| FR203981 | 0 | | | | | | | | | | | |
| FR205579 | 0 | | | | | | | | | | | |
| FR207412 | 0 | | | | | | | | | | | |
| FR210409 | 0 | | | | | | | | | | | |
| FR213383 | 0 | | | | | | | | | | | |
| FR218523 | 0 | | | | | | | | | | | |
| FR218973 | 0 | | | | | | | | | | | |
| FR224609 | 0 | Exclusively expressed in tumo | 0.9426 | 1.193 | 0.2374 | | 0.8412 | 0.9682 | | 0.9002 | 0.6636 | 1.0564 |
| FR228545 | 0 | | | | | | | | | | | |
| FR232277 | 0 | | | | | | | | | | | |
| FR233520 | 0 | | | | | | | | | | | |
| FR233702 | 0 | | | | | | | | | | | |
| FR233730 | 0 | | | | | | | | | | | |
| FR238825 | 0 | | | | | | | | | | | |
| FR242079 | 0 | | | | | | | | | | | |
| FR243752 | 0 | | | | | | | | | | | |
| FR246367 | 0 | | | | | | | | | | | |
| FR248000 | 0 | | | | | | | | | | | |
| FR252722 | 0 | | | | | | | | | | | |
| FR262980 | 0 | | | | | | | | | | | |
| FR263507 | 0 | | | | | | | | | | | |
| FR264146 | 0 | | | | | | | | | | | |
| FR266905 | 0 | | | | | | | | | | | |
| FR270731 | 0 | | | | | | | | | | | |
| FR271504 | 0 | | | | | | | | | | | |
| FR271551 | 0 | | | | | | | | | | | |
| FR274916 | 0 | Exclusively expressed in tumo | 1.8736 | 0.988 | 0.2154 | | 1.0523 | 0.7361 | 0.8171 | 0.7126 | | 1.1864 |
| FR277415 | 0 | Exclusively expressed in tumo | 3.6799 | 1.8366 | 0.2154 | 1.391 | 1.8961 | 2.0262 | 4.1132 | 1.1444 | 0.54 | 3.4276 |
| FR278590 | 0 | | | | | | | | | | | |
| FR279668 | 0 | | | | | | | | | | | |
| FR283482 | 0 | | | | | | | | | | | |
| FR284409 | 0 | Exclusively expressed in tumo | 1.394 | 0.9532 | | | 0.8906 | | 0.4786 | 0.8264 | 0.6318 | 1.6216 |
| FR284952 | 0 | | | | | | | | | | | |
| FR291903 | 0 | | | | | | | | | | | |
| FR292218 | 0 | | | | | | | | | | | |
| FR293713 | 0 | | | | | | | | | | | |
| FR294567 | 0 | Exclusively expressed in tumo | 1.342 | 0.9384 | 0.3978 | 0.9839 | 1.0981 | 1.4045 | | 1.1916 | 0.4962 | 1.941 |

| | | | | | | | | | | | | |
|---|---|---|---|---|---|---|---|---|---|---|---|---|
| FR296333 | 0 | | | | | | | | | | | |
| FR302680 | 0 | | | | | | | | | | | |
| FR306048 | 0 | | | | | | | | | | | |
| FR312701 | 0 | | | | | | | | | | | |
| FR315253 | 0 | | | | | | | | | | | |
| FR315324 | 0 | Exclusively expressed in tumors | | 1.2762 | | 1.4268 | 0.8414 | | | 0.8627 | 2.9076 | 1.4269 |
| FR319300 | 0 | | | | | | | | | | | |
| FR323470 | 0 | | | | | | | | | | | |
| FR324253 | 0 | | | | | | | | | | | |
| FR324541 | 0 | Exclusively expressed in tumors | | 0.4867 | | 0.5462 | | 0.4911 | | | | 0.4174 |
| FR327232 | 0 | | | | | | | | | | | |
| FR328873 | 0 | | | | | | | | | | | |
| FR338565 | 0 | | | | | | | | | | | |
| FR344796 | 0 | | | | | | | | | | | |
| FR348796 | 0 | | | | | | | | | | | |
| FR349959 | 0 | | | | | | | | | | | |
| FR352363 | 0 | Exclusively expressed in tumo | 0.8581 | 1.2651 | 0.6879 | | | 1.6456 | | | 1.046 | 2.5322 |
| FR353662 | 0 | | | | | | | | | | | |
| FR357447 | 0 | Exclusively expressed in norm | 4.9945 | 8.8665 | 1.0549 | 8.8919 | 5.9772 | 14.3143 | 1.8613 | 12.7507 | 1.0344 | 5.7415 |
| FR357773 | 0 | | | | | | | | | | | |
| FR359148 | 0 | | | | | | | | | | | |
| FR361464 | 0 | | | | | | | | | | | |
| FR365646 | 0 | | | | | | | | | | | |
| FR372135 | 0 | | | | | | | | | | | |
| FR378984 | 0 | | | | | | | | | | | |
| FR380227 | 0 | | | | | | | | | | | |
| FR380286 | 0 | | | | | | | | | | | |
| FR381169 | 0 | | | | | | | | | | | |
| FR381182 | 0 | Exclusively expressed in tumo | 1.1096 | 2.0097 | 0.2 | 1.0998 | 1.0961 | 1.4533 | 0.658 | 1.4259 | 1.0574 | 4.1158 |
| FR384068 | 0 | | | | | | | | | | | |
| FR389105 | 0 | Exclusively expressed in tumo | 1.2126 | 1.601 | 0.189 | | 0.8014 | 0.8041 | 1.3119 | | 0.4838 | 1.3257 |
| FR391445 | 0 | | | | | | | | | | | |
| FR393260 | 0 | Exclusively expressed in tumo | 0.6339 | 0.7349 | | | 0.6137 | 0.6283 | | 0.691 | 0.7798 | 0.7328 |
| FR019089 | 8.92E-16 | | | | | | | | | | | |
| FR217789 | 8.92E-16 | | | | | | | | | | | |
| FR236999 | 8.92E-16 | | | | | | | | | | | |
| FR245310 | 8.92E-16 | | | | | | | | | | | |
| FR255141 | 8.92E-16 | | | | | | | | | | | |
| FR337552 | 8.92E-16 | | | | | | | | | | | |
| FR295879 | 1.76E-15 | | | | | | | | | | | |
| FR318724 | 1.76E-15 | Exclusively expressed in tumo | 0.9162 | | 0.1736 | | 0.5799 | 1.0258 | | 0.7196 | 1.419 | 2.7828 |
| FR103462 | 3.49E-15 | | | | | | | | | | | |
| FR198390 | 6.05E-15 | Exclusively expressed in tumo | 1.0044 | | 0.1451 | | 0.7509 | 0.8143 | 0.4845 | 0.7667 | 0.8668 | 1.483 |
| FR086093 | 7.73E-15 | | | | | | | | | | | |
| FR196011 | 1.02E-14 | Exclusively expressed in tumo | 1.0643 | 1.086 | | | 0.8393 | 0.9848 | | 1.0588 | | 1.2732 |
| FR237180 | 2.71E-14 | | | | | | | | | | | |
| FR372317 | 3.28E-14 | Exclusively expressed in tumo | 1.1034 | | 0.2571 | | 1.1366 | 1.1985 | | 1.1889 | 0.9442 | 2.8737 |

| | | | | | | | | | | | | |
|---|---|---|---|---|---|---|---|---|---|---|---|---|
| FR177847 | 3.84E-14 | Exclusively expressed in tumo | 0.4116 | | | | 0.3944 | 0.6375 | | 0.3523 | | 1.001 |
| FR298744 | 4.22E-14 | Exclusively expressed in tumo | 1.1306 | 0.6909 | 0.1473 | | 0.9721 | | | 0.8411 | | 0.5772 |
| FR090905 | 4.85E-14 | | | | | | | | | | | |
| FR401918 | 5.06E-14 | | | | | | | | | | | |
| FR163199 | 7.54E-14 | | | | | | | | | | | |
| FR248128 | 9.10E-14 | Exclusively expressed in tumo | 0.8047 | | 0.5055 | | 0.9234 | 2.4083 | 0.8023 | 0.9486 | 0.4814 | 1.0933 |
| FR149081 | 1.10E-13 | Exclusively expressed in tumo | 0.4194 | | | | 0.3874 | 0.6341 | | 0.3891 | | 1.0438 |
| FR156205 | 1.10E-13 | Exclusively expressed in tumo | 0.5777 | 1.2707 | 0.1538 | | 0.566 | 0.9035 | | 0.9616 | 0.5116 | 1 |
| FR157961 | 1.38E-13 | Exclusively expressed in tumo | 2.2684 | 1.2812 | 0.3451 | | 1.0835 | 1.8171 | | 4.4058 | 0.6357 | 3.1058 |
| FR300684 | 1.64E-13 | | | | | | | | | | | |
| FR376350 | 2.93E-13 | | | | | | | | | | | |
| FR325367 | 3.79E-13 | | | | | | | | | | | |
| FR169300 | 5.84E-13 | | | | | | | | | | | |
| FR217801 | 5.84E-13 | | | | | | | | | | | |
| FR248059 | 6.20E-13 | Exclusively expressed in tumo | 1.0007 | 1.122 | 0.1582 | | 1.509 | | | 0.6495 | | 1.2846 |
| FR390022 | 7.84E-13 | Exclusively expressed in tumo | 0.4422 | | 0.1187 | | 0.581 | | 0.4729 | 0.6149 | 0.9999 | 1.0181 |
| FR072519 | 8.56E-13 | | | | | | | | | | | |
| FR106597 | 9.06E-13 | Exclusively expressed in tumo | 1.9027 | | 0.3604 | | 0.7921 | 0.8452 | | 1.3166 | | 1.1551 |
| FR194863 | 1.67E-12 | | | | | | | | | | | |
| FR105027 | 2.29E-12 | | | | | | | | | | | |
| FR341694 | 2.31E-12 | | | | | | | | | | | |
| FR019031 | 3.52E-12 | Exclusively expressed in tumo | 1.0666 | | | 1.8779 | 0.7689 | 0.7536 | | | 0.875 | 3.6347 |
| FR400444 | 4.33E-12 | Exclusively expressed in tumo | 1.4391 | 1.3957 | 0.567 | | 1.0608 | 2.3207 | | 1.8655 | | 4.189 |
| FR338575 | 4.73E-12 | Exclusively expressed in tumors | | | 0.2374 | 1.4614 | 0.8634 | | | 1.2068 | 0.57 | 2.0257 |
| FR013040 | 5.88E-12 | Exclusively expressed in tumo | 0.8183 | | 0.4493 | | 0.7184 | 1.1539 | | 0.8689 | | 1.8549 |
| FR136623 | 6.00E-12 | | | | | | | | | | | |
| FR001944 | 6.05E-12 | Exclusively expressed in tumo | 0.8175 | 1.4268 | 0.1275 | | 0.7886 | | | | | 1.11 |
| FR310293 | 6.59E-12 | | | | | | | | | | | |
| FR337144 | 8.96E-12 | Exclusively expressed in tumo | 1.6054 | | 0.2044 | | 1.0445 | 0.8538 | | 0.9283 | 0.7551 | 1.3042 |
| FR365501 | 1.37E-11 | | | | | | | | | | | |
| FR236484 | 1.42E-11 | | | | | | | | | | | |
| FR394703 | 1.50E-11 | Exclusively expressed in tumo | 1.3965 | | 0.1868 | | 0.6943 | 0.9173 | | 0.9718 | 0.5026 | 1.7593 |
| FR184567 | 2.39E-11 | | | | | | | | | | | |
| FR143879 | 3.79E-11 | | | | | | | | | | | |
| FR026803 | 5.53E-11 | Exclusively expressed in tumo | 0.9934 | | 0.1922 | | 0.8179 | 1.7757 | | 0.9821 | 2.0972 | 4.7899 |
| FR030135 | 6.18E-11 | Exclusively expressed in tumo | 1.3656 | | 0.1692 | | | 0.5705 | | 0.959 | 0.7178 | 1.4844 |
| FR249679 | 6.59E-11 | | | | | | | | | | | |
| FR252245 | 9.84E-11 | Exclusively expressed in tumors | | | | | 0.745 | 0.7935 | | | | |
| FR252137 | 1.07E-10 | | | | | | | | | | | |
| FR404492 | 1.26E-10 | Exclusively expressed in tumors | | | 0.1429 | | | 1.1407 | | 0.9324 | 2.6606 | 1.4976 |
| FR165524 | 1.98E-10 | Exclusively expressed in tumo | 2.7363 | | 0.4725 | | 0.9784 | 2.2549 | | 2.997 | | 3.1647 |
| FR377799 | 3.72E-10 | Exclusively expressed in tumo | 0.674 | 1.1796 | 0.778 | | | 1.0982 | | | | |
| FR355867 | 6.27E-10 | | | | | | | | | | | |
| FR293228 | 9.08E-10 | | | | | | | | | | | |
| FR074386 | 1.03E-09 | | | | | | | | | | | |
| FR070720 | 1.11E-09 | Exclusively expressed in tumo | 0.7477 | | | | 0.5812 | 0.5589 | | | 0.4224 | 0.6679 |
| FR341404 | 1.13E-09 | Exclusively expressed in tumo | 0.583 | 1.6628 | 0.1802 | | 1.0426 | | | 0.9388 | | 0.9523 |

| | | | | | | | | | | | | |
|---|---|---|---|---|---|---|---|---|---|---|---|---|
| FR281234 | 1.87E-09 | Exclusively expressed in tumo | 1.1558 | | 0.1385 | | 0.6693 | | | 1.2181 | 0.6006 | 2.422 |
| FR258764 | 6.49E-09 | | | | | | | | | | | |
| FR091055 | 1.08E-08 | | | | | | | | | | | |
| FR290353 | 1.08E-08 | | | | | | | | | | | |
| FR207372 | 1.39E-08 | | | | | | | | | | | |
| FR301922 | 1.68E-08 | Exclusively expressed in tumors | | 1.1384 | | | | 1.0693 | | | | 0.8381 |
| FR296497 | 1.90E-08 | | | | | | | | | | | |
| FR403738 | 1.94E-08 | | | | | | | | | | | |
| FR295300 | 2.48E-08 | Exclusively expressed in tumors | | | 0.1604 | | 0.812 | 0.8151 | | | 0.4196 | 0.6764 |
| FR161271 | 2.85E-08 | | | | | | | | | | | |
| FR214430 | 3.07E-08 | | | | | | | | | | | |
| FR042730 | 3.81E-08 | | | | | | | | | | | |
| FR167222 | 3.96E-08 | Exclusively expressed in tumo | 0.5585 | | | | | | 0.6721 | 0.8803 | 0.6504 | 2.2254 |
| FR145670 | 4.02E-08 | | | | | | | | | | | |
| FR126778 | 4.19E-08 | Exclusively expressed in tumo | 0.6908 | | 0.1495 | | 0.6462 | 0.7863 | | | | 0.7591 |
| FR398935 | 7.39E-08 | Exclusively expressed in tumo | 0.5826 | | | | 0.4712 | | 0.6292 | | 0.4403 | |
| FR009646 | 7.98E-08 | | | | | | | | | | | |
| FR162939 | 9.53E-08 | Exclusively expressed in tumo | 0.6032 | | | | | | 0.7633 | | 0.6246 | 0.8149 |
| FR014504 | 9.71E-08 | Exclusively expressed in tumors | | | 0.1824 | | 0.5441 | 0.6383 | | 0.7087 | | 0.9584 |
| FR122719 | 1.21E-07 | Exclusively expressed in tumo | 0.6689 | | 0.2835 | | 1.0481 | 0.7351 | | | | |
| FR289805 | 1.36E-07 | Exclusively expressed in tumo | 0.8853 | | 0.2286 | | 0.6982 | | | | | 1.3129 |
| FR111727 | 1.45E-07 | | | | | | | | | | | |
| FR167602 | 1.51E-07 | Exclusively expressed in tumo | 0.847 | | 0.1538 | | 0.6807 | | | | 0.6754 | 1.1775 |
| FR175260 | 1.53E-07 | | | | | | | | | | | |
| FR218589 | 1.58E-07 | Exclusively expressed in tumo | 0.7362 | | | | | 1.286 | | 0.9343 | 0.489 | 1.7699 |
| FR152981 | 2.06E-07 | Exclusively expressed in tumo | 0.6491 | | 0.1495 | | 0.5289 | 0.8554 | | | | 0.883 |
| FR148018 | 2.35E-07 | Exclusively expressed in tumo | 0.7159 | | 0.3134 | | | 0.77 | | 0.7469 | | 0.7579 |
| FR246461 | 2.90E-07 | Exclusively expressed in tumo | 3.8785 | | 0.4945 | | 4.1204 | 2.3802 | | | | 1.3618 |
| FR019162 | 2.92E-07 | Exclusively expressed in tumors | | | | | 0.6724 | | | 0.7421 | 0.8599 | |
| FR298686 | 2.92E-07 | Exclusively expressed in tumors | | 1.4692 | | | | | | | | 1.0658 |
| FR095987 | 3.69E-07 | Exclusively expressed in tumo | 0.7631 | | 0.2066 | | | 0.9778 | | 1.1411 | | 1.3702 |
| FR301191 | 3.87E-07 | | | | | | | | | | | |
| FR305534 | 1.14E-06 | | | | | | | | | | | |
| FR359895 | 1.54E-06 | Exclusively expressed in tumo | 0.7131 | | | | 0.4179 | | | | | 1.3485 |
| FR215891 | 2.20E-06 | Exclusively expressed in tumo | 0.6878 | | | | 0.807 | | | | | 1.0968 |
| FR205670 | 2.38E-06 | | | | | | | | | | | |
| FR087630 | 2.46E-06 | | | | | | | | | | | |
| FR227782 | 3.40E-06 | | | | | | | | | | | |
| FR079959 | 5.87E-06 | Exclusively expressed in tumo | 0.5082 | 0.8998 | | | | | | | | 1.2636 |
| FR158689 | 6.12E-06 | Exclusively expressed in tumo | 0.6467 | | | | 0.6267 | | | | | 1.7737 |
| FR290641 | 6.69E-06 | Exclusively expressed in tumo | 0.909 | | | | 0.6517 | | | 0.8031 | | 0.7574 |
| FR151159 | 7.16E-06 | | | | | | | | | | | |
| FR026107 | 7.17E-06 | Exclusively expressed in tumo | 0.1766 | | | | | 0.3234 | | | | 0.5447 |
| FR065160 | 8.24E-06 | | | | | | | | | | | |
| FR265415 | 9.26E-06 | | | | | | | | | | | |
| FR025280 | 1.06E-05 | Exclusively expressed in tumo | 0.46 | | | | 0.8758 | | 0.7289 | 0.6968 | | |
| FR010002 | 1.06E-05 | | | | | | | | | | | |

| | | | | | | | | | | | | |
|---|---|---|---|---|---|---|---|---|---|---|---|---|
| FR135669 | 1.31E-05 | | | | | | | | | | | |
| FR332156 | 1.38E-05 | | | | | | | | | | | |
| FR338284 | 1.56E-05 | Exclusively expressed in tumo | 0.7853 | | 0.1648 | | | | | 1.1569 | | 1.3545 |
| FR052968 | 1.80E-05 | | | | | | | | | | | |
| FR111293 | 1.85E-05 | | | | | | | | | | | |
| FR321895 | 2.30E-05 | Exclusively expressed in tumo | 1.5835 | | 0.8163 | | 4.7032 | 3.5136 | | 2.194 | | |
| FR328549 | 2.46E-05 | Exclusively expressed in tumors | | | | | 0.6259 | | | | 0.698 | |
| FR355504 | 2.89E-05 | Exclusively expressed in tumo | 0.6969 | | 0.1341 | | | | | 0.9223 | | 1.2457 |
| FR337428 | 4.48E-05 | | | | | | | | | | | |
| FR314551 | 5.14E-05 | Exclusively expressed in tumors | | | | | 0.9222 | | | | 0.7662 | |
| FR396827 | 5.37E-05 | Exclusively expressed in tumo | 0.4787 | | | | | | | 0.6981 | | 1.4411 |
| FR322380 | 5.42E-05 | Exclusively expressed in tumors | | 4.411 | | 3.7268 | | | 1.3941 | | | 2.2445 |
| FR004109 | 5.73E-05 | Exclusively expressed in tumo | 1.1129 | 3.1131 | 0.2637 | | 0.6698 | 1.1895 | 1.6588 | | 0.729 | 2.1324 |
| FR190502 | 6.23E-05 | Exclusively expressed in tumo | 0.5844 | | | | 0.8193 | | | | | 0.7622 |
| FR148093 | 6.99E-05 | | | | | | | | | | | |
| FR293687 | 7.15E-05 | | | | | | | | | | | |
| FR390460 | 8.85E-05 | | | | | | | | | | | |
| FR337999 | 9.47E-05 | | | | | | | | | | | |
| FR270038 | 0.00010989 | Exclusively expressed in tumors | | | | | 0.6313 | | | | 0.4115 | |
| FR354968 | 0.00011458 | Exclusively expressed in tumo | 0.8789 | | 0.1297 | | | | | | | 0.7835 |
| FR016548 | 0.00013317 | | | | | | | | | | | |
| FR222326 | 0.00013934 | | | | | | | | | | | |
| FR002120 | 0.00013943 | Exclusively expressed in tumors | | | | | 0.9052 | | | | 0.7408 | |
| FR092610 | 0.00014746 | Exclusively expressed in tumo | 0.8983 | | 0.1363 | | 0.597 | | | | | |
| FR322030 | 0.00014861 | Exclusively expressed in tumo | 0.4312 | | | | | | 0.3876 | | | 1.1654 |
| FR369781 | 0.00018264 | Exclusively expressed in tumors | | | 0.1429 | | 0.6235 | | | | 0.4514 | |
| FR154596 | 0.00023936 | Exclusively expressed in tumors | | | | | 1.0077 | 1.0089 | | | | |
| FR138193 | 0.00024458 | Exclusively expressed in tumors | | | | | 0.5786 | | | | 0.4278 | |
| FR066386 | 0.00026381 | Exclusively expressed in tumo | 0.5786 | | | | 0.8349 | | | | | 0.9385 |
| FR304927 | 0.00027112 | Exclusively expressed in tumo | 0.5665 | | | | 0.5233 | | | 1.2133 | | 0.7537 |
| FR045157 | 0.00027708 | | | | | | | | | | | |
| FR089006 | 0.00028435 | | | | | | | | | | | |
| FR319444 | 0.00029535 | | | | | | | | | | | |
| FR288536 | 0.00033214 | | | | | | | | | | | |
| FR152361 | 0.00033487 | Exclusively expressed in tumors | | | 0.156 | | | 0.9401 | | | | 0.7591 |
| FR309811 | 0.00033512 | | | | | | | | | | | |
| FR218180 | 0.00034025 | | | | | | | | | | | |
| FR090658 | 0.0003817 | Exclusively expressed in tumo | 0.5646 | | | | | | | | | 1.3205 |
| FR077664 | 0.00041277 | Exclusively expressed in tumo | 0.627 | | 0.211 | | | | | | | 0.8176 |
| FR308866 | 0.00043245 | Exclusively expressed in tumo | 0.855 | | 0.1429 | | | | | | | 1.1824 |
| FR121747 | 0.0004474 | Exclusively expressed in tumo | 0.7108 | | | | | | | 2.9119 | 0.9212 | |
| FR224235 | 0.0005116 | Exclusively expressed in tumo | 0.5204 | | 0.222 | | | | | 0.9147 | | 0.8333 |
| FR239801 | 0.0005116 | Exclusively expressed in tumo | 0.7125 | | 0.1209 | | | | | | | 0.7479 |
| FR250127 | 0.00055128 | Exclusively expressed in tumo | 0.6642 | | | | | | | 0.7753 | | 1.254 |
| FR028930 | 0.00065834 | Exclusively expressed in tumors | | | | | 0.499 | | | | | 0.7949 |
| FR002829 | 0.00067945 | | | | | | | | | | | |
| FR326119 | 0.00073627 | | | | | | | | | | | |

| | | | | | | | | | | | | |
|---|---|---|---|---|---|---|---|---|---|---|---|---|
| FR127490 | 0.00076369 | | | | | | | | | | | |
| FR066510 | 0.00081035 | | | | | | | | | | | |
| FR342305 | 0.00083219 | Exclusively expressed in tumo | 0.9013 | | 0.2923 | | | 0.9526 | | | | |
| FR236081 | 0.0008544 | Exclusively expressed in tumo | 0.7649 | | | | 0.8218 | | | | | |
| FR243247 | 0.00086729 | | | | | | | | | | | |
| FR048796 | 0.0010731 | Exclusively expressed in tumors | | | | | | | | | 1.005 | |
| FR302308 | 0.00109836 | Exclusively expressed in tumors | | | | | | | 1.6182 | 0.6688 | | |
| FR290142 | 0.00110561 | Exclusively expressed in tumo | 2.4363 | 6.5092 | | | | 9.2501 | | | | 2.8365 |
| FR064000 | 0.00116585 | | | | | | | | | | | |
| FR159092 | 0.00117402 | Exclusively expressed in tumo | 0.6138 | | 0.222 | | | | | | | 0.7551 |
| FR123582 | 0.00119817 | Exclusively expressed in tumors | | | | | 0.6418 | | | | | 0.8708 |
| FR316592 | 0.00134275 | Exclusively expressed in tumors | | | | | | | | | 0.5955 | 0.8756 |
| FR099055 | 0.00134334 | Exclusively expressed in tumors | | | | | | | | 1.1083 | 0.4382 | |
| FR267703 | 0.00140188 | Exclusively expressed in tumo | 0.9136 | | | | | | | 1.0927 | | |
| FR173465 | 0.0014276 | Exclusively expressed in tumo | 0.7735 | | 0.1868 | | | | | | | 1.0793 |
| FR061877 | 0.00144454 | Exclusively expressed in tumors | | | | | | | | | 0.6355 | 1.2208 |
| FR060745 | 0.00147273 | Exclusively expressed in tumors | | | | | 0.5525 | | | | | 0.6767 |
| FR207523 | 0.00155135 | Exclusively expressed in tumors | | | 0.2571 | | | 0.945 | | | | 0.908 |
| FR089602 | 0.00157569 | Exclusively expressed in tumors | | | | | | 0.8951 | | | | 0.9886 |
| FR115804 | 0.00159482 | Exclusively expressed in tumors | | | | | | 0.8439 | | | | 0.7142 |
| FR162794 | 0.00239611 | Exclusively expressed in tumo | 0.2861 | | | | | | | 0.2706 | | |
| FR202470 | 0.00239611 | Exclusively expressed in tumo | 0.2861 | | | | | | | 0.2706 | | |
| FR255519 | 0.00241364 | Exclusively expressed in tumors | | | | | 0.7284 | | | | 0.6149 | |
| FR132879 | 0.00252223 | | | | | | | | | | | |
| FR344447 | 0.00277426 | Exclusively expressed in tumo | 0.3326 | | | | | | | 0.2908 | | |
| FR389121 | 0.00311307 | Exclusively expressed in tumo | 0.9068 | | 0.1604 | | | 1.1292 | | | | |
| FR295545 | 0.00319292 | Exclusively expressed in tumors | | | | | | | 2.0497 | | | |
| FR324841 | 0.00332569 | Exclusively expressed in tumors | | | 0.1495 | | | | | 0.9549 | | 0.8159 |
| FR166990 | 0.00341571 | Exclusively expressed in tumo | 0.8737 | | | | | | | 0.7288 | | 1.1389 |
| FR118220 | 0.00387302 | | | | | | | | | | | |
| FR315126 | 0.00388773 | Exclusively expressed in tumors | | | | | | 0.5283 | | | | |
| FR298737 | 0.00390864 | Exclusively expressed in tumors | | 4.2829 | | 3.5559 | 2.0276 | | | | | |
| FR221589 | 0.00405339 | Exclusively expressed in tumors | | | | | | 0.3343 | | | | 0.6645 |
| FR372325 | 0.00433168 | | | | | | | | | | | |
| FR205900 | 0.00472191 | Exclusively expressed in tumors | | | | | | | | | 0.6074 | |
| FR109932 | 0.0048362 | | | | | | | | | | | |
| FR221812 | 0.00523338 | Exclusively expressed in tumors | | | | | | | | | 0.5403 | |
| FR026913 | 0.00588347 | | | | | | | | | | | |
| FR217228 | 0.00620675 | | | | | | | | | | | |
| FR083863 | 0.00696215 | Exclusively expressed in tumors | | | | | | | 0.483 | | | 0.7524 |
| FR350968 | 0.00723115 | Exclusively expressed in tumo | 0.4713 | | | | 0.4638 | | | | | |
| FR274844 | 0.00822044 | Exclusively expressed in tumors | | | | | 1.2423 | | | | | |
| FR330790 | 0.00860029 | Exclusively expressed in tumors | | | | | | | | | 0.5072 | |
| FR134850 | 0.00942974 | Exclusively expressed in tumors | | | 0.1934 | | | | | | 0.7866 | |
| FR227926 | 0.00952981 | Exclusively expressed in tumors | | | | 1.5625 | | | | | | |

# Supplementary Table 4

## Clinical features associated with sub-clusters in HNSC

### Multivariate analysis

| | Df | Sum Sq | Mean Sq | F value | Pr(>F) | |
|---|---|---|---|---|---|---|
| Alcohol consuption | 2 | 1.045 | 0.5223 | 1.0047 | 0.36724 | |
| Anatomical site | 12 | 4.911 | 0.4092 | 0.7873 | 0.663683 | |
| Nodal status | 2 | 6.552 | 3.2758 | 6.302 | 0.002055 | ** |
| Tumor stage | 4 | 5.035 | 1.2587 | 2.4216 | 0.048165 | * |
| hpv presence | 2 | 0.767 | 0.3835 | 0.7377 | 0.478969 | |
| Tumor grade | 4 | 1.692 | 0.4229 | 0.8136 | 0.517129 | |
| New oral neoplasm | 1 | 0.349 | 0.3487 | 0.6709 | 0.413332 | |
| New T after Tx | 2 | 1.159 | 0.5795 | 1.1149 | 0.329147 | |
| Smoking (packs per years) | 85 | 40.012 | 0.4707 | 0.9056 | 0.704393 | |
| Smoking | 2 | 1.299 | 0.6493 | 1.249 | 0.288105 | |
| Residuals | 338 | 175.695 | 0.5198 | | | |

---

Signif. codes: 0 ‘ ***’ 0.0 01 ‘**’ 0.01 ‘*’ 0.05 ‘.’ 0.1 ‘ ’ 1

### Univariate analysis

| Nodal status | NEG | POS | Information | Total | NEG (%) | POS (%) |
|---|---|---|---|---|---|---|
| Not in a cluster | 162 | 181 | 24 | 343 | 47.23% | 52.77% |
| Cluster 1 | 8 | 11 | 1 | 19 | 42.11% | 57.89% |
| Cluster 2 | 39 | 18 | 11 | 57 | 68.42% | 31.58% |

**p=0.0098**

**higher fraction of node negative in cluster 2**

# Clinical features associated with sub-clusters in KIRC

## Multivariate analysis

| | Df | Sum Sq | Mean Sq | F value | Pr(>F) | |
|---|---|---|---|---|---|---|
| met | 2 | 2.78 | 1.39213 | 1.4014 | 0.24716 | |
| nodal | 2 | 4.35 | 2.17439 | 2.1888 | 0.11305 | |
| factor(stage) | 4 | 10.51 | 2.62812 | 2.6455 | 0.03281 | * |
| VHL | 1 | 2.88 | 2.87946 | 2.8985 | 0.08924 | . |
| Residuals | 538 | 534.46 | 0.99342 | | | |

---

Signif. codes:  0 '***' 0.001 '**' 0.01 '*' 0.05 '.' 0.1 ' ' 1

## Univariate analysis

| | Stage | | | | | % of samples per cluster | | | |
|---|---|---|---|---|---|---|---|---|---|
| | I | II | III | IV | sum | I | II | III | IV |
| Not in a cluste | 104 | 31 | 48 | 39 | 222 | 46.85% | 13.96% | 21.62% | 17.57% |
| Cluster 1 | 86 | 14 | 30 | 12 | 142 | 60.56% | 9.86% | 21.13% | 8.45% |
| Cluster 2 | 60 | 11 | 37 | 18 | 126 | 47.62% | 8.73% | 29.37% | 14.29% |
| Cluster 3 | 18 | 5 | 16 | 10 | 49 | 36.73% | 10.20% | 32.65% | 20.41% |

**p=0.0346**

**Cluster 1 has a higher fraction of Stage I tumours**

# Clinical features associated with sub-clusters in PRAD

## Multivariate analysis

| | Df | Sum Sq | Mean Sq | F value | Pr(>F) |
|---|---|---|---|---|---|
| factor(type) | 2 | 0.9057 | 0.45285 | 4.1022 | 0.017657 |
| nodal | 2 | 0.0023 | 0.00115 | 0.0104 | 0.989674 |
| factor(Tstage) | 3 | 0.1515 | 0.05049 | 0.4574 | 0.71233 |
| gleasonscore | 4 | 1.5747 | 0.39369 | 3.5663 | 0.007523 |
| Residuals | 251 | 27.708 | 0.11039 | | |

---

Signif. codes: 0 '***' 0.001 '**' 0.01 '*' 0.05 '.' 0.1 ' ' 1

## Univariate analysis

| Gleason score | 6 to 7 | 8 to 10 | | 6 to 7 | 8 to 10 |
|---|---|---|---|---|---|
| Not in a cluster | 167 | 56 | 223 | 0.748878924 | 0.251121076 |
| Cluster 1 | 19 | 16 | 35 | 0.542857143 | 0.45714286 |

**p=0.0152**

**More higher gleason scores in cluster 1 group**

## piRNA differentially expressed in breast ductal adenocarcinoma

| piRNA indentifier | BH p-value |
|---|---|
| FR064000 | 0.00118 |
| FR267761 | 0.00118 |
| FR132045 | 0.00118 |
| FR132879 | 0.00118 |
| FR019089 | 0.00118 |
| FR007639 | 0.00118 |
| FR319486 | 0.00118 |
| FR310293 | 0.00118 |
| FR372325 | 0.00118 |
| FR298737 | 0.00118 |
| FR028698 | 0.00118 |
| FR290353 | 0.00118 |
| FR019019 | 0.00118 |
| FR090905 | 0.00118 |
| FR324541 | 0.00118 |
| FR290142 | 0.00118 |
| FR095339 | 0.00118 |
| FR169300 | 0.00118 |
| FR217801 | 0.00118 |
| FR210409 | 0.00118 |
| FR237180 | 0.00118 |
| FR163199 | 0.00118 |
| FR016773 | 0.00118 |
| FR091055 | 0.00118 |
| FR271551 | 0.00118 |
| FR233730 | 0.00118 |
| FR243752 | 0.00118 |
| FR107946 | 0.00118 |
| FR190827 | 0.00118 |
| FR136623 | 0.00118 |
| FR359148 | 0.00118 |
| FR378984 | 0.00118 |
| FR393260 | 0.00118 |
| FR355867 | 0.00118 |
| FR205670 | 0.00118 |
| FR197889 | 0.00118 |
| FR103462 | 0.00118 |
| FR218523 | 0.00118 |

| | |
|---|---|
| FR365646 | 0.00118 |
| FR114004 | 0.00118 |
| FR380286 | 0.002098 |
| FR266905 | 0.002098 |
| FR306048 | 0.002098 |
| FR140858 | 0.002098 |
| FR349959 | 0.002098 |
| FR268871 | 0.002949 |
| FR075316 | 0.002949 |
| FR263507 | 0.002949 |
| FR324515 | 0.003701 |
| FR246367 | 0.003701 |
| FR338565 | 0.003701 |
| FR259362 | 0.005342 |
| FR255141 | 0.005342 |
| FR262980 | 0.006991 |
| FR297613 | 0.007722 |
| FR283482 | 0.008136 |
| FR006353 | 0.008136 |
| FR003837 | 0.008136 |
| FR035643 | 0.009598 |

# Supplementary Table 5: piRNAs associated with survival

**P-values**

| **piRNA_list** | **BLCA** | **BRCA** | **COAD** | **HNSC** | **KIRC** | **LUAD** | **LUSC** | **PRAD** | **STAD** | **UCEC** | **THCA** |
|---|---|---|---|---|---|---|---|---|---|---|---|
| FR004819 | 0.0036 | | | | | | | | 0.0199 | | 0.0483 |
| FR006353 | | | 0.0424 | 0.0110 | | | | | | | 0.0210 |
| FR025321 | | 0.0424 | | 0.0069 | | | | | | 0.0005 | |
| FR027884 | | | | | 0.0000 | | 0.0012 | | | 0.0391 | |
| FR072386 | | 0.0475 | | | 0.0029 | | | | | 0.0454 | |
| FR245310 | | | 0.0023 | 0.0287 | | | | | | | 0.0366 |
| FR262980 | | | | | 0.0000 | | 0.0043 | | | 0.0358 | |
| FR279668 | | 0.0057 | | | 0.0002 | 0.0022 | | | | | |
| FR327232 | | | | | 0.0481 | | 0.0352 | | | 0.0020 | |
| FR381169 | | | | 0.0230 | 0.0000 | | | | | | 0.0420 |
| FR018916 | | | 0.0102 | | 0.0001 | | | | | | |
| FR019019 | | | | 0.0404 | | 0.0411 | | | | | |
| FR027151 | 0.0160 | | 0.0221 | | | | | | | | |
| FR111727 | | | | | 0.0001 | | | | | | 0.0359 |
| FR135042 | | | | | | | 0.0022 | | | 0.0391 | |
| FR140858 | | | | | 0.0002 | | 0.0100 | | | | |
| FR148093 | | 0.0045 | | | | | | | | | 0.0449 |
| FR190827 | | | 0.0107 | | 0.0269 | | | | | | |
| FR218523 | | | 0.0057 | 0.0308 | | | | | | | |
| FR222326 | | | | 0.0036 | | 0.0062 | | | | | |
| FR236484 | | | | 0.0498 | | | | | | 0.0013 | |
| FR236999 | 0.0459 | | | 0.0045 | | | | | | | |
| FR248000 | | | 0.0001 | | | 0.0463 | | | | | |
| FR255141 | | | 0.0022 | | | | | | | 0.0038 | |
| FR264146 | | | 0.0043 | | | | | | | 0.0028 | |
| FR271551 | | 0.0091 | | 0.0087 | | | | | | | |
| FR283482 | | | | | 0.0058 | 0.0020 | | | | | |
| FR293713 | | | 0.0104 | | 0.0000 | | | | | | |
| FR296333 | | 0.0093 | | | | | | | | 0.0108 | |
| FR301191 | 0.0366 | | 0.0240 | | | | | | | | |
| FR319465 | | | 0.0111 | | 0.0431 | | | | | | |
| FR319486 | | 0.0234 | | | 0.0285 | | | | | | |
| FR326119 | | | | | 0.0004 | | | | | 0.0305 | |
| FR349959 | | | 0.0255 | | 0.0044 | | | | | | |
| FR365501 | 0.0473 | | | | 0.0155 | | | | | | |
| FR378984 | | 0.0193 | | 0.0013 | | | | | | | |
| FR016773 | | | 0.0023 | | | | | | | | |
| FR026913 | | | 0.0078 | | | | | | | | |
| FR035643 | | | | | 0.0366 | | | | | | |
| FR038165 | | | | | | 0.0181 | | | | | |

| | | | | | | | | | | | |
|---|---|---|---|---|---|---|---|---|---|---|---|
| FR043670 | | | | 0.0453 | | | | | | | |
| FR074386 | | | | | 0.0004 | | | | | | |
| FR075316 | | | 0.0006 | | | | | | | | |
| FR082269 | 0.0203 | | | | | | | | | | |
| FR091055 | | | | | | | | | | | 0.0264 |
| FR103462 | | | | | 0.0000 | | | | | | |
| FR114004 | | | | | 0.0389 | | | | | | |
| FR125672 | | | 0.0162 | | | | | | | | |
| FR131044 | | | 0.0035 | | | | | | | | |
| FR157678 | | | | 0.0121 | | | | | | | |
| FR163199 | | | 0.0002 | | | | | | | | |
| FR169300 | | | | | 0.0095 | | | | | | |
| FR182987 | | 0.0219 | | | | | | | | | |
| FR197889 | | | | 0.0306 | | | | | | | |
| FR202919 | | | | | | | | | 0.0190 | | |
| FR213383 | | | | | 0.0325 | | | | | | |
| FR213473 | 0.0047 | | | | | | | | | | |
| FR217801 | | | | | 0.0095 | | | | | | |
| FR218973 | | | | | 0.0004 | | | | | | |
| FR246367 | | | | | 0.0031 | | | | | | |
| FR249679 | | | 0.0004 | | | | | | | | |
| FR259362 | | | | | | | | | | | 0.0424 |
| FR266905 | | | | | 0.0065 | | | | | | |
| FR268871 | 0.0042 | | | | | | | | | | |
| FR270731 | | | | | 0.0001 | | | | | | |
| FR297613 | | | 0.0048 | | | | | | | | |
| FR302680 | | | | | 0.0003 | | | | | | |
| FR319444 | | | 0.0004 | | | | | | | | |
| FR324515 | | | | | 0.0332 | | | | | | |
| FR338747 | | | | | | | | | | 0.0178 | |
| FR359148 | | | 0.0054 | | | | | | | | |
| FR365646 | | | | | 0.0001 | | | | | | |

| piRNA | TCGA (log-rank pvalue) | GSE29173 (log-rank pvalue) |
| --- | --- | --- |
| FR072386 | 0.0475 | 0.6476 |
| FR182987 | 0.0219 | not expressed |
| FR271551 | 0.0091 | 0.9829 |
| FR378984 | 0.0193 | 0.0200 |
| FR025321 | 0.0424 | 0.0225 |
| FR296333 | 0.0093 | not expressed |
| FR279668 | 0.0057 | not expressed |
| FR319486 | 0.0234 | not expressed |
| FR148093 | 0.0045 | not expressed |

# Supplementary Table 6

| | Genes | | | mutations (COSMIC) | | | Repetitive elements | | | |
|---|---|---|---|---|---|---|---|---|---|---|
| piRNA ID | Overlapping Features | Nearest Feature | Distance to Nearest Feature (bps) | Overlapping Features | Nearest Feature | Distance to Nearest Feature (bps) | Overlapping Features | Nearest Feature | Class | Distance to Nearest Feature (bps) |
| FR041203 | contained within PRK | PRKCZ (+) | 0 | COSM116523 | COSM116523 | 0 | region is 254 bp dow | Tigger3c (-) | DNA | 254 |
| FR277415 | contained within REF | RERE (-) | 0 | region is 1372 bp fro | COSM4144196 | 1372 | region is 321 bp upst | AT_rich (+) | Low_complexity | 321 |
| FR060745 | contained within H6P | H6PD (+) | 0 | region is 1878 bp fro | COSM1667123 | 1878 | region is 65 bp down | AluSx1 (-) | SINE | 65 |
| FR095987 | contained within RC( | RCC2 (-) | 0 | region is 1879 bp fro | COSM1687088 | 1879 | region is 357 bp dow | AT_rich (+) | Low_complexity | 357 |
| FR202919 | region is 41236 bp do | LOC284632 (+) | 41236 | region is 70155 bp fr | COSM1340932 | 70155 | region overlaps with | Charlie4z (-) | DNA | 0 |
| FR309900 | contained within WA: | WASF2 (-) | 0 | region is 2280 bp fro | COSM1341512 | 2280 | region is 220 bp dow | MIR3 (+) | SINE | 220 |
| FR122719 | contained within PTA | PTAFR (-) | 0 | region is 1571 bp fro | COSM907953 | 1571 | region is 52 bp down | AluSz (-) | SINE | 52 |
| FR218973 | contained within SN( | SNORA61 (-) | 0 | region is 2158 bp fro | COSM426034 | 2158 | region is 221 bp upst | AluSx1 (+) | SINE | 221 |
| FR259362 | contained within SN( | SNORD103B (-) | 0 | region is 903 bp from | COSM3977565 | 903 | region is 396 bp upst | L2c (+) | LINE | 396 |
| FR236484 | contained within SN( | SNORD85 (-) | 0 | region is 224 bp from | COSM258720 | 224 | region is 647 bp dow | L2a (-) | LINE | 647 |
| FR228545 | contained within SN( | SNORD85 (-) | 0 | region is 224 bp from | COSM258720 | 224 | region is 647 bp dow | L2a (-) | LINE | 647 |
| FR073001 | region is 10368 bp up | LINC01225 (+) | 10368 | region is 54416 bp fr | COSM3943862 | 54416 | region is 950 bp upst | THE1B (+) | LTR | 950 |
| FR394703 | contained within KPN | KPNA6 (+) | 0 | region is 1346 bp fro | COSM534627 | 1346 | region is 209 bp dow | (TTC)n (+) | Simple_repeat | 209 |
| FR254828 | contained within AKI | AKIRIN1 (+) | 0 | COSM909134 | COSM909134 | 0 | region is 114 bp dow | MIR (-) | SINE | 114 |
| FR315324 | intron of TMEM125 (+ | TMEM125 (+) | 0 | region is 1821 bp fro | COSM3689701 | 1821 | region is 322 bp dow | MIRb (-) | SINE | 322 |
| FR248059 | contained within DPH | DPH2 (+) | 0 | COSM1250623, COSI | COSM3805357 | 0 | region is 1411 bp dov | AluJb (+) | SINE | 1411 |
| FR006353 | contained within SN( | SNORD38B (+) | 0 | region is 182 bp from | COSM3711354 | 182 | region is 759 bp dow | AluJb (-) | SINE | 759 |
| FR079959 | contained within PIK: | PIK3R3 (-) | 0 | region is 1871 bp fro | COSM189506 | 1871 | region is 2255 bp dov | AluJo (-) | SINE | 2255 |
| FR086093 | region is 14886 bp do | S1PR1 (+) | 14886 | region is 16274 bp fr | COSM893037 | 16274 | region is 126 bp dow | AluY (+) | SINE | 126 |
| FR135669 | contained within CSD | CSDE1 (-) | 0 | region is 8298 bp fro | COSM1472449 | 8298 | region is 942 bp dow | L2a (-) | LINE | 942 |
| FR128806 | region is 46591 bp up | VANGL1 (+) | 46591 | region is 64279 bp fr | COSM1270142 | 64279 | region is 786 bp upst | AluJo (+) | SINE | 786 |
| FR248128 | contained within PH( | PHGDH (+) | 0 | COSM1723652 | COSM1723652 | 0 | region is 372 bp upst | MIR3 (+) | SINE | 372 |
| FR242050 | contained within TXN | TXNIP (+) | 0 | region is 270 bp from | COSM1626380 | 270 | region is 224 bp dow | C-rich (+) | Low_complexity | 224 |
| FR324841 | contained within HIS | HIST2H2AA4 (-) | 0 | region is 1371 bp fro | COSM3930377 | 1371 | region is 288 bp dow | (TCCCG)n (+) | Simple_repeat | 288 |
| FR324841_dup1 | contained within HIS | HIST2H2AA4 (+) | 0 | region is 82 bp from | COSM1333712 | 82 | region is 288 bp upst | (CGGGA)n (+) | Simple_repeat | 288 |
| FR387105 | region is 624 bp dow | NBPF18P (+) | 624 | region is 9586 bp fro | COSM277147 | 9586 | region is 476 bp dow | L2b (-) | LINE | 476 |
| FR298737 | intron of KIAA0907 (- | SNORA80E (-) | 0 | region is 1344 bp fro | COSM675700 | 1344 | region is 110 bp upst | AluY (+) | SINE | 110 |
| FR175260 | intron of KIAA0907 (- | SNORA80E (-) | 0 | region is 1344 bp fro | COSM675700 | 1344 | region is 110 bp upst | AluY (+) | SINE | 110 |
| FR136176 | contained within ISG | ISG20L2 (-) | 0 | region is 308 bp from | COSM1334920 | 308 | region is 152 bp upst | AluSq2 (-) | SINE | 152 |
| FR361464 | contained within TOF | TOR1AIP2 (-) | 0 | region is 1445 bp fro | COSM1134779 | 1445 | region is 13 bp upstr | MLT1L (+) | LTR | 13 |
| FR106749 | contained within ZNF | ZNF648 (-) | 0 | COSM1744823, COSI | COSM1744823 | 0 | region is 1236 bp dov | AluSg7 (-) | SINE | 1236 |
| FR242943 | contained within WD | WDR37 (+) | 0 | region is 2040 bp fro | COSM3806508 | 2040 | region is 340 bp upst | (TG)n (+) | Simple_repeat | 340 |
| FR338284 | contained within HNF | HNRNPF (-) | 0 | COSM1675184, COSI | COSM1675185 | 0 | region is 718 bp upst | HAL1 (+) | LINE | 718 |
| FR014504 | contained within TIM | TIMM23 (-) | 0 | region is 287 bp from | COSM3358772 | 287 | region is 232 bp upst | AluSx (-) | SINE | 232 |
| FR103462 | contained within SN( | SNORD98 (+) | 0 | region is 132 bp from | COSM236069 | 132 | region is 638 bp dow | AluYc (+) | SINE | 638 |
| FR365646 | contained within SN( | SNORD98 (+) | 0 | region is 132 bp from | COSM236069 | 132 | region is 639 bp dow | AluYc (+) | SINE | 639 |
| FR074386 | contained within SN( | SNORD98 (+) | 0 | region is 132 bp from | COSM236069 | 132 | region is 640 bp dow | AluYc (+) | SINE | 640 |
| FR206428 | contained within EIF4 | EIF4EBP2 (+) | 0 | region is 4201 bp fro | COSM1505796 | 4201 | region is 293 bp dow | AluSz (-) | SINE | 293 |
| FR400444 | contained within SE( | SEC24C (+) | 0 | COSM920382 | COSM920382 | 0 | region is 906 bp upst | AluSz6 (+) | SINE | 906 |
| FR013040 | contained within EIF! | EIF5AL1 (+) | 0 | COSM920752, COSM | COSM920753 | 0 | region is 468 bp dow | (CGG)n (+) | Simple_repeat | 468 |
| FR148018 | region is 24522 bp do | MAT1A (-) | 24522 | region is 56 bp from | COSM3981161 | 56 | region is 346 bp dow | (A)n (+) | Simple_repeat | 346 |
| FR013040_dup1 | region is 24519 bp do | MAT1A (-) | 24519 | region is 53 bp from | COSM3981161 | 53 | region is 349 bp dow | (A)n (+) | Simple_repeat | 349 |
| FR037883 | contained within LIN( | LINC00865 (+) | 0 | region is 64981 bp fr | COSM198308 | 64981 | region is 594 bp dow | Charlie1b (-) | DNA | 594 |
| FR295300 | contained within TME | TMEM180 (+) | 0 | COSM1268059, COSI | COSM200259 | 0 | region is 224 bp dow | MIR (+) | SINE | 224 |
| FR296810 | contained within FAM | FAM53B (-) | 0 | region is 1347 bp fro | COSM87431 | 1347 | region is 1928 bp ups | L1M5 (+) | LINE | 1928 |
| FR006894 | contained within SN( | SNORA54 (-) | 0 | region is 830 bp from | COSM687613 | 830 | region is 666 bp dow | L1MEg (+) | LINE | 666 |
| FR105027 | contained within SN( | SNORA45A (+) | 0 | region is 154 bp from | COSM166780 | 154 | region is 245 bp upst | AluSx (-) | SINE | 245 |
| FR061350 | contained within RPL | RPL27A (+) | 0 | COSM1357578 | COSM1357578 | 0 | region is 746 bp upst | AluSx (-) | SINE | 746 |
| FR035643 | contained within MTF | MTRNR2L8 (-) | 0 | region is 3027 bp fro | COSM685708 | 3027 | region is 601 bp upst | LSU-rRNA_Hsa (-) | rRNA | 601 |

| | | | |
|---|---|---|---|
| FR169300 | region is 900 bp upst | MIR4485 (-) | 900 |
| FR217801 | region is 900 bp upst | MIR4485 (-) | 900 |
| FR111727 | region is 1066 bp ups | MIR4485 (-) | 1066 |
| FR319486 | region is 1469 bp dov | RNF141 (-) | 1469 |
| FR227782 | region is 1469 bp dov | RNF141 (-) | 1469 |
| FR019031 | contained within EIF4 | EIF4G2 (-) | 0 |
| FR021452 | region is 6020 bp ups | LOC100507384 (+) | 6020 |
| FR041836 | region is 245 bp upst | LOC100507384 (+) | 245 |
| FR065160 | contained within SNO | SNORD67 (-) | 0 |
| FR337552 | contained within SNO | SNORD67 (-) | 0 |
| FR049394 | contained within TMX | TMX2-CTNND1 (+) | 0 |
| FR341449 | contained within DTX | DTX4 (+) | 0 |
| FR026803 | contained within FTH | FTH1 (-) | 0 |
| FR217789 | contained within FTH | FTH1 (-) | 0 |
| FR223921 | contained within AHN | AHNAK (-) | 0 |
| FR223921_dup1 | contained within AHN | AHNAK (-) | 0 |
| FR281234 | contained within EEF | EEF1G (-) | 0 |
| FR352701 | contained within MRI | MRPL49 (+) | 0 |
| FR325367 | contained within LTB | LTBP3 (-) | 0 |
| FR295545 | contained within EFE | EFEMP2 (-) | 0 |
| FR302308 | contained within LOC | LOC100128494 (+) | 0 |
| FR199846 | contained within RNF | RNF169 (+) | 0 |
| FR364055 | region is 2282 bp dov | NEU3 (+) | 2282 |
| FR380227 | contained within SNO | SNORD15A (+) | 0 |
| FR002829 | intron of SMCO4 (-) | SMCO4 (-) | 0 |
| FR190827 | contained within SNO | SNORD6 (-) | 0 |
| FR290353 | contained within SNO | SNORD6 (-) | 0 |
| FR157678 | contained within MIR | MIR34B (+) | 0 |
| FR387750 | contained within MIR | MIR34B (+) | 0 |
| FR224609 | contained within HYL | HYLS1 (+) | 0 |
| FR098442 | intron of IGSF9B (-) | IGSF9B (-) | 0 |
| FR271321 | contained within NCA | NCAPD3 (-) | 0 |
| FR344972 | contained within M6P | M6PR (-) | 0 |
| FR138455 | contained within M6P | M6PR (-) | 0 |
| FR013706 | contained within M6P | M6PR (-) | 0 |
| FR233702 | region is 21747 bp do | LOC642846 (+) | 21747 |
| FR278590 | region is 21747 bp do | LOC642846 (+) | 21747 |
| FR073001_dup1 | intron of AEBP2 (+) | AEBP2 (+) | 0 |
| FR221242 | contained within CCD | CCDC91 (+) | 0 |
| FR283571 | contained within TME | TMBIM6 (+) | 0 |
| FR372317 | contained within PFD | PFDN5 (+) | 0 |
| FR019162 | contained within AAA | AAAS (-) | 0 |
| FR138455_dup1 | region is 8613 bp dov | OR6C65 (+) | 8613 |
| FR233520 | contained within RPL | RPL41 (+) | 0 |
| FR215891 | contained within CTD | CTDSP2 (-) | 0 |
| FR386994 | contained within CTD | CTDSP2 (-) | 0 |
| FR250127 | contained within GNS | GNS (-) | 0 |
| FR233520_dup1 | intron of LOC643339 | LOC643339 (-) | 0 |
| FR338565 | region is 4692 bp ups | NEDD1 (+) | 4692 |
| FR361123 | contained within GOI | GOLGA2P5 (-) | 0 |
| FR129496 | intron of TXNRD1 (+) | TXNRD1 (+) | 0 |
| FR285125 | contained within UBE | UBE3B (+) | 0 |

| | | |
|---|---|---|
| region is 3357 bp fro | COSM685708 | 3357 |
| region is 3357 bp fro | COSM685708 | 3357 |
| region is 3523 bp fro | COSM685708 | 3523 |
| region is 4307 bp fro | COSM685708 | 4307 |
| region is 4313 bp fro | COSM685708 | 4313 |
| region is 277 bp from | COSM3666846 | 277 |
| region is 65375 bp fr | COSM1508266 | 65375 |
| region is 71152 bp fr | COSM1508266 | 71152 |
| region is 153 bp from | COSM1683191 | 153 |
| region is 154 bp from | COSM1683191 | 154 |
| COSM360760, COSM | COSM928698 | 0 |
| region is 2090 bp fro | COSM689582 | 2090 |
| region is 4 bp from C | COSM3397977 | 4 |
| region is 4 bp from C | COSM70835 | 4 |
| COSM3809806 | COSM3809806 | 0 |
| COSM1638108, COSI | COSM273920 | 0 |
| COSM193579, COSM | COSM929695 | 0 |
| COSM1746461 | COSM1746461 | 0 |
| COSM1355946 | COSM1355946 | 0 |
| region is 7 bp from C | COSM1127682 | 7 |
| COSM1746548 | COSM1746548 | 0 |
| region is 2446 bp fro | COSM1676303 | 2446 |
| region is 3552 bp fro | COSM336405 | 3552 |
| region is 284 bp from | COSM290699 | 284 |
| region is 56422 bp fr | COSM933341 | 56422 |
| region is 1257 bp fro | COSM933415 | 1257 |
| region is 1294 bp fro | COSM933415 | 1294 |
| region is 1823 bp fro | COSM1585386 | 1823 |
| region is 1822 bp fro | COSM1585386 | 1822 |
| region is 6138 bp fro | COSM924571 | 6138 |
| region is 5916 bp fro | COSM1352695 | 5916 |
| region is 20 bp from | COSM1297866 | 20 |
| region is 1217 bp fro | COSM944343 | 1217 |
| region is 785 bp from | COSM944343 | 785 |
| region is 592 bp from | COSM944343 | 592 |
| region is 23166 bp fr | COSM1323007 | 23166 |
| region is 23166 bp fr | COSM1323007 | 23166 |
| region is 5205 bp fro | COSM3811641 | 5205 |
| region is 4 bp from C | COSM388093 | 4 |
| region is 861 bp from | COSM3935983 | 861 |
| COSM1299585, COSI | COSM1299585 | 0 |
| COSM694094 | COSM694094 | 0 |
| region is 1616 bp fro | COSM941294 | 1616 |
| region is 158 bp from | COSM431483 | 158 |
| COSM1202573 | COSM1202573 | 0 |
| region is 478 bp from | COSM1363457 | 478 |
| region is 30 bp from | COSM1363634 | 30 |
| region is 191775 bp f | COSM1364872 | 191775 |
| region is 7236 bp fro | COSM3359985 | 7236 |
| region is 8414 bp fro | COSM1745265 | 8414 |
| region is 7624 bp fro | COSM934239 | 7624 |
| COSM1358620, COSI | COSM1510729 | 0 |

| | | | |
|---|---|---|---|
| region is 931 bp upst | LSU-rRNA_Hsa (-) | rRNA | 931 |
| region is 931 bp upst | LSU-rRNA_Hsa (-) | rRNA | 931 |
| region is 1097 bp ups | LSU-rRNA_Hsa (-) | rRNA | 1097 |
| region is 938 bp upst | MER1B (+) | DNA | 938 |
| region is 938 bp upst | MER1B (+) | DNA | 938 |
| region is 665 bp upst | MER5A (+) | DNA | 665 |
| region is 1054 bp dov | AluSx (+) | SINE | 1054 |
| region is 483 bp upst | MIR3 (+) | SINE | 483 |
| region is 976 bp dow | MIRc (+) | SINE | 976 |
| region is 976 bp dow | MIRc (+) | SINE | 976 |
| region is 197 bp dow | MIRb (+) | SINE | 197 |
| region is 414 bp dow | (TA)n (+) | Simple_repeat | 414 |
| region is 805 bp dow | AluSx3 (+) | SINE | 805 |
| region is 810 bp dow | AluSx3 (+) | SINE | 810 |
| region is 9816 bp dov | MIRb (-) | SINE | 9816 |
| region is 8664 bp dov | MIRb (-) | SINE | 8664 |
| region is 502 bp upst | L1MD2 (+) | LINE | 502 |
| region is 186 bp upst | L2b (-) | LINE | 186 |
| region is 49 bp upstr | C-rich (+) | Low_complexity | 49 |
| region is 351 bp upst | MIRc (-) | SINE | 351 |
| region is 874 bp dow | AluSx4 (-) | SINE | 874 |
| region is 123 bp dow | AT_rich (+) | Low_complexity | 123 |
| region is 250 bp upst | L2b (+) | LINE | 250 |
| region is 456 bp dow | AluY (-) | SINE | 456 |
| region is 176 bp upst | (CAG)n (+) | Simple_repeat | 176 |
| region is 1509 bp dov | AT_rich (+) | Low_complexity | 1509 |
| region is 1546 bp dov | AT_rich (+) | Low_complexity | 1546 |
| region is 510 bp dow | C-rich (+) | Low_complexity | 510 |
| region is 510 bp dow | C-rich (+) | Low_complexity | 510 |
| region is 613 bp upst | L1M5 (+) | LINE | 613 |
| region is 958 bp dow | MIRb (-) | SINE | 958 |
| region is 558 bp dow | Charlie4z (-) | DNA | 558 |
| region is 359 bp dow | AT_rich (+) | Low_complexity | 359 |
| region is 791 bp dow | AT_rich (+) | Low_complexity | 791 |
| region is 972 bp upst | MER81 (+) | DNA | 972 |
| region is 458 bp dow | MER11A (-) | DNA | 458 |
| region is 458 bp dow | MER11A (-) | DNA | 458 |
| region is 454 bp upst | MER5A1 (+) | DNA | 454 |
| region is 412 bp dow | L2a (+) | LINE | 412 |
| region is 929 bp upst | (A)n (+) | Simple_repeat | 929 |
| region is 464 bp dow | AluSx4 (-) | SINE | 464 |
| region is 399 bp dow | MIR (+) | SINE | 399 |
| region is 363 bp dow | (T)n (+) | Simple_repeat | 363 |
| region is 507 bp dow | (T)n (+) | Simple_repeat | 507 |
| region is 739 bp dow | FLAM_C (-) | SINE | 739 |
| region is 221 bp dow | GC_rich (+) | Low_complexity | 221 |
| region is 368 bp dow | AluJo (-) | SINE | 368 |
| region is 132 bp dow | (T)n (+) | Simple_repeat | 132 |
| region is 387 bp dow | AluYa5 (-) | SINE | 387 |
| region is 772 bp dow | MIRc (-) | SINE | 772 |
| region is 310 bp dow | L1M5 (-) | LINE | 310 |
| region is 240 bp dow | L4 (-) | LTR | 240 |

| | | | | | | | | | | |
|---|---|---|---|---|---|---|---|---|---|---|
| FR048796 | intron of SUDS3 (+) | SUDS3 (+) | 0 | region is 5 bp from C | COSM3811176 | 5 | region is 392 bp upst | MIRb (+) | SINE | 392 |
| FR127343 | contained within UN( | UNC119B (+) | 0 | region is 380 bp from | COSM1316993 | 380 | region is 2241 bp ups | AluY (-) | SINE | 2241 |
| FR123582 | contained within UN( | UNC119B (+) | 0 | region is 1317 bp fro | COSM1316993 | 1317 | region is 2520 bp dov | AluSq2 (-) | SINE | 2520 |
| FR016000 | contained within UN( | UNC119B (+) | 0 | region is 2418 bp fro | COSM3356289 | 2418 | region is 393 bp dow | AluSq2 (-) | SINE | 393 |
| FR208892 | contained within VPS | VPS37B (-) | 0 | region is 890 bp from | COSM3688016 | 890 | region is 1696 bp ups | L4 (-) | LTR | 1696 |
| FR246461 | region is 24367 bp up | MICU2 (-) | 24367 | region is 25 bp from | COSM1322937 | 25 | region is 182 bp upst | HAL1 (-) | LINE | 182 |
| FR318724 | region is 200555 bp d | BASP1P1 (-) | 200555 | region is 507226 bp f | COSM175524 | 507226 | region is 117 bp upst | (CGG)n (+) | Simple_repeat | 117 |
| FR372499 | contained within WA | WASF3 (+) | 0 | region is 1335 bp fro | COSM432255 | 1335 | region is 1763 bp ups | MER3 (-) | DNA | 1763 |
| FR155758 | contained within TSC | TSC22D1 (-) | 0 | region is 36 bp from | COSM1158201 | 36 | region is 615 bp upst | GC_rich (+) | Low_complexity | 615 |
| FR326119 | contained within SN( | SNORA31 (-) | 0 | region is 1086 bp fro | COSM3793304 | 1086 | region is 523 bp dow | AluSx1 (-) | SINE | 523 |
| FR357791.1 | intron of ATP7B (-) | ATP7B (-) | 0 | region is 1713 bp fro | COSM1140403 | 1713 | region is 401 bp upst | MIR (-) | SINE | 401 |
| FR027884 | contained within MIR | MIR18A (+) | 0 | region is 48265 bp fr | COSM948883 | 48265 | region is 1737 bp dov | GC_rich (+) | Low_complexity | 1737 |
| FR135042 | contained within MIR | MIR18A (+) | 0 | region is 48264 bp fr | COSM948883 | 48264 | region is 1737 bp dov | GC_rich (+) | Low_complexity | 1737 |
| FR262980 | contained within MIR | MIR18A (+) | 0 | region is 48267 bp fr | COSM948883 | 48267 | region is 1737 bp dov | GC_rich (+) | Low_complexity | 1737 |
| FR042730 | contained within POT | POTEM (-) | 0 | region is 2137 bp fro | COSM1587271 | 2137 | region is 1701 bp ups | L1PA12 (-) | LINE | 1701 |
| FR319444 | intron of CHD8 (-), re | SNORD9 (-) | 0 | region is 254 bp from | COSM1649097 | 254 | region is 851 bp upst | MIRc (-) | SINE | 851 |
| FR143879 | contained within SN( | SNORD8 (-) | 0 | region is 421 bp from | COSM352513 | 421 | region is 496 bp upst | AluSx3 (-) | SINE | 496 |
| FR283482 | region is 1593 bp dov | MMP14 (+) | 1593 | region is 3208 bp fro | COSM131157 | 3208 | region overlaps with | AT_rich (+) | Low_complexity | 0 |
| FR134850 | contained within PPF | PPP1R3E (-) | 0 | region is 805 bp from | COSM1607516 | 805 | region is 89 bp upstr | GC_rich (+) | Low_complexity | 89 |
| FR227926 | contained within DN/ | DNAAF2 (-) | 0 | region is 30 bp from | COSM3793712 | 30 | region is 111 bp dow | GC_rich (+) | Low_complexity | 111 |
| FR056860 | contained within PNI | PNMA1 (-) | 0 | region is 168 bp from | COSM1678120 | 168 | region is 134 bp upst | MamGyp-int (-) | LTR | 134 |
| FR029743 | contained within GST | GSTZ1 (+) | 0 | region is 539 bp from | COSM3754126 | 539 | region is 109 bp dow | (CGGGG)n (+) | Simple_repeat | 109 |
| FR162794 | region is 16305 bp do | KCNK10 (-) | 16305 | region is 21743 bp fr | COSM1707658 | 21743 | region is 119 bp upst | L2a (+) | LINE | 119 |
| FR202470 | region is 16305 bp do | KCNK10 (-) | 16305 | region is 21743 bp fr | COSM1707658 | 21743 | region is 119 bp upst | L2a (+) | LINE | 119 |
| FR344447 | region is 16299 bp do | KCNK10 (-) | 16299 | region is 21737 bp fr | COSM1707658 | 21737 | region is 113 bp upst | L2a (+) | LINE | 113 |
| FR052968 | intron of MEG8 (+) | MEG8 (+) | 0 | region is 4902 bp fro | COSM4147820 | 4902 | contained within L1M | L1ME5 (+) | LINE | 0 |
| FR309811 | intron of MEG8 (+) | MEG8 (+) | 0 | region is 4902 bp fro | COSM4147820 | 4902 | contained within L1M | L1ME5 (+) | LINE | 0 |
| FR060041 | region is 2508 bp dov | SNORD113-2 (+) | 2508 | region is 36901 bp fr | COSM4147820 | 36901 | contained within L1M | L1ME5 (+) | LINE | 0 |
| FR353474 | region is 2508 bp dov | SNORD113-2 (+) | 2508 | region is 36901 bp fr | COSM4147820 | 36901 | contained within L1M | L1ME5 (+) | LINE | 0 |
| FR184142 | region is 2165 bp ups | SNORD113-9 (+) | 2165 | region is 50433 bp fr | COSM4147820 | 50433 | contained within L1M | L1ME5 (+) | LINE | 0 |
| FR298757 | region is 2166 bp ups | SNORD113-9 (+) | 2166 | region is 50433 bp fr | COSM4147820 | 50433 | contained within L1M | L1ME5 (+) | LINE | 0 |
| FR015426 | region is 2165 bp ups | SNORD113-9 (+) | 2165 | region is 50434 bp fr | COSM4147820 | 50434 | contained within L1M | L1ME5 (+) | LINE | 0 |
| FR071598 | region is 2165 bp ups | SNORD113-9 (+) | 2165 | region is 50434 bp fr | COSM4147820 | 50434 | contained within L1M | L1ME5 (+) | LINE | 0 |
| FR118379 | contained within SN( | SNORD113-9 (+) | 0 | region is 52632 bp fr | COSM4147820 | 52632 | region is 297 bp upst | MER3 (-) | DNA | 297 |
| FR028698 | contained within SN( | SNORD114-1 (+) | 0 | region is 56815 bp fr | COSM4147820 | 56815 | region is 751 bp upst | AT_rich (+) | Low_complexity | 751 |
| FR004819 | contained within SN( | SNORD114-1 (+) | 0 | region is 56850 bp fr | COSM4147820 | 56850 | region is 716 bp upst | AT_rich (+) | Low_complexity | 716 |
| FR301191 | contained within SN( | SNORD114-1 (+) | 0 | region is 56852 bp fr | COSM4147820 | 56852 | region is 717 bp upst | AT_rich (+) | Low_complexity | 717 |
| FR267761 | contained within SN( | SNORD114-3 (+) | 0 | region is 60331 bp fr | COSM4147820 | 60331 | region is 4 bp downs | AluSx (+) | SINE | 4 |
| FR007639 | contained within SN( | SNORD114-22 (+) | 0 | region is 89908 bp fr | COSM4147820 | 89908 | region is 2111 bp dov | AluSx1 (-) | SINE | 2111 |
| FR245310 | contained within SN( | SNORD114-23 (+) | 0 | region is 90893 bp fr | COSM4147820 | 90893 | region is 1127 bp dov | AluSx1 (-) | SINE | 1127 |
| FR249679 | intron of EIF5 (+), reg | SNORA28 (+) | 0 | region is 482 bp from | COSM3419646 | 482 | region is 109 bp upst | Kanga1a (-) | DNA | 109 |
| FR165524 | contained within GOI | GOLGA8CP (+) | 0 | region is 8373 bp fro | COSM3401625 | 8373 | region is 267 bp dow | (CA)n (+) | Simple_repeat | 267 |
| FR165524_dup1 | contained within GOI | GOLGA8DP (-) | 0 | region is 26962 bp fr | COSM1477935 | 26962 | region is 267 bp upst | (TG)n (+) | Simple_repeat | 267 |
| FR165524_dup4 | contained within GOI | GOLGA8S (+) | 0 | region is 105 bp from | COSM1587769 | 105 | region is 267 bp dow | (CA)n (+) | Simple_repeat | 267 |
| FR019019 | contained within PW/ | SNORD107 (+) | 0 | region is 3574 bp fro | COSM226306 | 3574 | region is 428 bp upst | AluSx (-) | SINE | 428 |
| FR233520.1 | intron of HERC2 (-) | HERC2 (-) | 0 | region is 162 bp from | COSM1608222 | 162 | region is 120 bp upst | AluSx3 (+) | SINE | 120 |
| FR165524_dup7 | contained within GOI | GOLGA8F (-) | 0 | region is 1828 bp fro | COSM2083625 | 1828 | region is 266 bp upst | (TG)n (+) | Simple_repeat | 266 |
| FR165524_dup8 | contained within GOI | GOLGA8F (-) | 0 | region is 1828 bp fro | COSM2083625 | 1828 | region is 266 bp upst | (TG)n (+) | Simple_repeat | 266 |
| FR111929 | contained within BMI | BMF (-) | 0 | region is 3290 bp fro | COSM279179 | 3290 | region is 848 bp upst | MIRb (-) | SINE | 848 |
| FR079998 | contained within SEF | SERF2 (+) | 0 | region is 14 bp from | COSM341042 | 14 | region is 1476 bp ups | AluSx3 (-) | SINE | 1476 |
| FR267209 | intron of CYP19A1 (-) | CYP19A1 (-) | 0 | region is 44171 bp fr | COSM701072 | 44171 | region is 927 bp upst | L2c (+) | LINE | 927 |
| FR324253 | intron of CYP19A1 (-) | CYP19A1 (-) | 0 | region is 46311 bp fr | COSM701072 | 46311 | region is 882 bp upst | L2c (+) | LINE | 882 |

| | | | | | | | | | | |
|---|---|---|---|---|---|---|---|---|---|---|
| FR338565.1 | region is 157441 bp u | ONECUT1 (-) | 157441 | region is 148544 bp f | COSM1218448 | 148544 | region is 594 bp upst | MIRb (+) | SINE | 594 |
| FR395322 | region is 57684 bp up | C2CD4B (-) | 57684 | region is 24325 bp fr | COSM86506 | 24325 | region is 788 bp upst | AluSq (+) | SINE | 788 |
| FR148815 | region is 63847 bp do | MIR8067 (-) | 63847 | region is 6511 bp fro | COSM86506 | 6511 | region is 1556 bp ups | AT_rich (+) | Low_complexity | 1556 |
| FR109932 | region is 54247 bp do | MIR8067 (-) | 54247 | region is 3057 bp fro | COSM86506 | 3057 | region is 241 bp upst | MIRc (-) | SINE | 241 |
| FR070884 | region is 54003 bp do | MIR8067 (-) | 54003 | region is 3302 bp fro | COSM86506 | 3302 | region is 486 bp upst | MIRc (-) | SINE | 486 |
| FR121747 | region is 54004 bp do | MIR8067 (-) | 54004 | region is 3302 bp fro | COSM86506 | 3302 | region is 486 bp upst | MIRc (-) | SINE | 486 |
| FR389121 | region is 46890 bp do | MIR8067 (-) | 46890 | region is 10417 bp fr | COSM86506 | 10417 | region is 39 bp down | MIR3 (-) | SINE | 39 |
| FR293713 | contained within SNO | SNORD16 (-) | 0 | region is 68 bp from | COSM194143 | 68 | region is 223 bp upst | L1MA4 (-) | LINE | 223 |
| FR123248 | contained within MES | MESDC1 (+) | 0 | region is 427 bp from | COSM3754503 | 427 | region is 65 bp upstr | GC_rich (+) | Low_complexity | 65 |
| FR376350 | intron of GOLGA6L10 | UBE2Q2P2 (+) | 0 | region is 23457 bp fr | COSM2152248 | 23457 | region is 492 bp dow | Charlie19a (-) | DNA | 492 |
| FR232277 | intron of GOLGA6L10 | GOLGA6L10 (-) | 0 | region is 34169 bp fr | COSM3932180 | 34169 | region is 107 bp upst | (CAGGG)n (+) | Simple_repeat | 107 |
| FR232277_dup1 | intron of GOLGA6L10 | GOLGA6L9 (+) | 0 | region is 36386 bp fr | COSM3999635 | 36386 | region is 107 bp dow | (CCCTG)n (+) | Simple_repeat | 107 |
| FR232277_dup2 | intron of GOLGA6L10 | GOLGA6L9 (+) | 0 | region is 36386 bp fr | COSM3999635 | 36386 | region is 107 bp dow | (CCCTG)n (+) | Simple_repeat | 107 |
| FR232277_dup3 | intron of GOLGA6L10 | GOLGA6L9 (+) | 0 | region is 36386 bp fr | COSM3999635 | 36386 | region is 107 bp dow | (CCCTG)n (+) | Simple_repeat | 107 |
| FR376350_dup1 | intron of GOLGA6L17 | UBE2Q2P2 (+) | 0 | region is 25997 bp fr | COSM3401960 | 25997 | region is 493 bp dow | Charlie19a (-) | DNA | 493 |
| FR232277_dup4 | intron of GOLGA6L17 | GOLGA6L17P (+) | 0 | region is 35622 bp fr | COSM1600347 | 35622 | region is 107 bp upst | (CAGGG)n (+) | Simple_repeat | 107 |
| FR145670_dup2 | contained within GOI | LOC727751 (-) | 0 | region is 37132 bp fr | COSM1600347 | 37132 | region overlaps with | MIR3 (+) | SINE | 0 |
| FR327232 | contained within FSD | SCARNA15 (+) | 0 | region is 3330 bp fro | COSM3706768 | 3330 | region is 428 bp dow | AT_rich (+) | Low_complexity | 428 |
| FR232277_dup5 | intron of LOC440300 | LOC440300 (+) | 0 | region is 42068 bp fr | COSM4128490 | 42068 | region is 815 bp dow | MIRc (-) | SINE | 815 |
| FR145670_dup3 | contained within GOI | GOLGA2P7 (-) | 0 | region is 40564 bp fr | COSM4128490 | 40564 | region is 155 bp dow | AluJb (+) | SINE | 155 |
| FR232277_dup6 | region is 21238 bp up | LOC103171574 (+) | 21238 | region is 46092 bp fr | COSM4128493 | 46092 | region is 572 bp upst | L2c (+) | LINE | 572 |
| FR232277_dup7 | region is 2137 bp dov | LOC642423 (-) | 2137 | region is 65893 bp fr | COSM87727 | 65893 | region is 107 bp upst | (CAGGG)n (+) | Simple_repeat | 107 |
| FR145670_dup5 | region is 628 bp dow | LOC642423 (-) | 628 | region is 67403 bp fr | COSM87727 | 67403 | region overlaps with | MIR3 (+) | SINE | 0 |
| FR357791.2 | intron of AKAP13 (+) | AKAP13 (+) | 0 | region is 70491 bp fr | COSM309019 | 70491 | region is 698 bp dow | L2c (-) | LINE | 698 |
| FR221589 | contained within FAN | POLG (-) | 0 | region is 73 bp from | COSM2149526 | 73 | region is 1422 bp ups | AluJr (-) | SINE | 1422 |
| FR196011 | contained within FAN | POLG (-) | 0 | region is 74 bp from | COSM2149526 | 74 | region is 1423 bp ups | AluJr (-) | SINE | 1423 |
| FR270038 | contained within LINC | LINC00930 (-) | 0 | region is 58359 bp fr | COSM1206192 | 58359 | region is 240 bp dow | L2 (+) | LINE | 240 |
| FR161697 | region is 8077 bp ups | LINC00930 (-) | 8077 | region is 49850 bp fr | COSM1206192 | 49850 | region is 784 bp dow | L2b (+) | LINE | 784 |
| FR207571 | region is 326417 bp u | RGMA (-) | 326417 | region is 209374 bp f | COSM3771935 | 209374 | region is 982 bp upst | LTR1 (-) | LTR | 982 |
| FR323470 | intron of SPATA8-AS | SPATA8-AS1 (-) | 0 | region is 9572 bp fro | COSM1518243 | 9572 | region is 139 bp upst | AT_rich (+) | Low_complexity | 139 |
| FR102859 | intron of SPATA8-AS | SPATA8-AS1 (-) | 0 | region is 3824 bp fro | COSM1518243 | 3824 | region is 1541 bp ups | L2c (+) | LINE | 1541 |
| FR004109 | contained within IGF | IGF1R (+) | 0 | region is 4999 bp fro | COSM1254644 | 4999 | region is 2130 bp dov | (TTTTG)n (+) | Simple_repeat | 2130 |
| FR106433 | contained within SYN | SYNM (+) | 0 | region is 930 bp from | COSM223817 | 930 | region is 1909 bp dov | MER81 (-) | DNA | 1909 |
| FR371811 | contained within DNM | DNM1P46 (-) | 0 | region is 184 bp from | COSM959503 | 184 | region is 432 bp dow | 7SLRNA (+) | srpRNA | 432 |
| FR046594 | contained within DNM | DNM1P46 (-) | 0 | region is 185 bp from | COSM959503 | 185 | region is 433 bp dow | 7SLRNA (+) | srpRNA | 433 |
| FR021331_dup16 | region is 32356 bp up | TARSL2 (-) | 32356 | region is 3994 bp fro | COSM470436 | 3994 | region is 4622 bp dov | AluJo (+) | SINE | 4622 |
| FR021331_dup12 | region is 36624 bp up | TARSL2 (-) | 36624 | region is 8262 bp fro | COSM470436 | 8262 | region is 5225 bp ups | (CTCTG)n (+) | Simple_repeat | 5225 |
| FR021331_dup9 | region is 37494 bp up | TARSL2 (-) | 37494 | region is 9132 bp fro | COSM470436 | 9132 | region is 4355 bp ups | (CTCTG)n (+) | Simple_repeat | 4355 |
| FR191737_dup9 | region is 37496 bp up | TARSL2 (-) | 37496 | region is 9134 bp fro | COSM470436 | 9134 | region is 4353 bp ups | (CTCTG)n (+) | Simple_repeat | 4353 |
| FR027151 | region is 388 bp upst | C16orf13 (-) | 388 | region is 587 bp from | COSM1324377 | 587 | region is 360 bp upst | (CGGGG)n (+) | Simple_repeat | 360 |
| FR319465 | region is 408 bp upst | C16orf13 (-) | 408 | region is 607 bp from | COSM1324377 | 607 | region is 335 bp upst | (CGGGG)n (+) | Simple_repeat | 335 |
| FR089006 | region is 411 bp upst | C16orf13 (-) | 411 | region is 610 bp from | COSM1324377 | 610 | region is 334 bp upst | (CGGGG)n (+) | Simple_repeat | 334 |
| FR106521 | region is 412 bp upst | C16orf13 (-) | 412 | region is 611 bp from | COSM1324377 | 611 | region is 335 bp upst | (CGGGG)n (+) | Simple_repeat | 335 |
| FR348209 | region is 412 bp upst | C16orf13 (-) | 412 | region is 611 bp from | COSM1324377 | 611 | region is 334 bp upst | (CGGGG)n (+) | Simple_repeat | 334 |
| FR167602 | contained within PDP | PDPK1 (+) | 0 | region is 1968 bp fro | COSM3957387 | 1968 | region is 1026 bp dov | L1ME4a (-) | LINE | 1026 |
| FR389105 | contained within PDP | PDPK1 (+) | 0 | region is 3473 bp fro | COSM3957387 | 3473 | region is 61 bp upstr | L1ME4a (-) | LINE | 61 |
| FR243247 | contained within PDP | PDPK1 (+) | 0 | region is 4550 bp fro | COSM3957387 | 4550 | region is 1138 bp ups | L1ME4a (-) | LINE | 1138 |
| FR389105_dup1 | region is 2817 bp ups | KCTD5 (+) | 2817 | region is 2976 bp fro | COSM3772175 | 2976 | region is 61 bp upstr | L1ME4a (-) | LINE | 61 |
| FR243247_dup1 | region is 1738 bp ups | KCTD5 (+) | 1738 | region is 1897 bp fro | COSM3772175 | 1897 | region is 560 bp dow | AluSg7 (-) | SINE | 560 |
| FR341404 | contained within UBN | UBN1 (+) | 0 | region is 22 bp from | COSM3754909 | 22 | region is 663 bp dow | FAM (+) | SINE | 663 |
| FR003837_dup3 | contained within LOC | PKD1P6 (-) | 0 | region is 33 bp from | COSM3944377 | 33 | region is 61 bp upstr | AluSg7 (+) | SINE | 61 |
| FR003837_dup4 | contained within NPI | NPIPA5 (-) | 0 | COSM1135762, COSM | COSM1135763 | 0 | region is 61 bp upstr | AluSg7 (+) | SINE | 61 |

| | | | | | | | | | | | |
|---|---|---|---|---|---|---|---|---|---|---|---|
| FR003837_dup8 | contained within NPI | NPIPA8 (-) | | 0 | region is 93376 bp fr | COSM1733682 | 93376 | region is 61 bp upstr | AluSg7 (+) | SINE | 61 |
| FR003837_dup7 | region is 14751 bp do | MIR6770-2 (-) | | 14751 | region is 53240 bp fr | COSM1733682 | 53240 | region is 61 bp upstr | AluSg7 (+) | SINE | 61 |
| FR003837_dup9 | contained within NPI | NPIPB3 (-) | | 0 | region is 6323 bp fro | COSM1723200 | 6323 | region is 61 bp upstr | AluSg7 (+) | SINE | 61 |
| FR003837_dup10 | region is 24555 bp up | RRN3P1 (-) | | 24555 | region is 196 bp from | COSM434877 | 196 | region is 61 bp upstr | AluSg7 (+) | SINE | 61 |
| FR073001.2 | region is 65660 bp do | C16orf82 (+) | | 65660 | region is 69250 bp fr | COSM3817639 | 69250 | region is 190 bp upst | AluSq2 (-) | SINE | 190 |
| FR055135 | contained within EIF3 | EIF3C (-) | | 0 | region is 1473 bp fro | COSM1470851 | 1473 | region is 171 bp dow | MER5A (+) | DNA | 171 |
| FR055135_dup1 | contained within EIF3 | EIF3CL (+) | | 0 | region is 27 bp from | COSM3361711 | 27 | region is 194 bp dow | MER5A (-) | DNA | 194 |
| FR003837_dup13 | region is 25225 bp do | SNX29P2 (+) | | 25225 | COSM1588376, COSI | COSM1588377 | 0 | region is 61 bp upstr | AluSg7 (+) | SINE | 61 |
| FR003837_dup14 | region is 24232 bp up | LOC613038 (-) | | 24232 | region is 4615 bp fro | COSM132799 | 4615 | region is 61 bp upstr | AluSg7 (+) | SINE | 61 |
| FR258764 | contained within MAZ | MAZ (+) | | 0 | COSM1147662, COSI | COSM120942 | 0 | region is 365 bp dow | (CCG)n (+) | Simple_repeat | 365 |
| FR258764_dup1 | contained within MAZ | MAZ (+) | | 0 | COSM1147662, COSI | COSM120942 | 0 | region is 365 bp dow | (CCG)n (+) | Simple_repeat | 365 |
| FR242750 | contained within MAZ | MAZ (+) | | 0 | COSM1478766, COSI | COSM435073 | 0 | region is 913 bp dow | (CCG)n (+) | Simple_repeat | 913 |
| FR127556 | region is 141 bp upst | KCTD13 (-) | | 141 | region is 423 bp from | COSM1478776 | 423 | region is 204 bp upst | FRAM (+) | SINE | 204 |
| FR003837_dup15 | contained within LOC | LOC613037 (-) | | 0 | region is 6367 bp fro | COSM1318597 | 6367 | region is 61 bp upstr | AluSg7 (+) | SINE | 61 |
| FR314551 | contained within CD2 | CD2BP2 (-) | | 0 | region is 308 bp from | COSM3948464 | 308 | region is 505 bp dow | L2c (+) | LINE | 505 |
| FR156205 | region is 2429 bp dov | PRR14 (+) | | 2429 | region is 696 bp from | COSM4128984 | 696 | region is 362 bp dow | G-rich (+) | Low_complexity | 362 |
| FR293228 | contained within CMT | CMTM4 (-) | | 0 | region is 1145 bp fro | COSM1629949 | 1145 | region is 4126 bp ups | Helitron3Na_Mam (+) | RC | 4126 |
| FR309048 | contained within HAS | HAS3 (+) | | 0 | COSM266655, COSM | COSM460482 | 0 | region is 1832 bp ups | G-rich (+) | Low_complexity | 1832 |
| FR066386 | region is 959 bp dow | VPS4A (+) | | 959 | region is 1702 bp fro | COSM435623 | 1702 | region is 60 bp down | MER102b (+) | DNA | 60 |
| FR255141 | contained within SNO | SNORD71 (-) | | 0 | region is 379 bp from | COSM435724 | 379 | region is 372 bp dow | MIRc (+) | SINE | 372 |
| FR071609 | contained within IST1 | IST1 (+) | | 0 | region is 8 bp from C | COSM3701120 | 8 | region is 175 bp upst | MIR (+) | SINE | 175 |
| FR087630 | contained within KAR | KARS (-) | | 0 | COSM1141004, COSI | COSM1520018 | 0 | region is 134 bp dow | AluSp (-) | SINE | 134 |
| FR003121 | region is 41715 bp up | CNTNAP4 (+) | | 41715 | region is 42112 bp fr | COSM1201709 | 42112 | region is 128 bp upst | C-rich (+) | Low_complexity | 128 |
| FR341694 | contained within SNO | SNORD68 (+) | | 0 | region is 91 bp from | COSM137077 | 91 | region is 545 bp dow | GC_rich (+) | Low_complexity | 545 |
| FR384374 | contained within FAN | FANCA (-) | | 0 | COSM3818742, COSI | COSM3818743 | 0 | region is 265 bp dow | AluSg (+) | SINE | 265 |
| FR154596 | contained within SMY | SMYD4 (-) | | 0 | region is 719 bp from | COSM116351 | 719 | region is 32 bp down | AluY (-) | SINE | 32 |
| FR363585 | contained within HIC | HIC1 (+) | | 0 | region is 8 bp from C | COSM1381412 | 8 | region is 345 bp upst | (CGG)n (+) | Simple_repeat | 345 |
| FR359148 | contained within SNO | SNORD91A (-) | | 0 | region is 210 bp from | COSM3421345 | 210 | region is 66 bp upstr | AluSp (-) | SINE | 66 |
| FR321895 | contained within SNO | SNORD91A (-) | | 0 | region is 210 bp from | COSM3421345 | 210 | region is 67 bp upstr | AluSp (-) | SINE | 67 |
| FR126778 | contained within CYB | CYB5D2 (+) | | 0 | region is 10 bp from | COSM4130092 | 10 | region is 355 bp dow | L2b (-) | LINE | 355 |
| FR095339 | intron of USP6 (+) | USP6 (+) | | 0 | region is 170 bp from | COSM1384525 | 170 | region is 611 bp dow | L1MC3 (+) | LINE | 611 |
| FR401591 | contained within ELP | ELP5 (+) | | 0 | COSM1246582, COSI | COSM1246583 | 0 | region is 483 bp upst | L2b (+) | LINE | 483 |
| FR092448 | contained within ELP | ELP5 (+) | | 0 | COSM1246582, COSI | COSM1246583 | 0 | region is 483 bp upst | L2b (+) | LINE | 483 |
| FR068891 | contained within MIR | MIR744 (+) | | 0 | contained within COS | COSM26115 | 0 | region is 1011 bp ups | MER5B (-) | DNA | 1011 |
| FR091055 | contained within MIR | MIR744 (+) | | 0 | contained within COS | COSM26115 | 0 | region is 1011 bp ups | MER5B (-) | DNA | 1011 |
| FR167222 | region is 325477 bp u | CDRT7 (+) | | 325477 | region is 64693 bp fr | COSM975680 | 64693 | region is 338 bp dow | L1MC5 (-) | LINE | 338 |
| FR008939 | intron of FLII (-) | FLII (-) | | 0 | region is 152 bp from | COSM436155 | 152 | region is 42 bp down | MIR (+) | SINE | 42 |
| FR095339_dup1 | region is 8670 bp dov | EVPLL (+) | | 8670 | region is 10074 bp fr | COSM160848 | 10074 | region is 632 bp dow | L1MC3 (+) | LINE | 632 |
| FR210393 | contained within SPE | SPECC1 (+) | | 0 | COSM3755332, COSI | COSM3755332 | 0 | region is 1030 bp ups | MER5B (-) | DNA | 1030 |
| FR095339_dup2 | region is 33598 bp up | CDRT15L2 (+) | | 33598 | region is 33626 bp fr | COSM4129690 | 33626 | region is 632 bp dow | L1MC3 (-) | LINE | 632 |
| FR369781_dup2 | contained within CCD | LOC440416 (+) | | 0 | region is 109 bp from | COSM1247468 | 109 | region is 697 bp upst | AluSz (+) | SINE | 697 |
| FR227782.1 | region is 974 bp upst | MTRNR2L1 (+) | | 974 | region is 195163 bp f | COSM436192 | 195163 | region is 1882 bp ups | LSU-rRNA_Hsa (+) | rRNA | 1882 |
| FR398935_dup1 | region is 7913 bp dov | KRT18P55 (-) | | 7913 | region is 51173 bp fr | COSM129789 | 51173 | region is 129 bp dow | HERV-Fc1_LTR1 (+) | LTR | 129 |
| FR016773 | contained within SNO | SNORD42B (+) | | 0 | region is 92 bp from | COSM3356897 | 92 | region is 456 bp upst | AluSp (+) | SINE | 456 |
| FR248000 | contained within SNO | SNORD4A (+) | | 0 | region is 87 bp from | COSM977000 | 87 | region is 144 bp upst | AluSc (-) | SINE | 144 |
| FR108660 | contained within GIT | GIT1 (-) | | 0 | region is 26 bp from | COSM118923 | 26 | region is 2057 bp ups | L2b (+) | LINE | 2057 |
| FR233520.2 | region is 58408 bp do | DPRXP4 (+) | | 58408 | region is 35232 bp fr | COSM3819228 | 35232 | region is 128 bp dow | AT_rich (+) | Low_complexity | 128 |
| FR095339_dup4 | region is 24695 bp do | CCL3L3 (-) | | 24695 | region is 209 bp from | COSM436421 | 209 | region is 773 bp dow | L1MC3 (-) | LINE | 773 |
| FR095339_dup6 | region is 24695 bp do | CCL3L3 (-) | | 24695 | region is 209 bp from | COSM436421 | 209 | region is 773 bp dow | L1MC3 (-) | LINE | 773 |
| FR095339_dup7 | region is 24695 bp do | CCL3L3 (-) | | 24695 | region is 209 bp from | COSM436421 | 209 | region is 773 bp dow | L1MC3 (-) | LINE | 773 |
| FR095339_dup3 | intron of LOC101060 | TBC1D3F (-) | | 0 | region is 346 bp from | COSM328391 | 346 | region is 630 bp dow | L1MD3 (-) | LINE | 630 |
| FR095339_dup5 | intron of LOC101060 | TBC1D3F (-) | | 0 | region is 346 bp from | COSM328391 | 346 | region is 630 bp dow | L1MD3 (-) | LINE | 630 |

| | | | |
|---|---|---|---|
| FR095339_dup9 | intron of LOC101060 | TBC1D3F (-) | 0 |
| FR095339_dup8 | intron of TBC1D3B (- | TBC1D3B (-) | 0 |
| FR095339_dup10 | intron of LOC101060 | LOC102723859 (+) | 0 |
| FR095339_dup11 | intron of LOC101060 | LOC102723859 (+) | 0 |
| FR095339_dup12 | intron of LOC101060 | TBC1D3F (-) | 0 |
| FR095339_dup13 | intron of LOC101060 | TBC1D3F (-) | 0 |
| FR159523 | region is 378 bp upst | SOCS7 (+) | 378 |
| FR332156 | contained within SN | SNORA21 (-) | 0 |
| FR086093.1 | region is 2457 bp ups | SNORA21 (-) | 2457 |
| FR083318 | contained within TM | TMEM99 (+) | 0 |
| FR083863 | contained within TM | TMEM99 (+) | 0 |
| FR172647 | contained within FA | TUBG1 (+) | 0 |
| FR049269 | contained within ATX | ATXN7L3 (-) | 0 |
| FR250713 | region is 14399 bp do | LOC644172 (+) | 14399 |
| FR246461.1 | intron of NSF (+) | NSF (+) | 0 |
| FR377799 | contained within CO | COL1A1 (-) | 0 |
| FR106597 | contained within LR | LRRC59 (-) | 0 |
| FR166990 | contained within LR | LRRC59 (-) | 0 |
| FR279668 | contained within SR | SRSF1 (-) | 0 |
| FR095339_dup14 | intron of TBC1D3P1- | TBC1D3P1-DHX40P1 | 0 |
| FR095339_dup15 | intron of TBC1D3P2 ( | TBC1D3P2 (-) | 0 |
| FR309588 | contained within DC | DCAF7 (+) | 0 |
| FR246220_dup1 | contained within LR | LRRC37A3 (-) | 0 |
| FR251847 | contained within LIN | LINC00674 (+) | 0 |
| FR251847_dup1 | contained within LIN | LINC00674 (+) | 0 |
| FR252722 | contained within LIN | LINC00674 (+) | 0 |
| FR252722_dup1 | contained within LIN | LINC00674 (+) | 0 |
| FR131044 | contained within SN | SNORD1B (+) | 0 |
| FR114004 | contained within SN | SNORD1B (+) | 0 |
| FR132358 | contained within SN | SNORD1A (+) | 0 |
| FR321576 | contained within PG | PGS1 (+) | 0 |
| FR295879 | contained within RN | RNF213 (+) | 0 |
| FR290641 | contained within RN | LOC100294362 (-) | 0 |
| FR061877_dup1 | contained within AC | ACTG1 (-) | 0 |
| FR077664 | contained within AC | ACTG1 (-) | 0 |
| FR082509 | contained within NP | NPLOC4 (-) | 0 |
| FR271504 | contained within MA | MAFG (-) | 0 |
| FR271504_dup1 | contained within MA | MAFG (-) | 0 |
| FR324396 | contained within LIN | LINC00526 (-) | 0 |
| FR003837.1 | region is 15307 bp do | SLC35G4 (+) | 15307 |
| FR242079 | region is 8563 bp do | GAREM (-) | 8563 |
| FR310293 | contained within SN | SNORD58C (-) | 0 |
| FR132879 | contained within SN | SNORD58A (-) | 0 |
| FR064000 | contained within RP | SNORD58B (-) | 0 |
| FR293687 | contained within ZN | ZNF532 (+) | 0 |
| FR376353 | contained within PQ | PQLC1 (-) | 0 |
| FR384068_dup12 | region is 11153 bp do | PPAP2C (-) | 11153 |
| FR384068_dup10 | region is 11083 bp do | PPAP2C (-) | 11083 |
| FR384068_dup8 | region is 11017 bp do | PPAP2C (-) | 11017 |
| FR384068_dup5 | region is 10951 bp do | PPAP2C (-) | 10951 |
| FR384068_dup13 | region is 10885 bp do | PPAP2C (-) | 10885 |
| FR384068_dup17 | region is 10815 bp do | PPAP2C (-) | 10815 |

| | | |
|---|---|---|
| region is 46974 bp fr | COSM4129892 | 46974 |
| region is 1645 bp fro | COSM2154776 | 1645 |
| region is 1084 bp fro | COSM249130 | 1084 |
| region is 1084 bp fro | COSM249130 | 1084 |
| region is 346 bp from | COSM328393 | 346 |
| region is 346 bp from | COSM328393 | 346 |
| region is 756 bp from | COSM3749397 | 756 |
| region is 35 bp from | COSM1224209 | 35 |
| region is 2417 bp fro | COSM1736218 | 2417 |
| COSM560867 | COSM560867 | 0 |
| COSM560867 | COSM560867 | 0 |
| COSM706295 | COSM706295 | 0 |
| region is 1 bp from C | COSM1194021 | 1 |
| region is 35849 bp fr | COSM328398 | 35849 |
| region is 4789 bp fro | COSM3421642 | 4789 |
| region is 11 bp from | COSM122272 | 11 |
| region is 928 bp from | COSM1750139 | 928 |
| region is 715 bp from | COSM1750139 | 715 |
| region is 233 bp from | COSM981712 | 233 |
| region is 177 bp from | COSM3958607 | 177 |
| region is 101014 bp f | COSM1479830 | 101014 |
| region is 2764 bp fro | COSM349408 | 2764 |
| region is 24 bp from | COSM3421797 | 24 |
| region is 80083 bp fr | COSM473256 | 80083 |
| region is 80083 bp fr | COSM473256 | 80083 |
| region is 80170 bp fr | COSM473256 | 80170 |
| region is 80170 bp fr | COSM473256 | 80170 |
| region is 4976 bp fro | COSM1750343 | 4976 |
| region is 4933 bp fro | COSM1750343 | 4933 |
| region is 4452 bp fro | COSM1750343 | 4452 |
| COSM301134, COSM | COSM985083 | 0 |
| region is 63 bp from | COSM437736 | 63 |
| region is 7 bp from C | COSM1522829 | 7 |
| region is 85 bp from | COSM3720700 | 85 |
| COSM1130115, COSM | COSM388712 | 0 |
| region is 3 bp from C | COSM1259391 | 3 |
| region is 2281 bp fro | COSM473548 | 2281 |
| region is 2281 bp fro | COSM473548 | 2281 |
| region is 40940 bp fr | COSM988991 | 40940 |
| COSM986564 | COSM986564 | 0 |
| region is 12969 bp fr | COSM1661776 | 12969 |
| region is 88 bp from | COSM1480356 | 88 |
| region is 197 bp from | COSM1589494 | 197 |
| region is 151 bp from | COSM1153244 | 151 |
| region is 35 bp from | COSM213747 | 35 |
| COSM474064, COSM | COSM990115 | 0 |
| region is 11504 bp fr | COSM1392190 | 11504 |
| region is 11434 bp fr | COSM1392190 | 11434 |
| region is 11368 bp fr | COSM1392190 | 11368 |
| region is 11302 bp fr | COSM1392190 | 11302 |
| region is 11236 bp fr | COSM1392190 | 11236 |
| region is 11166 bp fr | COSM1392190 | 11166 |

| | | | |
|---|---|---|---|
| region is 768 bp dow | L1MC3 (-) | LINE | 768 |
| region is 614 bp dow | L1MD3 (-) | LINE | 614 |
| region is 773 bp dow | L1MC3 (+) | LINE | 773 |
| region is 773 bp dow | L1MC3 (+) | LINE | 773 |
| region is 773 bp dow | L1MC3 (-) | LINE | 773 |
| region is 773 bp dow | L1MC3 (-) | LINE | 773 |
| region is 2 bp upstre | GC_rich (+) | Low_complexity | 2 |
| region is 555 bp dow | AluSx (+) | SINE | 555 |
| region is 245 bp dow | AluSp (-) | SINE | 245 |
| region is 254 bp upst | (TTTTG)n (+) | Simple_repeat | 254 |
| region is 255 bp upst | (TTTTG)n (+) | Simple_repeat | 255 |
| region is 1416 bp do | MIR (-) | SINE | 1416 |
| region is 841 bp dow | AluSg (+) | SINE | 841 |
| region is 398 bp dow | AluSp (-) | SINE | 398 |
| region is 1878 bp ups | L2b (+) | LINE | 1878 |
| region is 2057 bp ups | L1MC4 (+) | LINE | 2057 |
| region is 250 bp upst | MIR3 (+) | SINE | 250 |
| region is 37 bp upstr | MIR3 (+) | SINE | 37 |
| region is 2136 bp do | AluSz6 (+) | SINE | 2136 |
| region is 752 bp dow | L1MC3 (-) | LINE | 752 |
| region is 772 bp dow | L1MC3 (-) | LINE | 772 |
| region is 167 bp upst | AluY (+) | SINE | 167 |
| region is 368 bp upst | AluJb (-) | SINE | 368 |
| region is 352 bp upst | AluJb (-) | SINE | 352 |
| region is 352 bp upst | AluJb (-) | SINE | 352 |
| region is 439 bp upst | AluJb (-) | SINE | 439 |
| region is 439 bp upst | AluJb (-) | SINE | 439 |
| region is 119 bp dow | AluSp (+) | SINE | 119 |
| region is 166 bp dow | AluSp (+) | SINE | 166 |
| region is 53 bp upstr | T-rich (+) | Low_complexity | 53 |
| region is 316 bp dow | L2b (+) | LINE | 316 |
| region is 591 bp dow | AluY (-) | SINE | 591 |
| region is 658 bp dow | AluSx3 (+) | SINE | 658 |
| region is 1587 bp ups | L1M5 (-) | LINE | 1587 |
| region is 1123 bp ups | GC_rich (+) | Low_complexity | 1123 |
| region is 58 bp upstr | AluY (+) | SINE | 58 |
| region is 1250 bp ups | (T)n (+) | Simple_repeat | 1250 |
| region is 1250 bp ups | (T)n (+) | Simple_repeat | 1250 |
| region is 1068 bp do | L2c (-) | LINE | 1068 |
| region is 61 bp upstr | AluSg7 (+) | SINE | 61 |
| region is 204 bp dow | MLT1J (+) | LTR | 204 |
| region is 99 bp upstr | AluY (-) | SINE | 99 |
| region is 1222 bp do | Charlie4a (+) | DNA | 1222 |
| region is 1523 bp ups | L2 (+) | LINE | 1523 |
| region is 1577 bp ups | L2c (-) | LINE | 1577 |
| region is 121 bp upst | G-rich (+) | Low_complexity | 121 |
| region is 40 bp down | L1MC3 (+) | LINE | 40 |
| region is 110 bp dow | L1MC3 (+) | LINE | 110 |
| region is 176 bp dow | L1MC3 (+) | LINE | 176 |
| region is 242 bp dow | L1MC3 (+) | LINE | 242 |
| region is 308 bp dow | L1MC3 (+) | LINE | 308 |
| region is 378 bp dow | L1MC3 (+) | LINE | 378 |

| | | | | | | | | | | | |
|---|---|---|---|---|---|---|---|---|---|---|---|
| FR384068 | region is 10745 bp do | PPAP2C (-) | 10745 | region is 11096 bp fr | COSM1392190 | 11096 | | region is 448 bp dow | L1MC3 (+) | LINE | 448 |
| FR384068_dup6 | region is 10679 bp do | PPAP2C (-) | 10679 | region is 11030 bp fr | COSM1392190 | 11030 | | region is 514 bp dow | L1MC3 (+) | LINE | 514 |
| FR384068_dup15 | region is 10613 bp do | PPAP2C (-) | 10613 | region is 10964 bp fr | COSM1392190 | 10964 | | region is 580 bp dow | L1MC3 (+) | LINE | 580 |
| FR384068_dup4 | region is 10547 bp do | PPAP2C (-) | 10547 | region is 10898 bp fr | COSM1392190 | 10898 | | region is 549 bp upst | L1MC3 (+) | LINE | 549 |
| FR384068_dup1 | region is 10477 bp do | PPAP2C (-) | 10477 | region is 10828 bp fr | COSM1392190 | 10828 | | region is 479 bp upst | L1MC3 (+) | LINE | 479 |
| FR384068_dup9 | region is 10411 bp do | PPAP2C (-) | 10411 | region is 10762 bp fr | COSM1392190 | 10762 | | region is 413 bp upst | L1MC3 (+) | LINE | 413 |
| FR384068_dup2 | region is 10341 bp do | PPAP2C (-) | 10341 | region is 10692 bp fr | COSM1392190 | 10692 | | region is 343 bp upst | L1MC3 (+) | LINE | 343 |
| FR384068_dup11 | region is 10271 bp do | PPAP2C (-) | 10271 | region is 10622 bp fr | COSM1392190 | 10622 | | region is 273 bp upst | L1MC3 (+) | LINE | 273 |
| FR384068_dup14 | region is 10201 bp do | PPAP2C (-) | 10201 | region is 10552 bp fr | COSM1392190 | 10552 | | region is 203 bp upst | L1MC3 (+) | LINE | 203 |
| FR384068_dup3 | region is 10131 bp do | PPAP2C (-) | 10131 | region is 10482 bp fr | COSM1392190 | 10482 | | region is 133 bp upst | L1MC3 (+) | LINE | 133 |
| FR384068_dup16 | region is 10065 bp do | PPAP2C (-) | 10065 | region is 10416 bp fr | COSM1392190 | 10416 | | region is 67 bp upstr | L1MC3 (+) | LINE | 67 |
| FR384068_dup7 | region is 9995 bp dov | PPAP2C (-) | 9995 | region is 10346 bp fr | COSM1392190 | 10346 | | region overlaps with | L1MC3 (+) | LINE | 0 |
| FR045157 | contained within CIR | CIRBP (+) | 0 | region is 664 bp from | COSM991345 | 664 | | region is 623 bp upst | L3 (-) | LTR | 623 |
| FR355504 | contained within MRI | MRPL54 (+) | 0 | COSM1750799, COSI | COSM2155187 | 0 | | region is 137 bp upst | MIR (+) | SINE | 137 |
| FR302680 | contained within SNO | SNORD41 (-) | 0 | region is 115 bp from | COSM1390627 | 115 | | region is 335 bp dow | L2b (+) | LINE | 335 |
| FR019089 | contained within SNO | SNORD41 (-) | 0 | region is 114 bp from | COSM1390627 | 114 | | region is 336 bp dow | L2b (+) | LINE | 336 |
| FR374098 | contained within TNF | TNPO2 (-) | 0 | COSM3796645, COSI | COSM3796646 | 0 | | region is 511 bp upst | AluSc8 (-) | SINE | 511 |
| FR016548 | contained within TNF | TNPO2 (-) | 0 | COSM1194653, COSI | COSM1194654 | 0 | | region is 367 bp dow | AluJr (-) | SINE | 367 |
| FR204697 | intron of LINC00661 ( | LINC00661 (+) | 0 | region is 18807 bp fr | COSM51648 | 18807 | | region is 231 bp dow | HSAT5 (-) | Satellite | 231 |
| FR255519 | contained within LIN | LINC00661 (+) | 0 | region is 13613 bp fr | COSM51648 | 13613 | | region is 263 bp upst | AluSx (+) | SINE | 263 |
| FR129496.1 | contained within RPL | RPL18A (+) | 0 | region is 2 bp from C | COSM1750723 | 2 | | region is 1224 bp dov | AluJb (-) | SINE | 1224 |
| FR362622 | contained within MA | MAST3 (+) | 0 | COSM3796800, COSI | COSM3796801 | 0 | | region is 231 bp dow | LTR55 (-) | LTR | 231 |
| FR316592 | contained within JUN | JUND (-) | 0 | region is 55 bp from | COSM711120 | 55 | | region is 23 bp down | (CGG)n (+) | Simple_repeat | 23 |
| FR372571 | contained within RGS | RGS9BP (+) | 0 | region is 87 bp from | COSM1392623 | 87 | | region is 808 bp dow | GC_rich (+) | Low_complexity | 808 |
| FR233167 | region is 1960 bp dov | LOC400685 (-) | 1960 | region is 44615 bp fr | COSM3959923 | 44615 | | region is 233 bp upst | THE1D (+) | LTR | 233 |
| FR162939 | region is 1243 bp dov | LOC400685 (-) | 1243 | region is 45332 bp fr | COSM3959923 | 45332 | | region is 86 bp down | THE1D (+) | LTR | 86 |
| FR315126 | region is 329 bp dow | LOC400685 (-) | 329 | region is 46245 bp fr | COSM3959923 | 46245 | | region is 999 bp dow | THE1D (+) | LTR | 999 |
| FR151159 | region is 324 bp dow | LOC400685 (-) | 324 | region is 46251 bp fr | COSM3959923 | 46251 | | region is 1005 bp dov | THE1D (+) | LTR | 1005 |
| FR252245 | region is 322 bp dow | LOC400685 (-) | 322 | region is 46252 bp fr | COSM3959923 | 46252 | | region is 1006 bp dov | THE1D (+) | LTR | 1006 |
| FR305534 | region is 324 bp dow | LOC400685 (-) | 324 | region is 46252 bp fr | COSM3959923 | 46252 | | region is 1006 bp dov | THE1D (+) | LTR | 1006 |
| FR010002 | region is 322 bp dow | LOC400685 (-) | 322 | region is 46253 bp fr | COSM3959923 | 46253 | | region is 1007 bp dov | THE1D (+) | LTR | 1007 |
| FR036138 | region is 1351 bp dov | LINC00904 (-) | 1351 | region is 41187 bp fr | COSM1392763 | 41187 | | region is 1830 bp ups | (TA)n (+) | Simple_repeat | 1830 |
| FR038115 | contained within SPI | SPINT2 (+) | 0 | region is 8710 bp fro | COSM4140595 | 8710 | | region is 776 bp dow | L2a (-) | LINE | 776 |
| FR157961 | contained within RPS | RPS19 (+) | 0 | region is 7 bp from C | COSM1712375 | 7 | | region is 386 bp dow | GC_rich (+) | Low_complexity | 386 |
| FR325599 | contained within LYP | LYPD5 (-) | 0 | COSM1129805 | COSM1129805 | 0 | | region is 115 bp upst | CT-rich (+) | Low_complexity | 115 |
| FR097339 | region is 10032 bp up | AP2S1 (-) | 10032 | region is 14888 bp fr | COSM418154 | 14888 | | region is 26 bp down | C-rich (+) | Low_complexity | 26 |
| FR222326_dup1 | contained within SNA | SNAR-A13 (+) | 0 | region is 21520 bp fr | COSM998825 | 21520 | | region is 111 bp dow | AluSx1 (-) | SINE | 111 |
| FR222326_dup2 | contained within SNA | SNAR-A2 (+) | 0 | region is 32271 bp fr | COSM998825 | 32271 | | region is 111 bp dow | AluSx1 (-) | SINE | 111 |
| FR222326_dup3 | contained within SNA | SNAR-A9 (+) | 0 | region is 37621 bp fr | COSM998825 | 37621 | | region is 110 bp dow | AluSx1 (-) | SINE | 110 |
| FR222326_dup4 | contained within SNA | SNAR-A2 (+) | 0 | region is 42464 bp fr | COSM1394976 | 42464 | | region is 111 bp dow | AluSx1 (-) | SINE | 111 |
| FR222326 | contained within SNA | SNAR-A13 (+) | 0 | region is 31723 bp fr | COSM1394976 | 31723 | | region is 111 bp dow | AluSx1 (-) | SINE | 111 |
| FR118220 | contained within RPL | RPL18 (-) | 0 | COSM4132226 | COSM4132226 | 0 | | region is 670 bp dow | (CACAG)n (+) | Simple_repeat | 670 |
| FR003121.1 | contained within RPL | RPL18 (-) | 0 | region is 11 bp from | COSM474992 | 11 | | region is 519 bp dow | AluJo (-) | SINE | 519 |
| FR297613 | contained within SNO | SNORD34 (+) | 0 | region is 49 bp from | COSM999311 | 49 | | region is 1426 bp dov | AluJb (+) | SINE | 1426 |
| FR349959 | contained within SNO | SNORD34 (+) | 0 | region is 49 bp from | COSM999311 | 49 | | region is 1426 bp dov | AluJb (+) | SINE | 1426 |
| FR018916 | contained within SNO | SNORD35A (+) | 0 | region is 83 bp from | COSM3835382 | 83 | | region is 1694 bp dov | AluJb (+) | SINE | 1694 |
| FR222326_dup8 | contained within SNA | SNAR-A9 (-) | 0 | region is 45486 bp fr | COSM475066 | 45486 | | region is 110 bp dow | AluSx1 (+) | SINE | 110 |
| FR222326_dup6 | contained within SNA | SNAR-A9 (-) | 0 | region is 50823 bp fr | COSM475066 | 50823 | | region is 110 bp dow | AluSx1 (+) | SINE | 110 |
| FR222326_dup11 | contained within SNA | SNAR-A9 (-) | 0 | region is 51726 bp fr | COSM3707308 | 51726 | | region is 110 bp dow | AluSx1 (+) | SINE | 110 |
| FR222326_dup12 | contained within SNA | SNAR-A9 (-) | 0 | region is 48662 bp fr | COSM3707308 | 48662 | | region is 110 bp dow | AluSx1 (+) | SINE | 110 |
| FR222326_dup7 | contained within SNA | SNAR-A9 (-) | 0 | region is 45603 bp fr | COSM3707308 | 45603 | | region is 110 bp dow | AluSx1 (+) | SINE | 110 |
| FR222326_dup10 | contained within SNA | SNAR-A9 (-) | 0 | region is 40249 bp fr | COSM3707308 | 40249 | | region is 110 bp dow | AluSx1 (+) | SINE | 110 |

| | | | |
|---|---|---|---|
| FR222326_dup13 | contained within SNA | SNAR-A9 (-) | 0 |
| FR222326_dup9 | contained within SNA | SNAR-A9 (-) | 0 |
| FR222326_dup5 | contained within SNA | SNAR-A9 (-) | 0 |
| FR149623 | contained within RPL | RPL28 (+) | 0 |
| FR326725 | contained within ADC | ADCY3 (-) | 0 |
| FR217789.1 | contained within FTH | FTH1P3 (-) | 0 |
| FR027151.1 | region is 362 bp upst | TIA1 (-) | 362 |
| FR319465.1 | region is 382 bp upst | TIA1 (-) | 382 |
| FR106521.1 | region is 386 bp upst | TIA1 (-) | 386 |
| FR348209.1 | region is 386 bp upst | TIA1 (-) | 386 |
| FR225984 | region is 345 bp upst | WDR54 (+) | 345 |
| FR026913 | contained within SNC | SNORD89 (-) | 0 |
| FR102402.3 | region is 103273 bp d | ST6GAL2 (-) | 103273 |
| FR073001.3 | region is 103249 bp d | ST6GAL2 (-) | 103249 |
| FR338565.2 | region is 103090 bp d | ST6GAL2 (-) | 103090 |
| FR086093.2 | region is 19801 bp do | FLJ42351 (-) | 19801 |
| FR266905 | region is 791069 bp u | DDX18 (+) | 791069 |
| FR048331_dup8 | region is 8447 bp dov | POTEJ (+) | 8447 |
| FR048331_dup7 | region is 10609 bp up | CYP4F30P (+) | 10609 |
| FR048331_dup6 | region is 8802 bp ups | CYP4F30P (+) | 8802 |
| FR294567 | contained within PLE | PLEKHB2 (+) | 0 |
| FR048331_dup9 | region is 2014 bp ups | LOC440910 (+) | 2014 |
| FR319486.1 | intron of KIF5C (+) | KIF5C (+) | 0 |
| FR227782.2 | intron of KIF5C (+) | KIF5C (+) | 0 |
| FR082269 | intron of KIF5C (+) | KIF5C (+) | 0 |
| FR390022 | region is 9664 bp ups | NR4A2 (-) | 9664 |
| FR295924 | intron of B3GALT1 (+ | B3GALT1 (+) | 0 |
| FR026803.1 | intron of SCHLAP1 (+ | SCHLAP1 (+) | 0 |
| FR217789_dup1 | intron of SCHLAP1 (+ | SCHLAP1 (+) | 0 |
| FR024428 | region is 402 bp upst | BMPR2 (+) | 402 |
| FR344651 | contained within CYF | CYP20A1 (+) | 0 |
| FR116115 | contained within ABI | ABI2 (+) | 0 |
| FR246367 | contained within SNC | SNORD51 (+) | 0 |
| FR376350.1 | region is 6310 bp ups | PLEKHM3 (-) | 6310 |
| FR236999 | contained within SNC | SNORD20 (-) | 0 |
| FR075316 | contained within SNC | SNORD82 (-) | 0 |
| FR390460 | contained within SCA | SCARNA6 (+) | 0 |
| FR357447 | intron of DGKD (+) | DGKD (+) | 0 |
| FR236081 | contained within AGA | AGAP1 (+) | 0 |
| FR159422 | contained within ASE | ASB1 (+) | 0 |
| FR284952 | intron of LINC01107 ( | LINC01107 (-) | 0 |
| FR018977 | contained within STK | STK35 (+) | 0 |
| FR287130 | contained within STK | STK35 (+) | 0 |
| FR270731 | contained within SNC | SNORD57 (+) | 0 |
| FR301184 | contained within LOC | LOC643406 (+) | 0 |
| FR185905 | contained within SNC | SNORD17 (-) | 0 |
| FR290142 | contained within SNC | SNORD17 (-) | 0 |
| FR344796 | contained within SNC | SNORD17 (-) | 0 |
| FR352491 | contained within SNC | SNORD17 (-) | 0 |
| FR233520.3 | region is 37002 bp do | PAX1 (+) | 37002 |
| FR218180 | contained within LIN | LINC00657 (-) | 0 |
| FR391445 | contained within DNA | DNAJC5 (+) | 0 |

| | | |
|---|---|---|
| region is 34897 bp fr | COSM3707308 | 34897 |
| region is 29543 bp fr | COSM3707308 | 29543 |
| region is 24215 bp fr | COSM3707308 | 24215 |
| COSM1396578 | COSM1396578 | 0 |
| COSM242851 | COSM242851 | 0 |
| region is 5749 bp fro | COSM418755 | 5749 |
| region is 604 bp from | COSM443144 | 604 |
| region is 624 bp from | COSM443144 | 624 |
| region is 628 bp from | COSM443144 | 628 |
| region is 628 bp from | COSM443144 | 628 |
| region is 907 bp from | COSM212599 | 907 |
| region is 3626 bp fro | COSM475666 | 3626 |
| region is 108355 bp f | COSM1526977 | 108355 |
| region is 108331 bp f | COSM1526977 | 108331 |
| region is 108172 bp f | COSM1526977 | 108172 |
| region is 24804 bp fr | COSM1193870 | 24804 |
| region is 791175 bp f | COSM4133028 | 791175 |
| region is 8945 bp fro | COSM3961136 | 8945 |
| region is 11875 bp fr | COSM3961136 | 11875 |
| region is 13682 bp fr | COSM3961136 | 13682 |
| region is 7 bp from C | COSM1720060 | 7 |
| region is 9881 bp fro | COSM4133114 | 9881 |
| region is 40337 bp fr | COSM261674 | 40337 |
| region is 40337 bp fr | COSM261674 | 40337 |
| region is 40305 bp fr | COSM261674 | 40305 |
| region is 9941 bp fro | COSM4133186 | 9941 |
| region is 27475 bp fr | COSM3961506 | 27475 |
| region is 109125 bp f | COSM81875 | 109125 |
| region is 109123 bp f | COSM81875 | 109123 |
| region is 1565 bp fro | COSM442125 | 1565 |
| COSM1015114 | COSM1015114 | 0 |
| region is 1629 bp fro | COSM1326512 | 1629 |
| region is 123 bp from | COSM3962240 | 123 |
| region is 30234 bp fr | COSM719547 | 30234 |
| region is 107 bp from | COSM1641864 | 107 |
| region is 91 bp from | COSM1017751 | 91 |
| region is 917 bp from | COSM1017972 | 917 |
| region is 513 bp from | COSM210415 | 513 |
| COSM1018230, COSI | COSM1018230 | 0 |
| region is 2362 bp fro | COSM1018598 | 2362 |
| region is 98089 bp fr | COSM1018598 | 98089 |
| region is 29016 bp fr | COSM1533233 | 29016 |
| region is 29034 bp fr | COSM1533233 | 29034 |
| region is 52 bp from | COSM3963429 | 52 |
| region is 73696 bp fr | COSM3953078 | 73696 |
| region is 5654 bp fro | COSM1410562 | 5654 |
| region is 5655 bp fro | COSM1410562 | 5655 |
| region is 5653 bp fro | COSM1410562 | 5653 |
| region is 5657 bp fro | COSM1410562 | 5657 |
| region is 40691 bp fr | COSM1260987 | 40691 |
| region is 19477 bp fr | COSM1411481 | 19477 |
| region is 2735 bp fro | COSM4134814 | 2735 |

| | | | |
|---|---|---|---|
| region is 110 bp dow | AluSx1 (+) | SINE | 110 |
| region is 110 bp dow | AluSx1 (+) | SINE | 110 |
| region is 110 bp dow | AluSx1 (+) | SINE | 110 |
| region is 405 bp upst | MIR (+) | SINE | 405 |
| region is 2379 bp dov | AT_rich (+) | Low_complexity | 2379 |
| region is 285 bp upst | AluSx (-) | SINE | 285 |
| region is 449 bp upst | AluSx1 (+) | SINE | 449 |
| region is 424 bp upst | AluSx1 (+) | SINE | 424 |
| region is 424 bp upst | AluSx1 (+) | SINE | 424 |
| region is 423 bp upst | AluSx1 (+) | SINE | 423 |
| region is 303 bp upst | AluSx (-) | SINE | 303 |
| region is 422 bp dow | AluJb (-) | SINE | 422 |
| region is 454 bp dow | L1MC5 (-) | LINE | 454 |
| region is 430 bp dow | L1MC5 (-) | LINE | 430 |
| region is 271 bp dow | L1MC5 (-) | LINE | 271 |
| region is 226 bp upst | ERVL-B4-int (-) | LTR | 226 |
| region is 231 bp from | tRNA-Leu-TTA(m) (+) | tRNA | 231 |
| region is 989 bp dow | L1P3 (-) | LINE | 989 |
| region is 1871 bp ups | L1P3 (-) | LINE | 1871 |
| region is 357 bp dow | MST-int (-) | LTR | 357 |
| region is 79 bp down | FAM (+) | SINE | 79 |
| region is 995 bp dow | MST-int (-) | LTR | 995 |
| region is 334 bp upst | MIRc (+) | SINE | 334 |
| region is 334 bp upst | MIRc (+) | SINE | 334 |
| region is 302 bp upst | MIRc (+) | SINE | 302 |
| region is 45 bp down | GC_rich (+) | Low_complexity | 45 |
| region is 333 bp upst | AT_rich (+) | Low_complexity | 333 |
| region is 254 bp dow | L1MA3 (+) | LINE | 254 |
| region is 259 bp dow | L1MA3 (+) | LINE | 259 |
| region is 195 bp upst | GC_rich (+) | Low_complexity | 195 |
| region is 415 bp upst | MER5A (-) | DNA | 415 |
| region is 482 bp upst | AluSc (+) | SINE | 482 |
| region is 70 bp down | MIR (+) | SINE | 70 |
| region is 171 bp dow | L1MA9 (-) | LINE | 171 |
| region is 128 bp dow | L4 (+) | LTR | 128 |
| region is 292 bp upst | (CAT)n (+) | Simple_repeat | 292 |
| region is 338 bp upst | AluJr (-) | SINE | 338 |
| region is 326 bp upst | AluSx1 (-) | SINE | 326 |
| region is 310 bp upst | (TATATG)n (+) | Simple_repeat | 310 |
| region is 762 bp upst | (TTTA)n (+) | Simple_repeat | 762 |
| region is 6926 bp ups | L1M6 (+) | LINE | 6926 |
| region is 2317 bp dov | MIR (-) | SINE | 2317 |
| region is 2299 bp dov | MIR (-) | SINE | 2299 |
| region is 2986 bp ups | AluSg (-) | SINE | 2986 |
| region is 1060 bp ups | MIR (+) | SINE | 1060 |
| region is 209 bp upst | AluSg (-) | SINE | 209 |
| region is 209 bp upst | AluSg (-) | SINE | 209 |
| region is 209 bp upst | AluSg (-) | SINE | 209 |
| region is 209 bp upst | AluSg (-) | SINE | 209 |
| region is 125 bp upst | (A)n (+) | Simple_repeat | 125 |
| region is 1110 bp dov | L2a (-) | LINE | 1110 |
| region is 1892 bp ups | AluJb (+) | SINE | 1892 |

| | | | | | | | | | | |
|---|---|---|---|---|---|---|---|---|---|---|
| FR271551 | contained within MIR | MIRLET7C (+) | 0 | region is 468519 bp f | COSM3770900 | 468519 | region is 418 bp upst | L2c (+) | LINE | 418 |
| FR161625 | region is 5626 bp do | PDE9A (+) | 5626 | region is 5843 bp fro | COSM1190366 | 5843 | region is 112 bp upst | LTR38B (-) | LTR | 112 |
| FR298744 | contained within PDX | PDXK (+) | 0 | region is 229 bp from | COSM119945 | 229 | region is 1737 bp ups | (T)n (+) | Simple_repeat | 1737 |
| FR127490 | contained within TSP | TSPEAR (-) | 0 | region is 1223 bp fro | COSM1172754 | 1223 | region is 280 bp upst | MER4C (-) | DNA | 280 |
| FR042730.2 | region is 202 bp dow | POTEH (-) | 202 | region is 2155 bp fro | COSM1031769 | 2155 | region is 1698 bp ups | L1PA12 (-) | LINE | 1698 |
| FR296333 | contained within DG( | MIR1306 (+) | 0 | COSM1032175, COSI | COSM122562 | 0 | region is 652 bp dow | AT_rich (+) | Low_complexity | 652 |
| FR403738 | contained within MIF | MIF (+) | 0 | region is 65 bp from | COSM419625 | 65 | region is 547 bp upst | GC_rich (+) | Low_complexity | 547 |
| FR115804 | contained within MIF | MIF (+) | 0 | region is 5 bp from C | COSM1616282 | 5 | region is 170 bp upst | GC_rich (+) | Low_complexity | 170 |
| FR104116 | contained within MIF | MIF (+) | 0 | region is 4 bp from C | COSM1616282 | 4 | region is 134 bp upst | GC_rich (+) | Low_complexity | 134 |
| FR140045 | contained within MIF | MIF-AS1 (-) | 0 | region is 435 bp from | COSM1327303 | 435 | region is 424 bp upst | AluYb8 (+) | SINE | 424 |
| FR234237 | intron of MIF-AS1 (-) | MIF-AS1 (-) | 0 | region is 698 bp from | COSM1327303 | 698 | region is 161 bp upst | AluYb8 (+) | SINE | 161 |
| FR159092 | contained within GU( | GUCD1 (-) | 0 | region is 15 bp from | COSM1032728 | 15 | region is 84 bp upstr | L2c (+) | LINE | 84 |
| FR225244 | contained within NF2 | NF2 (+) | 0 | region is 1930 bp fro | COSM327309 | 1930 | region is 89 bp down | AluSx (-) | SINE | 89 |
| FR233520.4 | intron of RBFOX2 (-) | RBFOX2 (-) | 0 | region is 1754 bp fro | COSM1033795 | 1754 | region is 160 bp dow | AT_rich (+) | Low_complexity | 160 |
| FR234424 | contained within LO( | LOC100506271 (-) | 0 | region is 19900 bp fr | COSM3842629 | 19900 | region is 161 bp upst | L2a (-) | LINE | 161 |
| FR312499 | intron of ELFN2 (-) | ELFN2 (-) | 0 | region is 17336 bp fr | COSM3842629 | 17336 | region is 35 bp upstr | MER46C (+) | DNA | 35 |
| FR049370 | contained within ELF | ELFN2 (-) | 0 | region is 3952 bp fro | COSM3842629 | 3952 | region is 219 bp dow | (CCCA)n (+) | Simple_repeat | 219 |
| FR319300 | contained within SN( | SNORD83B (-) | 0 | region is 158 bp from | COSM478973 | 158 | region is 2051 bp ups | AluSp (+) | SINE | 2051 |
| FR205579 | contained within SN( | SNORD83B (-) | 0 | region is 159 bp from | COSM478973 | 159 | region is 2051 bp ups | AluSp (+) | SINE | 2051 |
| FR213383 | contained within SN( | SNORD83B (-) | 0 | region is 160 bp from | COSM478973 | 160 | region is 2051 bp ups | AluSp (+) | SINE | 2051 |
| FR237180 | contained within SN( | SNORD83B (-) | 0 | region is 161 bp from | COSM478973 | 161 | region is 2051 bp ups | AluSp (+) | SINE | 2051 |
| FR218523 | contained within SN( | SNORD43 (-) | 0 | region is 517 bp from | COSM33359 | 517 | region is 1246 bp do | AluSx3 (-) | SINE | 1246 |
| FR042730_dup1 | region is 17619 bp up | MIR1281 (+) | 17619 | region is 18128 bp fr | COSM445040 | 18128 | region is 424 bp dow | AluJb (-) | SINE | 424 |
| FR102284 | region is 1065 bp do | CCDC134 (+) | 1065 | region is 1582 bp fro | COSM1199831 | 1582 | region is 17 bp down | MER51B (-) | DNA | 17 |
| FR092610 | contained within ZBE | ZBED4 (+) | 0 | region is 30 bp from | COSM316635 | 30 | region is 1212 bp do | FRAM (+) | SINE | 1212 |
| FR135938 | contained within ZBE | ZBED4 (+) | 0 | region is 9 bp from C | COSM726773 | 9 | region is 2017 bp do | FRAM (+) | SINE | 2017 |
| FR054799 | contained within VGL | VGLL4 (-) | 0 | COSM1270210, COSI | COSM79096 | 0 | region is 607 bp upst | MIR3 (-) | SINE | 607 |
| FR030135 | contained within RPL | RPL32 (-) | 0 | region is 12 bp from | COSM79274 | 12 | region is 355 bp dow | MER4E (+) | DNA | 355 |
| FR072519 | contained within RPL | RPL32 (-) | 0 | COSM3408212 | COSM3408212 | 0 | region is 330 bp dow | MIRc (+) | SINE | 330 |
| FR355867 | region is 22249 bp up | SNORA7A (-) | 22249 | region is 11888 bp fr | COSM1593327 | 11888 | region is 281 bp upst | AluY (+) | SINE | 281 |
| FR238825 | contained within IQS | IQSEC1 (-) | 0 | region is 2900 bp fro | COSM4157255 | 2900 | region is 2010 bp ups | L2 (-) | LINE | 2010 |
| FR239801 | contained within IQS | IQSEC1 (-) | 0 | region is 2225 bp fro | COSM4157255 | 2225 | region is 1768 bp ups | (TA)n (+) | Simple_repeat | 1768 |
| FR270274 | contained within NR2 | NR2C2 (+) | 0 | region is 4041 bp fro | COSM181530 | 4041 | region is 10 bp upstr | (TATATG)n (+) | Simple_repeat | 10 |
| FR161271 | region is 719 bp upst | UBP1 (-) | 719 | region is 1255 bp fro | COSM480035 | 1255 | region is 408 bp upst | AluSz (+) | SINE | 408 |
| FR373605 | region is 2378 bp do | RPL14 (+) | 2378 | region is 2604 bp fro | COSM1044561 | 2604 | region is 141 bp upst | MIRb (+) | SINE | 141 |
| FR069557 | contained within MIR | MIR138-1 (+) | 0 | region is 127809 bp f | COSM1617613 | 127809 | region is 211 bp upst | Charlie4z (-) | DNA | 211 |
| FR214430 | intron of ZKSCAN7 (- | ZKSCAN7 (+) | 0 | region is 9434 bp fro | COSM297215 | 9434 | region is 209 bp upst | GC_rich (+) | Low_complexity | 209 |
| FR284409 | contained within RBI | RBM15B (+) | 0 | COSM584355 | COSM584355 | 0 | region is 258 bp dow | (CCG)n (+) | Simple_repeat | 258 |
| FR090658 | contained within WD | WDR82 (-) | 0 | region is 2 bp from C | COSM1046846 | 2 | region is 59 bp down | AluSq (+) | SINE | 59 |
| FR035643.1 | region is 196606 bp u | EPHA6 (+) | 196606 | region is 701 bp from | COSM3767596 | 701 | region is 801 bp upst | AluSx1 (+) | SINE | 801 |
| FR167222.1 | intron of PCNP (+) | PCNP (+) | 0 | region is 221 bp from | COSM3786797 | 221 | region is 83 bp down | AluSc (-) | SINE | 83 |
| FR274844 | contained within AD( | ADCY5 (-) | 0 | region is 5 bp from C | COSM350722 | 5 | region is 1141 bp ups | L2b (+) | LINE | 1141 |
| FR026803.2 | intron of RAB7A (+) | RAB7A (+) | 0 | region is 30812 bp fr | COSM1536499 | 30812 | region is 678 bp upst | L1MC4 (-) | LINE | 678 |
| FR217789.2 | intron of RAB7A (+) | RAB7A (+) | 0 | region is 30810 bp fr | COSM1536499 | 30810 | region is 683 bp upst | L1MC4 (-) | LINE | 683 |
| FR002120 | region is 12468 bp do | LOC653712 (-) | 12468 | region is 30706 bp fr | COSM1536496 | 30706 | region is 1677 bp do | L1MA7 (-) | LINE | 1677 |
| FR372135 | contained within SN( | SNORA58 (-) | 0 | region is 7936 bp fro | COSM2149652 | 7936 | region is 143 bp upst | AT_rich (+) | Low_complexity | 143 |
| FR035643_dup1 | intron of PPM1L (+) | PPM1L (+) | 0 | region is 14053 bp fr | COSM1536852 | 14053 | region overlaps with | L2b (+) | LINE | 0 |
| FR162734 | region is 48362 bp up | MIR3138 (-) | 48362 | region is 10403 bp fr | COSM1618199 | 10403 | region is 293 bp dow | LTR5_Hs (-) | LTR | 293 |
| FR007558 | region is 57769 bp up | MIR3138 (-) | 57769 | region is 19810 bp fr | COSM1618199 | 19810 | region is 1234 bp do | L1MCa (-) | LINE | 1234 |
| FR138193 | region is 61464 bp up | MIR3138 (-) | 61464 | region is 23505 bp fr | COSM1618199 | 23505 | region is 1317 bp ups | L1MB5 (-) | LINE | 1317 |
| FR266905.1 | region is 448249 bp d | LOC101929199 (+) | 448249 | region is 707506 bp f | COSM164648 | 707506 | region is 54 bp upstr | THE1B (-) | LTR | 54 |
| FR376350.2 | contained within RPL | RPL9 (-) | 0 | region is 41 bp from | COSM263446 | 41 | region is 905 bp dow | FLAM_C (+) | SINE | 905 |

| | | | | | | | | | | |
|---|---|---|---|---|---|---|---|---|---|---|
| FR252137 | intron of AFF1 (+) | AFF1 (+) | 0 | region is 75003 bp fr | COSM1058562 | 75003 | region is 424 bp dow | (T)n (+) | Simple_repeat | 424 |
| FR266905_dup1 | intron of SNCA (-) | SNCA (-) | 0 | region is 2662 bp fro | COSM1541094 | 2662 | region is 70 bp upstr | AluSc (+) | SINE | 70 |
| FR188833.2 | contained within LIN | LINC01061 (-) | 0 | region is 4747 bp fro | COSM3669230 | 4747 | region is 1007 bp do | MER1A (-) | DNA | 1007 |
| FR174266 | region overlaps with | LRAT (+) | 0 | region is 3477 bp fro | COSM3825438 | 3477 | region is 532 bp dow | MIRb (+) | SINE | 532 |
| FR289069 | region overlaps with | LRAT (+) | 0 | region is 3477 bp fro | COSM3825438 | 3477 | region is 532 bp dow | MIRb (+) | SINE | 532 |
| FR107946 | contained within SN | SNORD123 (+) | 0 | region is 80275 bp fr | COSM169015 | 80275 | region is 383 bp dow | MIRc (+) | SINE | 383 |
| FR322380 | contained within SN | SNORD123 (+) | 0 | region is 80275 bp fr | COSM169015 | 80275 | region is 384 bp dow | MIRc (+) | SINE | 384 |
| FR066510 | region is 12600 bp u | OTULIN (+) | 12600 | COSM1659593 | COSM1659593 | 0 | region is 558 bp dow | L1MA4 (+) | LINE | 558 |
| FR102402.4 | region is 11697 bp u | OTULIN (+) | 11697 | region is 11 bp from | COSM449054 | 11 | region is 414 bp dow | Charlie2b (-) | DNA | 414 |
| FR008494 | intron of C5orf34 (-) | C5orf34 (-) | 0 | region is 596 bp from | COSM3828114 | 596 | region is 1384 bp do | AluJo (-) | SINE | 1384 |
| FR233520.5 | intron of IL6ST (-) | IL6ST (-) | 0 | region is 2186 bp fro | COSM3776763 | 2186 | region is 128 bp ups | AT_rich (+) | Low_complexity | 128 |
| FR105053 | contained within LO | LOC644936 (-) | 0 | region is 20259 bp fr | COSM593291 | 20259 | region is 580 bp ups | SVA_D (+) | Other | 580 |
| FR163199 | contained within MIR | MIR3607 (+) | 0 | region is 1139 bp fro | COSM335695 | 1139 | region is 275 bp dow | MER20 (+) | DNA | 275 |
| FR043670 | intron of KIAA0825 (- | KIAA0825 (-) | 0 | region is 32360 bp fr | COSM1620655 | 32360 | region is 1427 bp do | BLACKJACK (-) | DNA | 1427 |
| FR003121.2 | region is 235619 bp | FTMT (+) | 235619 | region is 235630 bp f | COSM735927 | 235630 | region is 193 bp dow | AT_rich (+) | Low_complexity | 193 |
| FR008494_dup1 | region is 106338 bp | C5orf63 (-) | 106338 | region is 120836 bp f | COSM1186822 | 120836 | region is 108 bp dow | L1M2 (-) | LINE | 108 |
| FR361473 | intron of CDC42SE2 ( | CDC42SE2 (+) | 0 | region is 20754 bp fr | COSM1200379 | 20754 | region is 122 bp ups | AluSx (+) | SINE | 122 |
| FR004361 | contained within UQ | UQCRQ (+) | 0 | region is 58 bp from | COSM1060779 | 58 | region is 191 bp dow | L1MB7 (+) | LINE | 191 |
| FR338747 | intron of PCBD2 (+) | PCBD2 (+) | 0 | region is 16065 bp fr | COSM1541902 | 16065 | region is 1994 bp ups | AluY (+) | SINE | 1994 |
| FR380286 | contained within SN | SNORD63 (-) | 0 | region is 514 bp from | COSM204775 | 514 | region is 198 bp ups | AluY (-) | SINE | 198 |
| FR148093 | region is 14750 bp u | LOC101929696 (-) | 14750 | region is 48462 bp fr | COSM1130929 | 48462 | region is 124 bp dow | MIR (-) | SINE | 124 |
| FR157967 | intron of FAM153B (+ | FAM153B (+) | 0 | region is 990 bp from | COSM3827712 | 990 | region is 523 bp dow | AluSp (+) | SINE | 523 |
| FR289805 | contained within FAF | FAF2 (+) | 0 | region is 9 bp from C | COSM1496043 | 9 | region is 208 bp dow | FLAM_A (+) | SINE | 208 |
| FR157967_dup2 | intron of FAM153C (+ | FAM153C (+) | 0 | region is 983 bp from | COSM482599 | 983 | region is 136 bp ups | MIRb (-) | SINE | 136 |
| FR032701 | contained within SQ | SQSTM1 (+) | 0 | region is 6 bp from C | COSM3827841 | 6 | region is 333 bp dow | L2 (+) | LINE | 333 |
| FR352363 | contained within DSF | DSP (+) | 0 | region is 18 bp from | COSM1547434 | 18 | region is 1696 bp do | (TA)n (+) | Simple_repeat | 1696 |
| FR328400 | contained within DSF | DSP (+) | 0 | COSM2156615 | COSM2156615 | 0 | region is 2149 bp do | (TA)n (+) | Simple_repeat | 2149 |
| FR348796 | region is 19297 bp u | RNF144B (+) | 19297 | region is 31552 bp fr | COSM1744277 | 31552 | region is 631 bp ups | (CTG)n (+) | Simple_repeat | 631 |
| FR274916 | contained within C6o | C6orf62 (-) | 0 | region is 78 bp from | COSM450974 | 78 | region is 219 bp ups | AT_rich (+) | Low_complexity | 219 |
| FR306048 | contained within HIS | HIST1H2AC (+) | 0 | COSM3722038, COSI | COSM3722038 | 0 | region is 885 bp ups | MER101 (-) | DNA | 885 |
| FR205670 | contained within HIS | HIST1H1E (+) | 0 | COSM1311866 | COSM1311866 | 0 | region is 836 bp dow | AluY (-) | SINE | 836 |
| FR393260 | contained within HIS | HIST1H1E (+) | 0 | COSM1311866 | COSM1311866 | 0 | region is 835 bp dow | AluY (-) | SINE | 835 |
| FR133421 | contained within HIS | HIST1H1D (-) | 0 | region is 26 bp from | COSM161530 | 26 | region is 988 bp ups | AT_rich (+) | Low_complexity | 988 |
| FR379437 | contained within HIS | HIST1H2BH (+) | 0 | COSM1621291 | COSM1621291 | 0 | region is 391 bp dow | FLAM_C (-) | SINE | 391 |
| FR217228 | contained within HIS | HIST1H2BJ (-) | 0 | COSM1161614, COSI | COSM1161614 | 0 | region is 811 bp dow | AluSx (+) | SINE | 811 |
| FR197889 | contained within HIS | HIST1H2BJ (-) | 0 | region is 17 bp from | COSM3722041 | 17 | region is 991 bp dow | AluSx (+) | SINE | 991 |
| FR324841.1 | contained within HIS | HIST1H2AI (+) | 0 | COSM3720554 | COSM3720554 | 0 | region is 1091 bp ups | MER33 (+) | DNA | 1091 |
| FR324841_dup1.1 | contained within HIS | HIST1H2AJ (-) | 0 | COSM595470 | COSM595470 | 0 | region is 280 bp dow | (A)n (+) | Simple_repeat | 280 |
| FR279637 | contained within HIS | HIST1H1B (-) | 0 | COSM1719065 | COSM1719065 | 0 | region is 1191 bp do | AluJb (+) | SINE | 1191 |
| FR263507 | contained within HIS | HIST1H2AM (-) | 0 | COSM1161616 | COSM1161616 | 0 | region is 570 bp ups | AluJb (-) | SINE | 570 |
| FR207412 | contained within HIS | HIST1H2AM (-) | 0 | COSM1292272, COSI | COSM74904 | 0 | region is 642 bp ups | AluJb (-) | SINE | 642 |
| FR379437_dup1 | contained within HIS | HIST1H2BO (+) | 0 | COSM1254215, COSI | COSM741679 | 0 | region is 510 bp dow | AluSg4 (-) | SINE | 510 |
| FR312701 | contained within LIN | LINC01015 (+) | 0 | region is 25911 bp fr | COSM3250625 | 25911 | region is 2597 bp ups | MER4C (-) | DNA | 2597 |
| FR312701_dup1 | contained within LIN | LINC01015 (+) | 0 | region is 25911 bp fr | COSM3250625 | 25911 | region is 2597 bp ups | MER4C (-) | DNA | 2597 |
| FR312701_dup2 | contained within LIN | LINC01015 (+) | 0 | region is 25911 bp fr | COSM3250625 | 25911 | region is 2597 bp ups | MER4C (-) | DNA | 2597 |
| FR029085 | intron of TRIM26 (-) | TRIM26 (-) | 0 | region is 12363 bp fr | COSM165128 | 12363 | region is 210 bp ups | L1MC3 (+) | LINE | 210 |
| FR029085_dup1 | intron of TRIM26 (-) | TRIM26 (-) | 0 | region is 12363 bp fr | COSM165128 | 12363 | region is 210 bp ups | L1MC3 (+) | LINE | 210 |
| FR029085_dup2 | intron of TRIM26 (-) | TRIM26 (-) | 0 | region is 12363 bp fr | COSM165128 | 12363 | region is 210 bp ups | L1MC3 (+) | LINE | 210 |
| FR338575 | contained within HLA | HLA-B (-) | 0 | region is 678 bp from | COSM3410931 | 678 | region is 613 bp ups | L3 (-) | LTR | 613 |
| FR338575_dup1 | contained within HLA | HLA-B (-) | 0 | region is 678 bp from | COSM3410931 | 678 | region is 613 bp ups | L3 (-) | LTR | 613 |
| FR265415 | contained within HLA | HLA-B (-) | 0 | COSM1131924, COSI | COSM1443267 | 0 | region is 1774 bp do | L1MEf (-) | LINE | 1774 |
| FR265415_dup1 | contained within HLA | HLA-B (-) | 0 | COSM1131924, COSI | COSM1443267 | 0 | region is 1774 bp do | L1MEf (-) | LINE | 1774 |

| | | | | | | | | | | | |
|---|---|---|---|---|---|---|---|---|---|---|---|
| FR337999 | contained within SN( | SNORD84 (-) | 0 | region is 511 bp from | COSM1621424 | 511 | region is 151 bp dow | L1MDa (+) | LINE | 151 |
| FR337999_dup1 | contained within SN( | SNORD84 (-) | 0 | region is 511 bp from | COSM1621424 | 511 | region is 151 bp dow | L1MDa (+) | LINE | 151 |
| FR337999_dup2 | contained within SN( | SNORD84 (-) | 0 | region is 511 bp from | COSM1621424 | 511 | region is 151 bp dow | L1MDa (+) | LINE | 151 |
| FR291903 | contained within SN( | SNORD84 (-) | 0 | region is 511 bp from | COSM1621424 | 511 | region is 154 bp dow | L1MDa (+) | LINE | 154 |
| FR291903_dup1 | contained within SN( | SNORD84 (-) | 0 | region is 511 bp from | COSM1621424 | 511 | region is 154 bp dow | L1MDa (+) | LINE | 154 |
| FR291903_dup2 | contained within SN( | SNORD84 (-) | 0 | region is 511 bp from | COSM1621424 | 511 | region is 154 bp dow | L1MDa (+) | LINE | 154 |
| FR301922 | contained within SN( | SNORA38 (+) | 0 | region is 182 bp from | COSM1077604 | 182 | region is 151 bp dow | AluSp (-) | SINE | 151 |
| FR301922_dup1 | contained within SN( | SNORA38 (+) | 0 | region is 182 bp from | COSM1077604 | 182 | region is 151 bp dow | AluSp (-) | SINE | 151 |
| FR013752 | region is 10552 bp do | LINC01016 (-) | 10552 | region is 77771 bp fr | COSM1078179 | 77771 | region is 344 bp dow | MIR3 (-) | SINE | 344 |
| FR354968 | intron of LINC01016 ( | LINC01016 (-) | 0 | region is 91433 bp fr | COSM1078179 | 91433 | region is 421 bp upst | G-rich (+) | Low_complexity | 421 |
| FR208423 | intron of LINC01016 ( | LINC01016 (-) | 0 | region is 94647 bp fr | COSM1078179 | 94647 | region is 1122 bp do | L2a (+) | LINE | 1122 |
| FR357773 | intron of LINC01016 ( | LINC01016 (-) | 0 | region is 95304 bp fr | COSM1078179 | 95304 | region is 1779 bp do | L2a (+) | LINE | 1779 |
| FR366450 | contained within C6o | C6orf89 (+) | 0 | COSM1546429 | COSM1546429 | 0 | region is 126 bp dow | MER5C1 (+) | DNA | 126 |
| FR028930 | contained within ZFA | ZFAND3 (+) | 0 | region is 741 bp from | COSM1444181 | 741 | region is 2730 bp ups | HAL1 (+) | LINE | 2730 |
| FR381029 | contained within TB( | TBCC (-) | 0 | region is 20 bp from | COSM484083 | 20 | region is 608 bp upst | C-rich (+) | Low_complexity | 608 |
| FR008494.1 | contained within EEF | EEF1A1 (-) | 0 | COSM1600349, COSI | COSM1621917 | 0 | region is 1146 bp do | AluYc (+) | SINE | 1146 |
| FR102402.5 | contained within EEF | EEF1A1 (-) | 0 | COSM1445855, COSI | COSM1445855 | 0 | region is 2420 bp do | AluYc (+) | SINE | 2420 |
| FR073001.4 | contained within EEF | EEF1A1 (-) | 0 | region is 1 bp from C | COSM1445855 | 1 | region is 2448 bp do | AluYc (+) | SINE | 2448 |
| FR338565.5 | contained within EEF | EEF1A1 (-) | 0 | COSM1329544, COSI | COSM1329544 | 0 | region is 2715 bp do | AluYc (+) | SINE | 2715 |
| FR365184 | contained within BEI | BEND3 (-) | 0 | COSM739769 | COSM739769 | 0 | region is 1095 bp ups | L1ME1 (+) | LINE | 1095 |
| FR025321 | contained within SN( | SNORD100 (+) | 0 | region is 128 bp from | COSM3829088 | 128 | region is 693 bp upst | AluSx (-) | SINE | 693 |
| FR009646 | region is 101 bp upst | HBS1L (-) | 101 | region is 4352 bp fro | COSM3829113 | 4352 | region is 83 bp upstr | L2c (+) | LINE | 83 |
| FR072519.1 | intron of MTHFD1L (+ | MTHFD1L (+) | 0 | region is 7022 bp fro | COSM3430112 | 7022 | region is 347 bp dow | AluSx1 (+) | SINE | 347 |
| FR061350.1 | region is 151300 bp u | RGS17 (-) | 151300 | region is 64522 bp fr | COSM4160302 | 64522 | region is 308 bp dow | (TTC)n (+) | Simple_repeat | 308 |
| FR205900 | contained within RPS | RPS6KA2 (-) | 0 | region is 2140 bp fro | COSM3761647 | 2140 | region is 658 bp upst | L4 (+) | LTR | 658 |
| FR224235.1 | contained within ACT | ACTB (-) | 0 | COSM1451626 | COSM1451626 | 0 | region is 824 bp dow | (A)n (+) | Simple_repeat | 824 |
| FR054563 | region is 457 bp upst | C7orf26 (+) | 457 | region is 495 bp from | COSM3669751 | 495 | region is 430 bp upst | GC_rich (+) | Low_complexity | 430 |
| FR008494.2 | region is 10417 bp up | STEAP1B (-) | 10417 | region is 15860 bp fr | COSM1596936 | 15860 | region is 386 bp upst | L1MD3 (-) | LINE | 386 |
| FR066510.1 | region is 10435 bp up | STEAP1B (-) | 10435 | region is 15878 bp fr | COSM1596936 | 15878 | region is 404 bp upst | L1MD3 (-) | LINE | 404 |
| FR073001.5 | region is 11364 bp up | STEAP1B (-) | 11364 | region is 16807 bp fr | COSM1596936 | 16807 | region is 419 bp dow | L1MD3 (-) | LINE | 419 |
| FR338565.6 | region is 11523 bp up | STEAP1B (-) | 11523 | region is 16966 bp fr | COSM1596936 | 16966 | region is 260 bp dow | L1MD3 (-) | LINE | 260 |
| FR238720 | contained within TRA | TRA2A (-) | 0 | COSM1312981, COSI | COSM1312981 | 0 | region is 466 bp dow | AluJb (-) | SINE | 466 |
| FR184831.1 | region is 66885 bp do | HIBADH (-) | 66885 | region is 67660 bp fr | COSM600762 | 67660 | region is 1727 bp do | L1ME4a (-) | LINE | 1727 |
| FR025280 | intron of JAZF1 (-) | JAZF1 (-) | 0 | region is 37905 bp fr | COSM1088925 | 37905 | region is 930 bp upst | L2a (-) | LINE | 930 |
| FR255470 | contained within POI | POLR2J4 (-) | 0 | region is 6934 bp fro | COSM453088 | 6934 | region is 499 bp upst | AluSg (-) | SINE | 499 |
| FR072519.2 | intron of NUDCD3 (-) | NUDCD3 (-) | 0 | region is 17035 bp fr | COSM232919 | 17035 | region is 68 bp down | (GAAAA)n (+) | Simple_repeat | 68 |
| FR110343 | region is 323 bp upst | TMED4 (-) | 323 | region is 397 bp from | COSM453117 | 397 | region is 267 bp upst | AluJb (+) | SINE | 267 |
| FR330790 | region is 493 bp upst | TMED4 (-) | 493 | region is 567 bp from | COSM453117 | 567 | region is 94 bp upstr | AluJb (+) | SINE | 94 |
| FR012473 | contained within SN( | SNORA5A (-) | 0 | region is 71 bp from | COSM1090060 | 71 | region is 2063 bp ups | AluSx (+) | SINE | 2063 |
| FR328549 | region is 6366 bp ups | ZNF736 (+) | 6366 | region is 28822 bp fr | COSM1623031 | 28822 | region is 249 bp upst | AluSc8 (-) | SINE | 249 |
| FR115701 | region is 23165 bp do | MDH2 (+) | 23165 | region is 5548 bp fro | COSM3703275 | 5548 | region is 753 bp upst | AluSx (-) | SINE | 753 |
| FR125672 | region is 33293 bp do | COL1A2 (+) | 33293 | region is 34159 bp fr | COSM3715856 | 34159 | region is 22 bp down | L1PA15 (-) | LINE | 22 |
| FR140858 | contained within MIR | MIR106B (-) | 0 | region is 125 bp from | COSM1214734 | 125 | region is 693 bp dow | Tigger4b (-) | DNA | 693 |
| FR255470_dup1 | contained within POI | POLR2J (-) | 0 | region is 751 bp from | COSM1083506 | 751 | region is 1180 bp ups | AluSq2 (+) | SINE | 1180 |
| FR255470_dup2 | contained within POI | POLR2J3 (-) | 0 | region is 10893 bp fr | COSM1488088 | 10893 | region is 499 bp upst | AluSg (-) | SINE | 499 |
| FR255470_dup3 | contained within POI | POLR2J2 (-) | 0 | region is 20 bp from | COSM136622 | 20 | region is 499 bp upst | AluSg (-) | SINE | 499 |
| FR141628 | contained within GC( | GCC1 (-) | 0 | COSM2157039, COSI | COSM452271 | 0 | region is 3485 bp do | L2c (-) | LINE | 3485 |
| FR243752 | contained within GC( | GCC1 (-) | 0 | region is 389 bp from | COSM1447850 | 389 | region is 466 bp dow | L2c (-) | LINE | 466 |
| FR348796.1 | contained within IMP | IMPDH1 (-) | 0 | region is 12 bp from | COSM1548339 | 12 | region is 196 bp dow | MER103C (+) | DNA | 196 |
| FR381169 | contained within MIR | MIR182 (-) | 0 | region is 15266 bp fr | COSM461681 | 15266 | region is 178 bp upst | C-rich (+) | Low_complexity | 178 |
| FR111293 | contained within TMI | TMEM209 (-) | 0 | region is 1286 bp fro | COSM1085498 | 1286 | region is 436 bp dow | AluSz (-) | SINE | 436 |
| FR281234.1 | intron of CHCHD3 (-) | CHCHD3 (-) | 0 | region is 179 bp from | COSM160775 | 179 | region is 1184 bp do | (T)n (+) | Simple_repeat | 1184 |

| | | | | | | | | | | | |
|---|---|---|---|---|---|---|---|---|---|---|---|
| FR233520.6 | intron of DGKI (-) | DGKI (-) | 0 | region is 7767 bp fro | COSM3394553 | 7767 | region is 131 bp dow | Tigger3b (-) | DNA | 131 |
| FR337428 | contained within KIA | KIAA1147 (-) | 0 | region is 4066 bp fro | COSM1212078 | 4066 | region is 2374 bp dov | Tigger4a (+) | DNA | 2374 |
| FR266905.2 | region is 13905 bp do | TAS2R5 (+) | 13905 | region is 14019 bp fr | COSM1086335 | 14019 | region is 232 bp from | tRNA-Leu-TTA(m) (-) | tRNA | 232 |
| FR206006 | region is 13907 bp do | TAS2R5 (+) | 13907 | region is 14021 bp fr | COSM1086335 | 14021 | region is 232 bp upst | L1MB7 (+) | LINE | 232 |
| FR015567 | region is 13908 bp do | TAS2R5 (+) | 13908 | region is 14022 bp fr | COSM1086335 | 14022 | region is 232 bp upst | L1MB7 (+) | LINE | 232 |
| FR015567_dup1 | contained within MTF | MTRNR2L6 (+) | 0 | region is 21545 bp fr | COSM1635162 | 21545 | region is 623 bp upst | MER2 (+) | DNA | 623 |
| FR206006_dup1 | contained within MTF | MTRNR2L6 (+) | 0 | region is 21545 bp fr | COSM1635162 | 21545 | region is 622 bp upst | MER2 (+) | DNA | 622 |
| FR266905_dup1.1 | contained within MTF | MTRNR2L6 (+) | 0 | region is 21548 bp fr | COSM1635162 | 21548 | region is 620 bp upst | MER2 (+) | DNA | 620 |
| FR353662 | region is 1418 bp ups | FAM115C (+) | 1418 | region is 83546 bp fr | COSM1086816 | 83546 | region is 66 bp upstr | AluSx (-) | SINE | 66 |
| FR338565_dup2 | intron of TPK1 (-) | TPK1 (-) | 0 | region is 416 bp from | COSM1548802 | 416 | region is 540 bp upst | AluJr (+) | SINE | 540 |
| FR090905 | region is 118942 bp u | CNTNAP2 (+) | 118942 | region is 119530 bp f | COSM3669704 | 119530 | region is 47 bp down | L2a (-) | LINE | 47 |
| FR365501 | region is 118943 bp u | CNTNAP2 (+) | 118943 | region is 119531 bp f | COSM3669704 | 119531 | region is 48 bp down | L2a (-) | LINE | 48 |
| FR099055 | contained within ABC | ABCB8 (+) | 0 | region is 81 bp from | COSM1449250 | 81 | region is 498 bp dow | LTR5_Hs (+) | LTR | 498 |
| FR197104 | contained within RBI | RBM33 (+) | 0 | region is 1655 bp fro | COSM1622633 | 1655 | region is 2038 bp ups | T-rich (+) | Low_complexity | 2038 |
| FR001130 | region is 293 bp upst | DNAJB6 (+) | 293 | region is 21869 bp fr | COSM1699645 | 21869 | region is 136 bp dow | GC_rich (+) | Low_complexity | 136 |
| FR296497 | contained within CTS | CTSB (-) | 0 | region is 852 bp from | COSM177681 | 852 | region is 247 bp dow | AluSx1 (+) | SINE | 247 |
| FR404492 | contained within CTS | CTSB (-) | 0 | region is 3 bp from C | COSM485996 | 3 | region is 1623 bp dov | AluSx1 (+) | SINE | 1623 |
| FR184567 | contained within CTS | CTSB (-) | 0 | COSM1202633 | COSM1202633 | 0 | region is 1361 bp dov | MER5B (-) | DNA | 1361 |
| FR288536 | region is 4472 bp dov | HMBOX1 (+) | 4472 | region is 6075 bp fro | COSM1098958 | 6075 | region is 1249 bp dov | (T)n (+) | Simple_repeat | 1249 |
| FR086093.3 | contained within RPS | RPS20 (-) | 0 | region is 20 bp from | COSM3766779 | 20 | region is 1107 bp dov | AT_rich (+) | Low_complexity | 1107 |
| FR132045 | contained within SNO | SNORD54 (-) | 0 | region is 164 bp from | COSM221172 | 164 | region is 1272 bp dov | AT_rich (+) | Low_complexity | 1272 |
| FR136623 | contained within SNO | SNORD54 (-) | 0 | region is 164 bp from | COSM221172 | 164 | region is 1275 bp dov | AT_rich (+) | Low_complexity | 1275 |
| FR338747.1 | intron of CPA6 (-) | CPA6 (-) | 0 | region is 38697 bp fr | COSM3779316 | 38697 | region is 225 bp dow | L2 (-) | LINE | 225 |
| FR038165 | intron of CPA6 (-) | CPA6 (-) | 0 | region is 38697 bp fr | COSM3779316 | 38697 | region is 225 bp dow | L2 (-) | LINE | 225 |
| FR357791.4 | contained within FAE | FABP5 (+) | 0 | COSM197164 | COSM197164 | 0 | region is 132 bp dow | GC_rich (+) | Low_complexity | 132 |
| FR089602 | region is 10929 bp up | LINC00964 (+) | 10929 | region is 44571 bp fr | COSM1454740 | 44571 | region is 36 bp upstr | MamGypLTR1d (+) | LTR | 36 |
| FR179095 | contained within JRK | JRK (-) | 0 | region is 16551 bp fr | COSM4162717 | 16551 | region is 515 bp dow | Tigger10 (-) | DNA | 515 |
| FR342305 | contained within PLE | PLEC (-) | 0 | COSM3698904, COSI | COSM3698908 | 0 | region is 3004 bp ups | (CTG)n (+) | Simple_repeat | 3004 |
| FR292218 | intron of LRRC14 (+) | LRRC14 (+) | 0 | region is 1032 bp fro | COSM1552281 | 1032 | region is 101 bp upst | MIRb (+) | SINE | 101 |
| FR266905.3 | intron of JAK2 (+) | JAK2 (+) | 0 | region is 2856 bp fro | COSM403070 | 2856 | region is 228 bp from | tRNA-Leu-TTA(m) (+) | tRNA | 228 |
| FR057472 | contained within KDI | KDM4C (+) | 0 | region is 27961 bp fr | COSM1109557 | 27961 | region is 111 bp dow | AluJb (-) | SINE | 111 |
| FR360021 | region is 215098 bp d | CAAP1 (-) | 215098 | region is 216782 bp f | COSM1497088 | 216782 | region is 237 bp upst | (TC)n (+) | Simple_repeat | 237 |
| FR266905_dup1.2 | region is 14467 bp do | PTENP1 (-) | 14467 | region is 3340 bp fro | COSM1108374 | 3340 | region is 235 bp dow | LSU-rRNA_Hsa (+) | rRNA | 235 |
| FR348796.2 | region is 7313 bp ups | DCAF12 (-) | 7313 | region is 7699 bp fro | COSM608683 | 7699 | region is 627 bp upst | (CCG)n (+) | Simple_repeat | 627 |
| FR207372 | intron of LINC01507 ( | LINC01507 (+) | 0 | region is 146934 bp f | COSM4149501 | 146934 | region is 33 bp upstr | L1MB4 (-) | LINE | 33 |
| FR097576 | region is 669 bp dow | SPATA31D5P (+) | 669 | region is 68204 bp fr | COSM400115 | 68204 | region is 672 bp upst | (CAAAA)n (+) | Simple_repeat | 672 |
| FR097576_dup1 | region is 670 bp dow | SPATA31D4 (+) | 670 | region is 53132 bp fr | COSM400115 | 53132 | region is 672 bp upst | (CAAAA)n (+) | Simple_repeat | 672 |
| FR072386 | contained within MIR | MIRLET7A1 (+) | 0 | region is 68036 bp fr | COSM1111336 | 68036 | region is 847 bp upst | L1M4 (-) | LINE | 847 |
| FR210409 | contained within MIR | MIRLET7F1 (+) | 0 | region is 68427 bp fr | COSM1111336 | 68427 | region is 1238 bp ups | L1M4 (-) | LINE | 1238 |
| FR144444_dup1 | contained within FAI | FAM225B (-) | 0 | region is 47853 bp fr | COSM1553666 | 47853 | region is 500 bp dow | AluSx1 (-) | SINE | 500 |
| FR144444 | contained within FAI | FAM225A (+) | 0 | region is 43030 bp fr | COSM1553666 | 43030 | region is 500 bp dow | AluSx1 (+) | SINE | 500 |
| FR344475_dup1 | contained within FAI | FAM225A (+) | 0 | region is 40297 bp fr | COSM1553666 | 40297 | region is 143 bp dow | MLT1A0 (-) | LTR | 143 |
| FR158689 | contained within MIR | MIR600HG (-) | 0 | region is 9437 bp fro | COSM1624518 | 9437 | region is 1155 bp ups | MER58A (+) | DNA | 1155 |
| FR275856 | region is 27231 bp up | PRRC2B (+) | 27231 | region is 27341 bp fr | COSM752516 | 27341 | region is 94 bp upstr | L2a (-) | LINE | 94 |
| FR136216_dup1 | intron of PRRC2B (+) | SNORD62B (+) | 0 | region is 643 bp from | COSM3699489 | 643 | region is 1032 bp dov | L1ME3D (-) | LINE | 1032 |
| FR233730 | contained within SNO | SNORD62B (+) | 0 | region is 643 bp from | COSM3699489 | 643 | region is 1033 bp dov | L1ME3D (-) | LINE | 1033 |
| FR136216 | intron of PRRC2B (+) | SNORD62B (+) | 0 | region is 891 bp from | COSM3847894 | 891 | region is 179 bp dow | L2 (+) | LINE | 179 |
| FR233730_dup1 | contained within SNO | SNORD62B (+) | 0 | region is 892 bp from | COSM3847894 | 892 | region is 179 bp dow | L2 (+) | LINE | 179 |
| FR194863 | contained within PRF | PRRC2B (+) | 0 | region is 292 bp from | COSM1554236 | 292 | region is 2174 bp ups | MER103C (-) | DNA | 2174 |
| FR066510.2 | region is 9906 bp ups | GTF3C5 (+) | 9906 | region is 10257 bp fr | COSM1460755 | 10257 | region is 405 bp upst | (TAA)n (+) | Simple_repeat | 405 |
| FR372325 | contained within SNO | SNORD36C (+) | 0 | region is 191 bp from | COSM1460793 | 191 | region is 600 bp dow | AluSx (-) | SINE | 600 |
| FR258622 | contained within SEC | SEC16A (-) | 0 | region is 15 bp from | COSM1461085 | 15 | region is 397 bp upst | AluSx (+) | SINE | 397 |

| | | | |
|---|---|---|---|
| FR162144 | intron of ABCA2 (-) | ABCA2 (-) | 0 |
| FR376350.3 | intron of APOO (-) | APOO (-) | 0 |
| FR348796.3 | region is 72393 bp do | LOC101927476 (+) | 72393 |
| FR042730.3 | region is 37143 bp do | LINC01186 (-) | 37143 |
| FR182987 | contained within MIR | MIR532 (+) | 0 |
| FR378984 | contained within MIR | MIR532 (+) | 0 |
| FR203981 | region is 22294 bp do | KDM5C (-) | 22294 |
| FR324515 | intron of ZC4H2 (-) | ZC4H2 (-) | 0 |
| FR213473 | intron of ZC4H2 (-) | ZC4H2 (-) | 0 |
| FR268871 | intron of ZC4H2 (-) | ZC4H2 (-) | 0 |
| FR218180.2 | region is 110303 bp d | HMGN5 (-) | 110303 |
| FR102402.8 | intron of DACH2 (+) | DACH2 (+) | 0 |
| FR073001.7 | intron of DACH2 (+) | DACH2 (+) | 0 |
| FR152361.1 | region is 3810 bp dov | MIR2114 (+) | 3810 |
| FR264146 | region is 288273 bp d | TTTY23B (+) | 288273 |

| | | |
|---|---|---|
| region is 58 bp from | COSM1107004 | 58 |
| None | None | 0 |
| None | None | 0 |
| None | None | 0 |
| None | None | 0 |
| None | None | 0 |
| None | None | 0 |
| None | None | 0 |
| None | None | 0 |
| None | None | 0 |
| None | None | 0 |
| None | None | 0 |
| None | None | 0 |
| None | None | 0 |
| None | None | 0 |

| | | | |
|---|---|---|---|
| region is 1498 bp dov | (CCCCG)n (+) | Simple_repeat | 1498 |
| region is 141 bp upst | FLAM_C (+) | SINE | 141 |
| region is 684 bp dow | L1MB4 (-) | LINE | 684 |
| region is 425 bp dow | MER4D0 (-) | DNA | 425 |
| region is 219 bp dow | MIR (+) | SINE | 219 |
| region is 256 bp dow | MIR (+) | SINE | 256 |
| region is 1 bp upstre | MLT2B4 (-) | LTR | 1 |
| tRNA-Gly-GGY (-) | tRNA-Gly-GGY (-) | tRNA | 0 |
| contained within tRN | tRNA-Gly-GGY (-) | tRNA | 0 |
| tRNA-Gly-GGY (-) | tRNA-Gly-GGY (-) | tRNA | 0 |
| region is 2307 bp ups | MIR3 (+) | SINE | 2307 |
| region is 623 bp dow | MER41B (-) | DNA | 623 |
| region is 599 bp dow | MER41B (-) | DNA | 599 |
| region is 989 bp upst | L1ME3A (+) | LINE | 989 |
| region is 478 bp dow | MER45C (+) | DNA | 478 |
